# Manipulating Ferroelectric Topological Polar Structures with Twisted Light

**DOI:** 10.1002/adma.202415231

**Published:** 2025-06-06

**Authors:** Nimish P. Nazirkar, Viet Tran, Pascal Bassène, Atoumane Ndiaye, Julie Barringer, Jie Jiang, Wonsuk Cha, Ross Harder, Jian Shi, Moussa N'Gom, Edwin Fohtung

**Affiliations:** ^1^ Department of Materials Science and Engineering Rensselaer Polytechnic Institute (RPI) Troy NY 12180 USA; ^2^ Department of Physics Applied Physics, and Astronomy, RPI Troy NY 12180 USA; ^3^ Shirley Ann Jackson Ph.D. Center for Biotechnology and Interdisciplinary Studies RPI Troy NY 12180 USA; ^4^ Advanced Photon Source Argonne National Laboratory Lemont IL 60439 USA

**Keywords:** bragg coherent x‐ray diffractive imaging, data storage, energy harvesting, information processing, twisted and structured light, Orbital angular momentum, Quazi‐2D Ferroelectrics, Twisted light Raman Spectroscopy, Polar Vortices, Bloch Points and Anti Bloch Points, Merons and Hopfions

## Abstract

The dynamic control of non‐equilibrium states represents a central challenge in condensed matter physics. While intense terahertz fields drive metal‐insulator transitions and ferroelectricity via soft phonon modes, recent theory suggests that twisted light with orbital angular momentum (OAM) offers a distinct route to manipulate ferroelectric order and stabilize topological excitations including skyrmions, vortices, and Hopfions. Control of ferroelectric polarization in quasi‐2D CsBiNb_2_O_7_ (CBNO) is demonstrated using non‐resonant twisted ultra‐violet (UV) light (375 nm, 800 THz). Combining in situ X‐ray Bragg coherent diffractive imaging (BCDI), twisted optical Raman spectroscopy, and density functional theory (DFT), three‐dimensional (3D) ionic displacements, strain fields, and polarization changes are resolved in single crystals. Operando measurements reveal light‐induced strain hysteresis under twisted light–a hallmark of nonlinear, history‐dependent ferroelastic switching driven by OAM. Discrete, irreversible domain transitions emerge as the topological charge ℓ is cycled, stabilizing non‐trivial domain textures including vortex‐antivortex pairs, Bloch/anti‐Bloch points, and merons. These persist after OAM removal, indicating a memory effect. Competing mechanisms are discussed, including multiphoton absorption, strain‐mediated polarization switching, and defect‐wall interactions. The findings establish structured light as a tool for deterministic, reversible control of ferroic states, enabling optically reconfigurable non‐volatile devices.

## Graphical abstract

We demonstrate dynamic control of ferroelectric order in quasi‐2D CsBiNb2O7 using twisted ultraviolet light carrying orbital angular momentum. Our approach harnesses non‐resonant multiphoton absorption and induced strain to modulate topological of ferroelectric polarization textures. In‐situ X‐ray coherent imaging and Raman spectroscopy reveal reversible, nanoscale polarization transitions, enabling efficient stabilization of topological solitons and paving the way for novel optoelectronic devices.

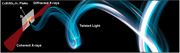

## 1 Introduction

In recent years, there has been a surge in condensed matter research toward nontrivial topologically‐protected dipolar textures. These textures have predominantly been found in bulk materials, nanocrystals, and epitaxial heterostructures, where precise electrostatic, magnetostatic, and elastic boundary conditions are enforced on crystalline ferroelectric (FE) and magnetic materials. Since the prediction of flux‐closure domain structures in magnetic films in the 1940s,^[^ ^1^, ^2^ ^]^ parallels have been drawn in predicting and observing real‐space topologically‐protected dipolar textures such as flux‐closures,^[^ ^3^ ^]^ center domains,^[^ ^4^ ^]^ vortices,^[^ ^5^, ^6^ ^]^ skyrmions,^[^ ^7^ ^]^ merons,^[^ ^8^ ^]^ bubbles,^[^ ^9^ ^]^ and hopfions^[^ ^10^ ^]^ in ferroelectrics. These topological structures, especially in ferroelectric materials, hold potential for applications in high‐density information storage, thin‐film capacitors, actuators, and other electronic devices.^[^ ^1^, ^2^, ^8^ ^]^ Furthermore, their inherent stability and rich interfacial phenomena offer promising avenues for multifunctional device architectures where electronic, magnetic, and mechanical responses can be synergistically exploited.

Advances in theory and experimental investigations have focused on the stabilization and observation of polar textures in ferroelectric systems. Studies have reported phenomena such as negative capacitance and the emergence of chirality in polar vortices and skyrmions, originating from sequences of achiral materials, using resonant soft X‐ray diffraction‐based circular dichroism and optical measurements.^[^ ^11^, ^12^, ^13^, ^14^ ^]^ Phase field simulations^[^ ^15^ ^]^ and aberration‐corrected scanning transmission electron microscopy have further shown that polar Bloch points (BPs) can be stabilized in substrate‐induced tensile‐strained ultrathin FE PbTiO_3_ films. However, experimentally observing and manipulating these textures in a nondestructive and volumetric manner remains a significant challenge. Addressing these challenges is critical, as it would enable the precise control of domain architectures and the realization of devices with enhanced performance and novel functionalities.

In this work, we address these challenges by demonstrating the stabilization and manipulation of polar BPs in two‐dimensional (2D) FE flakes using twisted light (TL) illumination and advanced imaging techniques. Specifically, the use of sub‐band gap 375 nm twisted UV light enables localized interactions with mid‐gap states and multiphoton processes, dynamically modulating polarization textures and inducing strain inhomogeneities in free‐standing CsBiNb_2_O_7_ (CBNO) flakes. Additionally, the nontrivial phase profile of TL allows for spatially resolved excitation, offering unprecedented control over the induced ferroelectric responses. By combining in situ X‐ray Bragg coherent diffractive imaging (BCDI)^[^ ^5^, ^16^, ^17^ ^]^ and optical Raman spectroscopy, we reconstruct 3D atomic displacements and strain maps, revealing reversible transitions from BPs to merons and back to BPs. Our observations advance the fundamental understanding of ferroelectric polar textures and open pathways for applications in next‐generation spintronics technologies.

**Figure 1:**
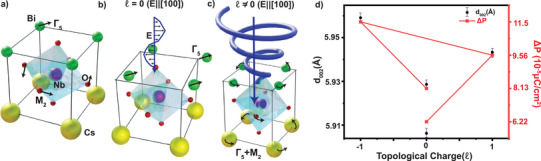
Schematic of the manipulation of FE using vortex field from twisted light and its experimental detection. a) The CsBiNb_2_O_7_(CBNO) crystal unit responsible for FE, shown in the absence of twisted light. When driven by a plane‐polarized 375 nm UV field in a Laguerre‐Gaussian (LG_ℓ_) mode carrying orbital angular momentum (OAM) quantized at ℓℏ per photon, the twisted light interacts with mid‐gap states and defect‐mediated pathways through non‐resonant mechanisms, inducing localized strain gradients and multiphoton absorption effects. This interaction dynamically modulates ionic displacements and ferroelectric (FE) polarization textures. b,c) Twisted UV light topological charges ℓ = 0 and ℓ ≠ 0 create quasi‐static strain fields that couple to vibrational modes, driving polarization changes. b) Zone‐center FE modes and c) zone‐boundary octahedral tilting modes respond to the localized strain and OAM‐induced torques, dynamically modulating the symmetry and polarization textures. d) Experimentally observed changes in polarization and atomic displacements along the [002] direction in CBNO arising due to the strain and phonon modes depicted in (b and c). These observations confirm that twisted UV light stabilizes non‐equilibrium topological phases via defect‐coupled and strain‐mediated mechanisms.

The microscopic origin of twisted UV light‐induced strain heterogeneity in CBNO remains an open question, though several interrelated mechanisms are likely at play. Our observations suggest that non‐linear optical processes–particularly multi‐photon absorption facilitated by the structured wavefront of vortex beams–induce localized carrier generation, which perturbs the lattice and gives rise to strain patterns observed via Bragg Coherent Diffractive Imaging (BCDI) as shown in **Figure** **1** **d**. Additionally, selective sub‐bandgap excitation of shallow defect states may couple to polar phonon modes, amplifying local distortions under twisted light.^[^ ^18^, ^19^ ^]^ Strain‐sensitive Rashba–Dresselhaus interactions also emerge as a crucial component, linking ferroelectric polarization, spin orbit coupling, and lattice deformations.^[^ ^20^, ^21^, ^22^ ^]^ When TL with finite OAM impinges upon CBNO, it exerts a torque that influences the material's polarization state. Due to the strong spin orbit coupling (SOC) in this material, the OAM of the light can be effectively transferred to its spin texture, directly manipulating the spin states via the polarization texture. Consequently, the electronic band structure, including modifications in the Rashba spin splitting, can be dynamically tuned, enabling selective control over the spin texture. Furthermore, the handedness and magnitude of the OAM serve as additional degrees of freedom, allowing for deterministic tailoring of both polarization and spin configurations. This approach offers a dual control scheme in which the spatial (real‐space) and momentum‐space properties can be concurrently engineered.

By exploiting the interplay between Γ‐point zone‐center and M‐point zone‐boundary phonons, ^[^ ^20^ ^]^ we reveal phase transitions that stabilize polar vortex loops and Z_2_ × Z_4_ topological textures in CBNO.

Our results demonstrate that TL can dynamically modulate ferroelectricity by transferring orbital angular momentum to induce ionic displacements and polarization changes, as illustrated in Figure 1. The unique ability of TL to impart non‐uniform electric fields and strain gradients without electrodes offers a transformative approach for studying and manipulating ferroelectric systems. These findings emphasize the role of sub‐bandgap excitation pathways in driving polarization modulations and strain dynamics, paving the way for the development of ultrafast non‐volatile memory switches and other optoelectronic devices. Overall, our work not only deepens the understanding of the intricate coupling between light and matter in ferroelectric systems but also sets the stage for future explorations into the integration of photonic and spintronic functionalities in advanced materials.

## 2 Results

To conduct the in situ TL BCDI experiment, we first synthesized free‐standing CBNO 2D nanoflakes using a polymer precursor method.^[^ ^23^ ^]^ These nanoflakes were then transferred onto the sample holder (see Experimental Section and Figures S1 and S2, Supporting Information).

TL, also known as optical vortices or light carrying OAM, consists of electromagnetic fields^[^ ^24^, ^25^ ^]^ with one or more singularities where the phase^[^ ^24^ ^]^ is undefined, causing the intensity to vanish. The discovery of TL beams in visible light produced in free‐space^[^ ^24^, ^26^ ^]^ has generated significant interest across various scientific disciplines, including quantum information processing,^[^ ^27^ ^]^ optical tweezers, super‐resolution microscopy ^[^ ^28^ ^]^, and telecommunications.^[^ ^29^, ^30^ ^]^

**Figure 2:**
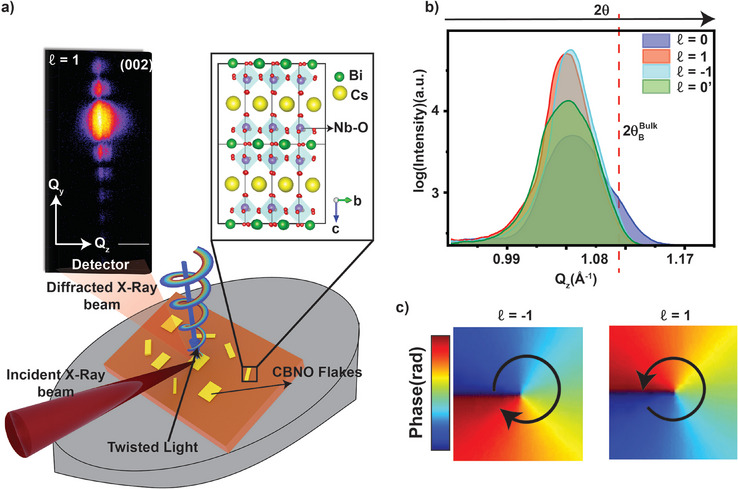
In situ Bragg coherent diffractive imaging experiment. a) Coherent x‐rays (red) are incident on a nanoflake (yellow) containing polarization vortices and FE domains subjected to twisted UV light (Red‐yellow‐blue) illumination. A schematic of a Dion‐Jacobson layered oxide CBNO crystal which has been shown to harbor FE with high Curie temperature and large in‐plane polarization is used as a model system to study twisted light control of atomic displacements, microscopic FE polarization and domain structure with near‐atomic resolution observed as (b) shifts and broadening of the recorded Bragg peak intensity. Simulated phase of twisted UV lights with topological charges (ℓ) of 1 and −1, carrying OAM quantized in units of ℓℏ per photon (c). In our experiments, the diffracted X‐rays collected at the detector plane carry information about the 3D electron density and atomic displacement fields within the nanoflake that allows us to monitor recorded X‐ray intensity variations near the Bragg spot during a cyclical application of light topological charge from ℓ = 0, 1, −1, and back to 0.

**Figure** **2**c illustrates exemplary phase and 2D intensity profiles (see Figures S4 and S31, Supporting Information) in planes perpendicular to the propagation direction (*z*‐direction) for the Laguerre‐Gaussian (LG_ℓ_) mode of TL used in this experiment. LG beams are solutions to the paraxial wave equation,^[^ ^24^ ^]^ and in the lowest order of the paraxial approximation, the electric and magnetic fields, as well as the vector potential, are purely transverse. Using the Lorenz gauge, the longitudinal component of the electric field (see Section S7 and Figures S4– S7, Supporting Information) can be expressed in terms of a scalar potential as:

(1) $$E_{z}\left(r,t\right)=-\partial_{t}\Phi \left(r,t\right)=-i\omega ce^{i(\mathrm{kz}-\omega t)}A_{0}\cdot \nabla_{⊥}\Pi \left(r\right)$$

where *E*_*z*_ is the longitudinal component of the electric field, Φ is the scalar potential, *c* is the speed of light, *k* is the wave number, ω is the angular frequency, **A**_0_ is the vector potential amplitude, ∇_⊥_ is the transverse gradient, and Π(**r**) is the complex amplitude of the LG beam, expressed as Π(**r**) = Π_0_(*r*, *z*)exp (*i*ℓϕ).

In our in situ BCDI setup, we utilized a spatial light modulator (SLM) (more details in Experimental Section) to focus a 375 nm continuum (with constant beam power over time) of LG beams with varied topological charge ℓ normal to the in‐plane direction ([100]‐direction) of our CBNO sample (see Figure 1). We generated a spatially inhomogeneous TL electric field **E**_OAM_ (2) and measured its intensity profile. For full derivation, see Figures S4– S6 and Note S7 (Supporting Information).

(2) $$E_{\text{OAM}}\left(\rho ,t\right)=\left\{\begin{array}{cc} E{\left(\frac{\sqrt{2}\rho }{w}\right)}^{\left|\ell \right|}e^{-\frac{\rho^{2}}{w^{2}}}\left(\cos\left(\ell \phi -\omega t\right)e_{x}-\sigma \sin\left(\ell \phi -\omega t\right)e_{y}\right), & \text{for}\;0\leq t\leq T\\ 0, & \text{for}\;t>T \end{array}\right.$$

**Figure 3:**
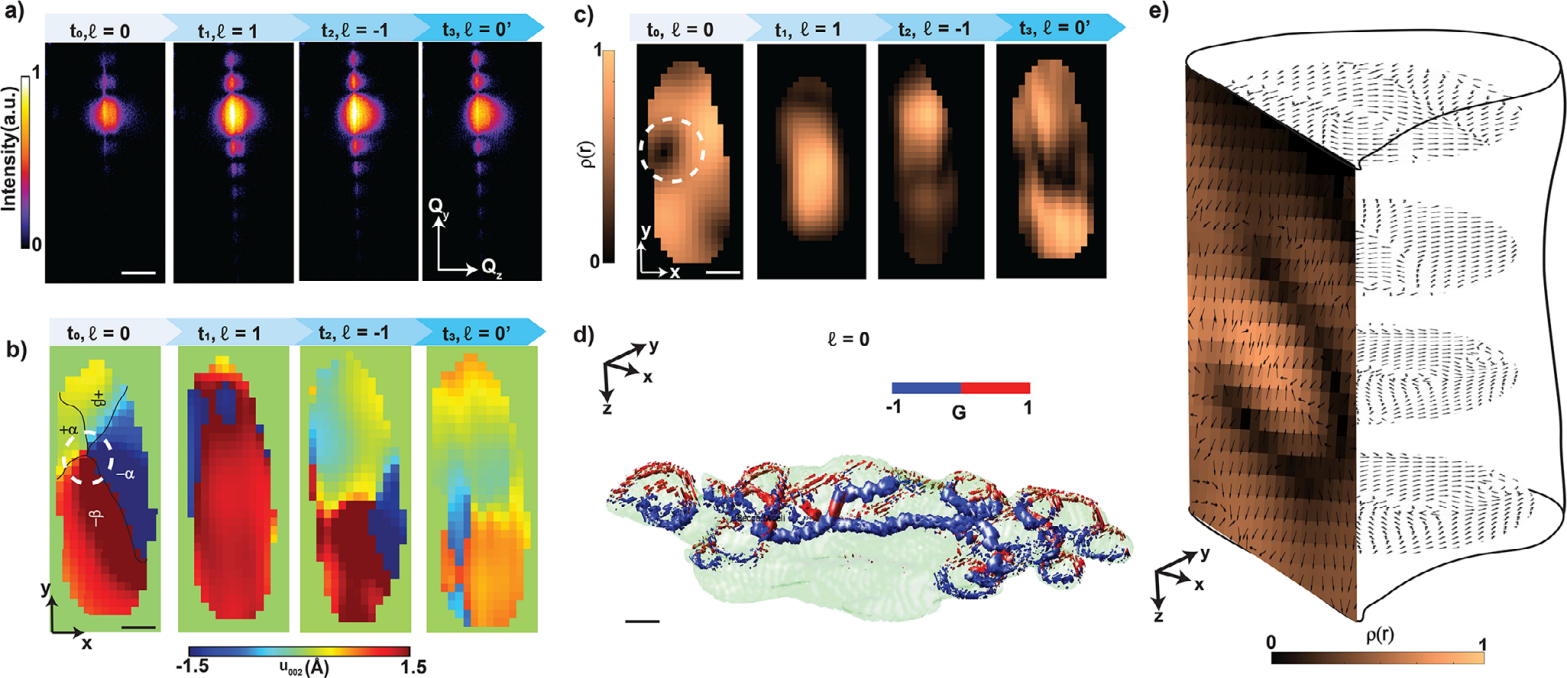
Real‐space topologically‐protected polar structures under a vortex‐light field. a) 2D slices from the recorded 3D coherent diffraction patterns for the (002) Bragg peak, used to measure the time evolution of the (b) FE displacement field during twisted light‐induced structural changes. The polarization vortex core is identified at time *t*_0_ under LG_ℓ_ beam illumination of ℓ = 0 (zero OAM). As time progresses to *t*_3_, changes in the displacement field are observed due to the nucleation of a vortex‐antivortex pair. c) A 2D map showing the evolution of the central cut of a Bloch point in the freestanding CBNO. d) The magnitude of the toroidal moment density under LG_ℓ_ beam of ℓ = 0. e) A rendering at time *t*_0_ of CBNO under a TL electric field of ℓ = 0, illustrating a vortex spanning the volume of the crystal along the *z* −direction. Each cross‐section perpendicular to the *z* − direction contains a vortex texture, and the cut through the *y* − direction shows a bloch‐like behavior around the vortex core. The scale bar in (a) represents 0.01 Å^−1^ while that in (b), (c), and (d) represents 60 nm.

In Equation (2), *T* is the time during which the CBNO nanoflake is under LG_ℓ_ beam illumination. At the time *t* = 0, we switched on the TL UV beam and, with the aid of the SLM and the neutral density filter, selected TL electric field polarization(σ = 0) components **e**_*x*_ and **e**_*y*_ and magnitude such that **E**_∥_[100] as shown in Figure 1. We aligned the X‐ray detector and CBNO crystal to satisfy the Bragg condition for the *d*_002_ inter‐atomic spacing of the (002) Bragg spot, as shown in **Figure** **3**. For a duration *T*, we measured 3D diffraction near the (002) peak for beam topological charge ℓ = 0. This procedure was repeated cyclically for ℓ = 0, 1, −1, and back to 0 (see Figure 3a). During the experiment, we observed TL‐induced structural changes by tracking the characteristic asymmetrical behavior in diffraction patterns (see Figures 1b and 3a).

X‐rays scattered by a single CBNO nanoflake satisfying the (002) Bragg condition were recorded on an area detector (see Figure 3a; and Section S11, Supporting Information). The experimental geometry, combined with the random orientation of the exfoliated nanoflakes, ensured that the (002) Bragg reflections from separate particles were well separated, allowing individual reflections to be isolated on the detector. The nanoflake was kept under continuous coherent TL UV illumination before the imaging experiment. From the coherent diffraction data, we reconstructed both the 3D distribution of the displacement field and the electron density (ρ(*x*, *y*, *z*)), along [002], *u*_002_(*x*, *y*, *z*), in an individual nanoflake with 14 nm spatial resolution (see Figures S15– S17, Supporting Information).

Figure 3b shows a cross‐section of the 3D displacement field [*u*_002_(*x*, *y*, *z* = *z*_0_)] in the FE nanoflake. The [002] direction is approximately along the *z* −axis, while the X‐ray beam is almost parallel to the *x* −axis. Depending on the topological polar structure type, the angular distribution will have distinct features associated with a topological invariant known as the topological FE charge winding number:

(3) $$\begin{array}{c} Q=\frac{1}{8\pi }\int dA_{i}\varepsilon_{ijk}\frac{\sum_{\kappa \alpha }Z_{\kappa ,\alpha }^{*}u_{\kappa \alpha }\left(r\right)}{\left|\sum_{\kappa \alpha }Z_{\kappa ,\alpha }^{*}u_{\kappa \alpha }\left(r\right)\right|}\\ \cdot \left[\partial_{j}\left(\frac{\sum_{\kappa \beta }Z_{\kappa ,\beta }^{*}u_{\kappa \beta }\left(r\right)}{\left|\sum_{\kappa \beta }Z_{\kappa ,\beta }^{*}u_{\kappa \beta }\left(r\right)\right|}\right)\times \partial_{k}\left(\frac{\sum_{\kappa \gamma }Z_{\kappa ,\gamma }^{*}u_{\kappa \gamma }\left(r\right)}{\left|\sum_{\kappa \gamma }Z_{\kappa ,\gamma }^{*}u_{\kappa \gamma }\left(r\right)\right|}\right)\right] \end{array}$$

This is a quantized value associated with FE defects such as vortices or skyrmions that measure the number of times the displacement vector field (or related order parameter) wraps around a given point or region in space. These topological features can influence the electronic properties and are important for applications in topological quantum computing and advanced electronic devices.^[^ ^4^, ^6^ ^]^ In Equation (3) *A*_*i*_ is the surface of a small volume containing the polar structure, ϵ_*ijk*_ is the Levi‐Civita symbol, and $Z_{\kappa ,i\alpha }^{*}$ is the Born effective charge tensor component for atom κ, relating the *i*‐th component of the polarization to the α‐th component of the displacement of atom κ, obtained from DFT computations (see Experimental Section and Section S9, and Figures S8– S11) for the measured displacement field in Figure 3b. Displacement fields generated by FE vortices are characterized by a winding number $Q=\frac{1}{2\pi }\oint_{C}\nabla \theta \cdot dl=+1$ around the vortex core in 2D (Section S12, Supporting Information), regardless of their polarity (clockwise or anticlockwise).^[^ ^31^ ^]^ The angle $\theta =\arctan\left(\frac{\delta_{y}u_{002}}{\delta_{x}u_{002}}\right)$ is related to the components of the displacement vector **u_002_** (*r*). For polar BPs, the winding number captures the change in the displacement (polarization) direction in three dimensions. For anti‐polar Bloch points (ABPs), the winding number captures the reversal in the polarization direction. The integral remains the same but yields a negative winding number due to the opposite orientation. Merons and anti‐merons are characterized by half‐integer winding numbers $Q=\pm \frac{1}{4\pi }\oint_{C}\nabla \theta \cdot dl$ and a discontinuous polarization field.

By inspecting Figure 3, we identified topological polar structures such as vortices, merons, and BPs in the displacement field and electron density maps obtained by BCDI. Figure 3b shows that at time *t*_0_, two types of antiphase domains, α and β, can form in CBNO due to the presence of two bifurcation options. Combined with two possible in‐plane polarization directions^[^ ^23^, ^32^ ^]^ (“+” being parallel to the *a* and *b*‐axes and “−” being antiparallel to them), there are a total of four antiphase‐FE Z_2_ × Z_4_ domains (α +, β −, α −, and β +) existing in this system under ℓ = 0 (see Figure S18, Supporting Information). Unlike trimerization observed in other improper FE materials such as the hexamanganites and hexaferrites,^[^ ^33^ ^]^ where three‐fold symmetric domains are formed, the transition in CBNO leads to an in‐plane FE domain structure shown in Figure 3.

In Figure 3b,d, we qualitatively analyze the polarization vortex by inspection of the displacement field. Approximately 50 nm higher, in‐plane Bi‐ion displacement due to LG_ℓ_ electric field of ℓ = 0 induces a periodic strain modulation (Figure 3) in the *xy* −plane above the vortex and creates tensile strain due to its larger lattice constant. However, approximately 50 nm below, the material compensates for this by creating a predominantly compressive strain with a smaller lattice constant. By looking at multiple planes above and below along the *c* −direction, within the spatial resolution of the experiment, we corroborate this periodic modulation of tensile and compressive strain in the *xy* −plane. The strain maps also show intersecting domains that form fork‐like patterns in the *yz* −plane. These fork‐like features not only guarantee the formation of the vortices but also account for the observed polar BPs (Figure 3d,e).

The magnitude of the LG_ℓ_ electric field $E_{OAM}=10\;{\text{kVcm}}^{-1}$ for ℓ = 0 is smaller than the reported coercive field^[^ ^32^ ^]^ for CBNO (see Section S7, Table S1, and Figure S6, Supporting Information), thus explaining why we do not observe significant FE domain switching. To investigate how the observed strain modulation affects the FE polarization, we measured the relative shifts of α and β domains (Figures S18 and S19, Supporting Information) under the TL induced electric field. We notice that at time *t*_1_ under an OAM field of ℓ = 1, the α^+^ and β^+^ domains shift by vectors of 1/5[110] and −1/5[110], respectively, relative to the β^−^. This corresponds to polar displacements as a result of Bi^3 +^ shift in the range of 0.25–0.6 Å, which is consistent with what is found in bulk CBNO.^[^ ^23^ ^]^

The evolution of the complex polar textures shown in the planar views of the CBNO displacement field and magnitude (Figure 3) under the vortex electric field **E**_*OAM*_ can be better assessed by quantitative inspection of the three‐dimensional BCDI reconstructions. Besides the winding number Q, we can describe the topological structure in terms of a non‐zero toroidal moment,^[^ ^8^, ^9^, ^10^ ^]^ defined as $G=\frac{1}{2N}\sum_{i=1}^{N}r_{i}\times P_{i}$, where **P**_*i*_ is the local dipole moment located at **r**_*i*_ and *N* is the number of dipoles (cells). **P**_*i*_ is estimated using the Born effective charge tensor, $P_{i}=\sum_{\kappa \alpha }Z_{\kappa ,i\alpha }^{*}u_{\kappa \alpha }$.

**Figure 4:**
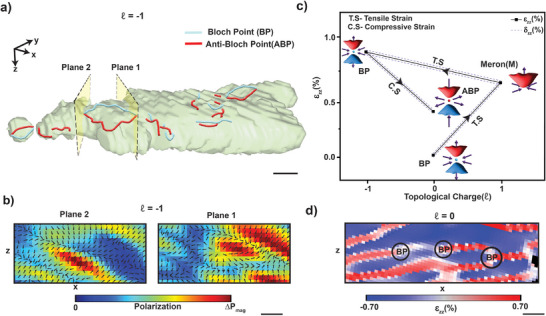
Twisted light‐induced polar Bloch point transitions and strain evolution. a) BCDI rendering of the magnitude of the toroidal moment density under OAM of ℓ = −1, capturing the topology formed by Bloch and anti‐Bloch points. b) 2D slices depicting the density of BPs and ABPs in the toroidal field, marked by planes in (a), with the normalized magnitude of polarization Δ*P*_*mag*_vectors overlaid. c) Hysteresis‐like loop capturing OAM field‐induced strain ϵ_*zz*_ and BP transition states in CBNO. d) BCDI strain map under an OAM field of ℓ = 0 showing that BPs are associated with regions of reduced polarization magnitude or significant strain gradients. Scale bars correspond to 100 nm.

We compute the FE toroidal moment from the reconstructed polarization. Regions of the large toroidal moment are plotted in **Figure** **4**a, where several ‘tubes’ and loops corresponding to the cores of vortices, antivortices, BPs, and ABPs are visible (see Figures S24– S28, Supporting Information). Unlike in incompressible fluids, where the divergence must vanish, a non‐zero divergence of the FE polarization due to complex topological structures like FE vortices and BPs signifies the presence of bound charges that play a role in the formation and stability of these structures under the TL‐induced vortex field. BPs and ABPs are identified by positive (red) and negative (blue) values within the reconstructed toroidal moment field. We observe a large number of 3D loops (Figure 4a) that resemble vortex rings under LG_ℓ_ electric field of ℓ = 1. We consider the case of one such loop, identified by plotting an isosurface corresponding to a maximum threshold of ±0.05*P*_*s*_ (Figure 4a). This loop is located in the vicinity of a single Bloch (anti‐Bloch) point, spanning a region of the CBNO crystal. This loop texture represents a departure from the coaxial string‐like topology observed under the TL‐induced vortex field of ℓ = 0 and ℓ = 1 (Figures S26– S28, Supporting Information). The 2D cut planes in Figure 4b show the vector field of change in *P*_*mag*_ (see Section S16, Supporting Information), allowing us to identify topological features such as head‐to‐head convergence, tail‐to‐tail divergence, BPs, ABPs, and merons.

In Ref. [15], polar BPs were observed in strained PbTiO_3_ films using phase‐field simulations and aberration‐corrected scanning transmission electron microscopy at the locations where a vortex core intersected a domain wall. Similarly, we find that the BP pair is located at the intersection of the vortex‐antivortex loop with a domain wall separating regions of opposite strain magnitude (Figure 4d). It was predicted in the same reference that polar BPs can be stabilized using substrate‐induced tensile strain in FE films. In Ref. [31], a periodic shear strain landscape was generated by stacking freestanding FE perovskite layers with controlled twist angles, akin to Moiré lattices.^[^ ^34^ ^]^ An important remark is that in both cases above, the inter‐layer strain in the film cannot be dynamically tuned.

Here, in Figure 4c, we observe that under LG_ℓ_ electric field transitioning from ℓ = 0 to ℓ = 1, then ℓ = −1, and back to ℓ = 0, we can manipulate the global and local strain within the CBNO crystal in a controllable manner (Figures S19 and S21, Supporting Information). Under a global tensile strain ε_*zz*_ of 0.7%, there is a transition from BPs to merons. A slight increase in the tensile strain to ε_*zz*_ of 0.75%, by changing the vortex field to ℓ = −1, reverts to a global Bloch point state. Subsequent compressive strain back to ℓ = 0 transitions to a global ABP state within the CBNO crystal (Tables S2 and S3 and Figure S23, Supporting Information). Figure S20 (Supporting Information) shows the local mapping of the strain modulations and induced shear strains that mediate the observed BP transitions. It is worth noting that such controlled strain manipulation and the observed strain landscape are unique, as they cannot be attained, to the best of our knowledge, either by epitaxial strain or by any pattern of externally applied stresses or electrodes.

In Figures 3 and 4, we have presented experimental evidence of the formation of polar topological states in the quasi‐2D FE nanoflake, with emerging polarization vortex structures that are axially symmetric at time *t*_0_ under LG_ℓ_ electric field of ℓ = 0. However, the creation of a vortex costs additional energy due to the existence of the vortex core where the FE is suppressed. Thus, under UV LG_ℓ_ beam illumination of ℓ = 1, the singularity at the vortex core can be completely eliminated by the escape of the vector field into the third dimension (see Figure S20, Supporting Information) along the vortex axis, leading to the emergence of chiral meron‐like structures in the flake. Under TL illumination of ℓ = −1, the system returns to a BP state, while subsequent compressive strain allows the polar structure to transition to an ABP state under a TL‐induced LG_ℓ_ field of ℓ = 1.

In addition to the observed TL‐induced OAM field‐induced strain and manipulation of real‐space FE polar structures from BCDI, OAM‐dependent Raman measurements on CBNO suggest a possible correlation with twisted UV light‐induced excitation of the zone‐center and zone‐boundary modes in CBNO (see details in Experimental Section and Figures S9, S10 and S12, Supporting Information).

**Figure 5:**
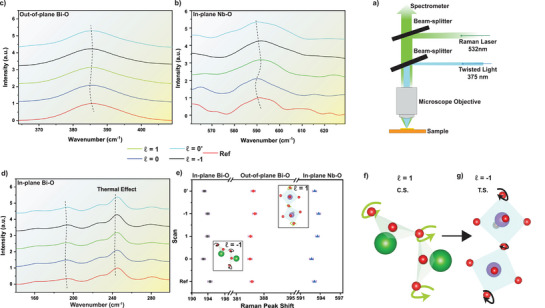
Twisted‐light dependent Raman spectroscopy on CBNO: a) Schematic of the in situ Raman experimental setup designed to probe photo‐induced structural changes, modulation of phonon modes, and domain dynamics in a CBNO nanoflake under LG_ℓ_ light illumination. The setup utilizes a 532 nm Raman laser with a 375 nm coherent twisted light of varying topological charges. b) Line plots showing the behavior of the out‐of‐plane phonon mode for the Bi‐O layer. The spectra demonstrate reversible shifts in peak positions, characterized by a tensile shift from the reference to ℓ = 0, compression under ℓ = 1, and a reversion to a tensile state under ℓ = −1, with minimal changes upon returning to ℓ = 0. This suggests significant OAM‐induced modulation of vibrational states. c) Line plots illustrating the out‐of‐plane phonon modes of the Bi‐O layer, including isolated Bi atom responses. The plots indicate compression effects attributed to thermal influences from continuous illumination, highlighting the sensitivity of Bi phonon modes to UV LG_ℓ_ light and thermal input. d) Line plots for the in‐plane phonon modes of the Bi‐O layers, showing reversible Raman peak shifts similar to those observed in the out‐of‐plane Bi‐O modes, underscoring the symmetric impact of OAM on in‐plane Bi‐O vibrations. e) Summary of Raman peak shifts across different modes when interacting with twisted light carrying various topological charges. Insets depict the structural and bond modifications along with torsional strain effects induced by twisted light, demonstrating the impact of OAM on lattice dynamics (refer to Figures S10, S13, S32, and S33 and Table S4, Supporting Information for detailed analysis). f–g) Schematic representations of interactions between twisted light with topological charges ℓ = 1 (Compressive Strain) and ℓ = −1 (Tensile Strain), with Bi‐O tetrahedra and Nb‐O octahedra, respectively. These illustrate the torsional and structural changes imparted by non‐zero OAM, contributing to the understanding of vortex‐light‐induced Raman activity.

**Figure** **5**a illustrates the experimental setup for OAM‐dependent Raman measurements on CBNO. Details of the experimental methodology can be found in Experimental Section. A reference scan was initially performed to confirm the unperturbed crystal structure of CBNO, as shown in Figure S30 (Supporting Information). Subsequent Raman scans were conducted under various OAM conditions, similar to BCDI measurements (ℓ = 0, ℓ = 1, ℓ = −1, and returning to ℓ = 0). We monitored both the in‐plane zone‐center phonon mode, reported around 190 cm^−1^ for the Bi‐O bond,^[^ ^35^ ^]^ and the out‐of‐plane phonon mode, as depicted in Figures 5d,c, respectively. Illumination with ℓ = 0 resulted in tensile strain along the c‐axis, while the OAM field with ℓ = 1 induced a compressive effect. These findings are consistent with the BCDI measurements. A reversible behavior was observed with ℓ = −1, and returning to ℓ = 0 resulted in a Raman spectral shift that matched the initial state. This reversibility was also seen in the zone‐boundary phonon mode, which has been reported around 580 cm^−1^ for the Nb‐O in‐plane Raman peak,^[^ ^36^ ^]^ as shown in Figure 5b. The consistent nature of these trends was confirmed by tracking the Raman peak position at full width at half maximum (FWHM), as illustrated in Figure 5e.

Schematics showing the effects of twisted light (TL)‐induced OAM fields (ℓ = 1 and ℓ = −1) on the Bi‐O and Nb‐O bonds are presented in Figure 5f,g, respectively. The schematic is inspired by the data shown in Figures 5b–d, where we observe a reversible tensile and compressive behavior in strain in the phonon modes. This reiterates and emphasizes the ability of the TL‐induced electric fields to control the bending and breathing of the phonon modes in the nanocrystal. An inset illustrating the inverse case is shown in Figure 5e, and a detailed analysis of the shifts in polarization is depicted in Figure S9 (Supporting Information). The Raman spectroscopy results align with the proposed interaction mechanism between TL and FE polarization in CBNO. Qualitatively, comparisons between BCDI and Raman spectroscopy reveal a hysteresis‐like behavior in both strain and Raman peak shifts. This is analogous to the traditional hysteresis observed between polarization and electric field in FEs, where the beam topological charge acts similarly to a variable electric field. However, the complex nature of the LG_ℓ_ electric field provide a wider space to manipulate the sample. This demonstrates the potential for controlling FE topologies using TL, opening new avenues for research into vortex‐light–matter interactions.

We propose that the interaction mechanism involves the coupling of TL with the electronic structure, leading to shifts in phonon modes within the CBNO nanoflake. One potential interaction mechanism is intra‐band transitions, as discussed in Sections S8 and S10 (Supporting Information). We suggest that electron‐mediated shifts in the phonon mode structure result in the observed changes in strain, where a preferential shift in the electronic structure is noted (see Figure S14, Supporting Information) within the tight‐binding approximation of the CBNO band structure. In both BCDI and Raman spectroscopy experimental techniques, the effects are observed post electron–phonon renormalization, leading to the observed Raman peak shifts, FE polarization, and strain behavior. A thermal effect is shown in Figure 5d. The wavenumber relates to Raman frequency shifts in the Cs‐O bonds. However, Cs does not contribute to the ferroelectric landscape of CBNO. This thermal effect also helps us isolate the effect of a thermal energy induced shifts and the shifts induced by the orbital angular momentum of the TL on the ferroelectric behavior of the system.

Our experimental results, supported by theoretical analysis, reveal that the OAM states ℓ = 0 and ℓ = 0′ correspond to a uniform electric field distribution, while ℓ = 1 and ℓ = −1 result in a non‐uniform electric field when illuminating the nanoflake. In both cases, the strength and direction of the electric field vary depending on the beam waist and the topological charge. Under a homogeneous electric field corresponding to ℓ = 0, a stable 2D vortex structure was observed at time *t* = 0, with the vortex core aligned along one of the crystallographic axes [001] (see Figure 3c). A small quasi‐static field applied at *t* = 0 stabilized an axial vortex core, which is topologically protected by two antiphase nanodomains with nearly homogeneous spontaneous polarization (see Section S8 and Figure S22, Supporting Information).

Similar to ferromagnetic vortices, the core of the observed ferroelectric vortex behaves like a dipole, offering the potential to rotate the vortex axis under the influence of a small quasi‐static electric field, whether homogeneous or inhomogeneous. Due to strong ferroelectric anisotropy, which hinders the rotation of the dipolar vortex core and tends to align it along one of the crystallographic directions, the orientation of the core in the field is determined by the minimization of dipolar and anisotropy energies. As the vortex core is coupled to the antiphase domains, the plane of the vortex, which is perpendicular to the core axis, can rotate along with the core. The core size is sensitive to variations in the TL‐induced electric field, and as these variations occur, the nanoflake gradually transitions toward a single‐domain state, as observed at time *t* = *t*_1_.

Manipulating FE polarization in quasi‐2D materials like CBNO using incident vortex fields generated by coherent twisted ultraviolet light offers distinct advantages over using pulsed terahertz infrared photons, as traditionally employed in 3D perovskite materials like SrTiO_3_. Twisted ultraviolet light carries topological charge, imparting angular momentum to the electrons and ions in the material, leading to precise interactions with ferroelectric polarization. This helical phase can stabilize transitions between Bloch points and merons by aligning polarization vectors and modifying the energy landscape, thereby lowering the energy barriers for these transitions. The higher spatial precision and photon energy of UV light allow for more effective and localized polarization changes compared to terahertz infrared pulses. Additionally, vortex fields carrying orbital angular momentum can selectively interact with specific phonon modes or electronic states that are not easily accessible with terahertz infrared pulses, providing a unique tool for manipulating ferroelectric polarization.

In 3D perovskite oxides systems,^[^ ^37^, ^38^, ^39^ ^]^ pulsed terahertz infrared photons can resonantly excite specific phonon modes, inducing transient changes in ferroelectric polarization and numerous emergent phenomena.^[^ ^40^, ^41^, ^42^ ^]^ However, the additional degree of freedom and increased pathways for energy dissipation in 3D systems make it more challenging to achieve the same level of control and precision as in quasi‐2D systems. Therefore, using electric field of coherent twisted ultraviolet light to manipulate ferroelectric polarization in quasi‐2D CBNO provides superior control and effectiveness, enabling the stabilization of complex topological transitions.

### 2.1 Observed Strain Hysteresis

Figures 1d and 4c provide clear experimental evidence of strain hysteresis in CBNO under twisted light illumination. This hysteresis is a hallmark of nonlinear, history‐dependent ferroelastic domain dynamics induced by the orbital angular momentum (OAM) of the light field.

The observed strain hysteresis suggests discrete, irreversible domain switching events in response to varying OAM topological charge ℓ. As in conventional ferroelectric polarization‐field hysteresis, this behavior implies the nucleation, reorientation, and stabilization of non‐trivial domain configurations (e.g., Bloch points, vortex‐antivortex pairs) which do not fully relax when ℓ is reduced. Additionally, domain wall pinning by intrinsic defects or impurities likely enhances this lagged response, contributing to the observed memory effect.

The coupling between TL and FE polarization creates a complex energy landscape with multiple metastable states separated by energy barriers. This energy profile results in different structural pathways during the increasing and decreasing ℓ cycles, producing the characteristic hysteresis loop. The behavior is consistent with first‐order‐like phase transition dynamics, where switching between distinct strain or polarization states is discontinuous and strongly path‐dependent.

The spatial phase profile of TL, characterized by the topological charge ℓ, introduces an inhomogeneous driving force on the lattice. This optical torque modulates the strain state in a non‐single‐valued manner, leading to hysteresis in the strain response. Furthermore, local lattice distortions generated by photoinduced effects persist beyond the duration of the applied OAM, suggesting slow relaxation dynamics or trapping in metastable states.

This observed strain hysteresis – with its strong dependence on the history of applied ℓ – demonstrates the potential for OAM light to induce non‐volatile strain states in ferroelectric materials. Such memory effects are attractive for reconfigurable photonic, straintronic, and non‐volatile memory applications. Future pump‐probe BCDI or ultrafast Raman studies could provide time‐resolved insights into the domain switching and relaxation processes, while theoretical modeling using Landau‐Ginzburg approaches incorporating OAM coupling terms may capture the underlying energy landscape and switching dynamics.

### 2.2 Alienating Thermal Effects

The strain and polarization changes induced by TL are qualitatively distinct from conventional thermal effects. Temperature‐dependent Raman measurements on the Nb–O vibrational modes display a monotonic, linear shift with increasing temperature, consistent with thermal expansion or phonon softening(see Figure S35, Supporting Information). In contrast, under TL illumination, the Raman modes exhibit cyclical, hysteresis‐like behavior as the topological charge ℓ is varied between −1, 0, and +1 – a signature of complex, history‐dependent domain reconfiguration rather than continuous heating effects.

Complementary BCDI measurements further corroborate this interpretation, revealing spatially localized lattice distortions and domain rearrangements that correlate with the applied OAM of the vortex beam. Given the moderate laser power density and the limited penetration depth of 375~nm light in CBNO, significant bulk heating is unlikely. Overall, the combined Raman and BCDI evidence confirms that the observed strain hysteresis originates from intrinsic OAM‐induced ferroelastic phenomena rather than trivial laser‐induced thermal effects.

### 2.3 Mechanism of Twisted Light‐Induced Strain

At this stage, it is not quite obvious from the exact microscopic mechanism responsible for twisted UV light induced strains. We shall address a few possibilities.

#### 2.3.1 Non‐Linear Optical Processes

In our investigations, the microscopic mechanisms responsible for TL‐induced strain heterogeneity in CBNO could be potentially attributed to the interplay between non‐linear optical processes and spatial phase modulation of the incident vortex beam. Although the UV excitation (375 nm, ≈3.31 eV) is slightly below the measured bandgap of CBNO (3.64 eV), the wavefront‐shaping, and structured electromagnetic fields provided by the TL facilitate multi‐photon absorption. This multi‐photon process may lead to the generation of photoexcited carriers that locally perturb the crystal lattice, giving rise to heterogeneous strain fields observed by BCDI (see Figures 3 and 4). Also, experimental observations–such as intensity/power‐dependentsee (see Section S19 and Figure S37, Supporting Information) non‐linear shifts in Raman modes (notably the Nb–O vibrational mode) and spatially resolved lattice distortions via BCDI–lend support to the contention that non‐linear absorption processes^[^ ^18^, ^19^ ^]^ are pivotal in realizing these strain heterogeneities.

Moreover, the formation of head‐to‐head and tail‐to‐tail domain walls, as well as BPs, suggests that charge screening plays a critical role in the overall mechanism. In the non‐linear screening regime, the photoexcited electron–hole pairs effectively screen the spontaneous polarization through mechanisms where the screening energy is intrinsically proportional to the electronic bandgap. Such interplay between charge screening and strain has parallels with the fast photostrictive responses observed in ferroelectric materials, where the rapid, local strain induced by light is notably non‐thermal in nature.^[^ ^43^ ^]^

Moreover, the formation of head‐to‐head and tail‐to‐tail domain walls observed in our BCDI results, as well as BPs, suggests that charge screening plays a critical role^[^ ^44^ ^]^ In the non‐linear screening regime, the photoexcited electron–hole pairs effectively screen the spontaneous polarization through mechanisms in which the screening energy is proportional to the electronic bandgap. Concurrently, the unique spatial phase and orbital angular momentum associated with twisted light can potentially introduce a chiral component to the excitation process. This aspect can further modulates the local electronic structure and reinforces strain heterogeneity via selection rules that permit non‐dipolar transitions.^[^ ^45^ ^]^ This scenario is further substantiated by studies demonstrating that the OAM inherent in twisted light facilitates interaction pathways leading to hyper‐Raman and hyper‐Rayleigh responses, underscoring the role of non‐linear optical susceptibility in complex materials.^[^ ^45^, ^46^ ^]^

In combination, these effects provide a robust framework for understanding how multi‐photon absorption, modulated by the vortex beam's spatial structure, induces complex domain dynamics and strain patterns in CBNO.

#### 2.3.2 Rashba–Dresselhaus Effects

In addition to the non‐linear excitation pathways, the formation of domain walls with accompanying BPs/ABPs indicates that charge screening plays a critical role. The screening of spontaneous polarization in ferroelectrics such as CBNO is achieved when generated electron–hole pairs provide sufficient compensation for the intrinsic polarization field–a process that is energetically linked to the electronic bandgap.^[^ ^47^ ^]^

Recent theoretical studies^[^ ^20^, ^21^ ^]^ on Rashba–Dresselhaus FE CBNO have highlighted the intimate coupling between its FE polarization and strong spin–orbit interactions and strain.^[^ ^22^ ^]^ In the FE state of CBNO, the absence of inversion symmetry leads to pronounced Rashba spin splitting, which is highly sensitive to strain. Mechanical strain alters local lattice parameters and modulates the ferroelectric polarization, thereby changing the internal electric fields that govern the Rashba effect.

Both tensile and compressive strains (see Figure 4) doesn't only manipulate the texture of FE polarization but can tune the band structure of CBNO, affecting not only the bandgap but also the magnitude of the spin splitting. This strain‐induced modulation provides a powerful avenue for engineering persistent spin helix states,^[^ ^48^ ^]^ which are critical for low‐power spintronic applications. Moreover, the interplay between strain, complex domain configurations (such as head‐to‐head and tail‐to‐tail domain walls and BPs), and TL excitation leads to non‐linear screening effects that manifest through the generation of electron–hole pairs to fully screen the spontaneous polarization. Since the screening energy is proportional to the electronic bandgap, these findings underscore the potential of CBNO as a platform for dynamically tuning both its electronic and spintronic properties.

Overall, we suggest that the interplay between non‐linear optical processes–including multi‐photon absorption–and the spatially varying phase profile of the vortex beam can be central to the observed phenomena. The electromagnetic fields of the twisted light induce non‐linear polarization effects in the CBNO crystal, can enhance the generation of photoexcited carriers. These carriers modify the local lattice screening and electronic distribution, leading to heterogeneous strain fields that are further reinforced by the spatial modulation of the vortex beam. Although direct, microscopic validation of each individual process remains challenging, the combined evidence from Raman spectroscopy, BCDI, and intensity studies provides plausible support for these non‐linear effects as possible drivers of the strain heterogeneity and domain dynamics observed in our experiments.

### 2.4 Selective Coupling to Polar Phonon Modes Via Sub‐Bandgap Excitation in CBNO:

In CBNO, the 375 nm UV excitation (approximately 3.31 eV) is slightly below the measured bandgap of 3.64 eV (see Figure S12, Supporting Information). Under these sub‐bandgap conditions, photons can selectively excite shallow defect states that reside within the bandgap. These defect states, when populated, interact with the lattice via electron–phonon coupling, particularly influencing the polar phonon modes that are highly sensitive to local charge distributions. The excitation of these shallow defect states leads to localized modulation of the polar phonon modes, resulting in spatially inhomogeneous lattice distortions. Under the influence of twisted light, with its unique spatial phase and high intensity, these localized effects are further amplified, ultimately giving rise to the observed strain heterogeneities in CBNO.

This mechanism involves an initial sub‐bandgap excitation of defect states, which then couple selectively to polar phonon modes, modifying the local ferroelectric polarization and creating regions of differential strain. Future studies could confirm this mechanism by employing time‐resolved Raman spectroscopy and pump–probe techniques to monitor the dynamics of defect state excitation and their influence on polar phonon modes. Moreover, theoretical models that incorporate electron–phonon coupling under twisted‐light excitation, alongside spatially resolved measurements such as scanning probe microscopy under in situ illumination, would provide further insights into the selective modulation of polar modes and the resulting strain distributions.

To further elucidate the exact microscopic mechanisms at play in CBNO under TL excitation, several experiments can be envisaged. Time‐resolved TL pump–probe measurements, such as ultrafast BCDI and time‐resolved Raman spectroscopy, would allow us to capture the dynamics of photoexcited carrier generation, lattice distortion, and domain wall motion on ultrafast timescales. Systematic intensity‐dependent studies, where the incident light intensity and topological charge are varied, could help distinguish threshold behavior associated with multi‐photon absorption from linear optical responses. Complementary temperature‐dependent experiments would further aid in decoupling thermal effects from intrinsic non‐linear optical phenomena. Additionally, non‐linear optical techniques like twisted‐light second‐harmonic generation (SHG) could provide direct insights into the evolution of the polarization state, while time‐resolved angle‐resolved photoemission spectroscopy (TR‐ARPES) might reveal modifications in the band structure and Rashba spin splitting induced by twisted light. Finally, spatially resolved measurements using scanning probe techniques such as piezoresponse force microscopy (PFM) under in‐situ illumination could map ferroelectric domain dynamics and clarify the role of head‐to‐head and tail‐to‐tail domain walls, as well as Bloch points, in charge screening. Collectively, these experimental approaches would offer a comprehensive understanding of the interplay between non‐linear absorption, charge screening, and domain dynamics in CBNO.

## 3 Conclusion and Outlook

We have demonstrated that the orbital angular momentum electric field from twisted light can dynamically induce strains (both tensile and compressive) up to ≈0.8% along the c‐axis in individual quasi‐2D ferroelectric CBNO crystals. These strains are quantifiable, with nanometer resolution, and can be controlled reversibly, creating significant strain gradients. However, we observed irreversibility in some strain states, particularly during cycling of the topological charge (ℓ) of twisted light. This irreversibility resembles hysteresis‐like behavior, which could arise from defect pinning, localized energy barriers, or strain‐induced polarization switching. A deeper investigation into this hysteresis effect is required, including systematic hysteresis loop measurements to fully characterize the coupling mechanism. This phenomenon is potentially universal to quasi‐2D ferroelectric systems with weak out‐of‐plane bonding along the stacking direction. For example, in CBNO, the reported piezoelectric coefficient *d*_33_ (8 pC N^−1^)^[^ ^49^ ^]^ is significantly smaller than in typical 3D perovskite oxides (e.g., PbTiO_3_, BaTiO_3_,^[^ ^50^ ^]^ BiFeO_3_). Our observations show that topologically protected polar structures and transitions from Bloch points to merons occur under a light‐carrying orbital angular momentum electric field, mediated by induced tensile strains. These findings align with earlier reports of polar Bloch points in strained epitaxial thin films. However, in epitaxial thin films, the mechanical boundary conditions are rigid and determined by the atom‐on‐atom replication of the substrate structure, leaving little room for modification. Additionally, reversible and dynamic control of torsional, shear, or inhomogeneous strain patterns is not easily achievable via epitaxy.

In summary, we have found that it is possible to induce non‐trivial ferroelectric textures and polar structures, as well as strains in quasi‐2D freestanding ferroelectric crystalline flakes. The coupling mechanism involves the twisted light's spatially varying electric field interacting with the material's polarization and strain fields, leading to localized ionic displacements and quasi‐static strain gradients. These couplings not only drive the nucleation of vortex‐like and Bloch‐type modulations but also create persistent, field‐driven strain profiles that may depend on the specific topological charge (ℓ) of the light. This interplay between the electric field gradients, defects, and polarization states underpins the hysteresis‐like effects observed in our experiments. Furthermore, the nature of these 2D and 3D topological structures can be largely tuned by controlling the twisted light's topological charge.

Moreover, the interaction of twisted light with ferroelectrics potentially opens new avenues for research into the dynamical twist‐multiferroics^[^ ^42^ ^]^ and the braiding of polar 1D structures. To further explore these phenomena, a full hysteresis loop measurement and time‐resolved techniques are necessary to resolve the dynamic behavior of polarization and strain under varying ℓ. Additionally, combining twisted UV light with other modalities, such as higher‐order Bessel beams or circularly polarized twisted light, could uncover new mechanisms of light‐matter interaction. Upgrades in the coherence^[^ ^51^, ^52^ ^]^ of synchrotrons and the development of time‐resolved orbital angular momentum pump orbital angular momentum‐probe Bragg coherent diffractive imaging techniques that require twisted X‐rays^[^ ^53^ ^]^ could potentially provide deeper insights into the dynamical control of novel states of matter that are unattainable by conventional thermodynamic methods. Additionally, the development of new Bragg coherent diffractive imaging methods to access in‐plane strain and orbital angular momentum‐dependent Raman spectroscopy to capture chiral or more nuanced twisted‐light interactions could represent a paradigm shift in engineering beyond 2D. These advances would not only enhance the understanding of twisted‐light interactions but also open possibilities for designing multifunctional devices, where ferroelectric and multiferroic states can be dynamically controlled in real‐time. This includes the potential for dynamical manipulation of the twist angle in which other modes of twisted light, such as higher‐order Bessel beams with higher penetration, could play important roles. Shedding further light on these advances would be a great challenge, for both theory and experiment, but is beyond the scope of the present work.

In the near future, combining twisted light with experimental techniques such as angle‐resolved photoemission spectroscopy (ARPES) and high‐harmonic generation (HHG) will serve as promising tools for probing these reported interactions. ARPES can be used to monitor changes in the spin texture, while HHG can generate attosecond pulses carrying OAM for ultrafast manipulation of the spin states. This dual capability of controlling both real‐space polarization and momentum‐space spin texture represents a significant advancement in the field of spintronics, with profound implications for next‐generation memory devices, quantum computing, and other applications requiring precise spin current control.

## 4 Experimental Section

### Fabrication of Freestanding Quazi‐2D Flakes

Free‐standing CBNO 2D flakes were synthesized using a molten‐salt assisted method.^[^ ^23^, ^36^ ^]^ Further, pre‐characterization was carried out to confirm the stoichiometry using XPS (Section S4 and Figure S2a, Supporting Information), morphology using XRD and SEM (Sections S2 and S3 and Figure S1, Supporting Information) and bandgap using transmission spectroscopy (Section S5 and Figure S2b, Supporting Information). Stability measurements were also performed under excitation with UV light with gaussian profile and timed raman measurements were taken for 120 mins as shown in Figures S34 and S38 (Supporting Information).

### Twisted Light Bragg Coherent Diffractive Imaging Experiments

The Si (111) monochromator at the Advanced Photon Source's sector 34‐ID‐C was utilized to isolate coherent X‐ray photons with an energy of 9.0 keV. The beam's monochromaticity was defined by an energy bandwidth of 1 eV, and it exhibited a 0.7 µm transverse coherence length. The X‐ray beam was focused onto the sample using a pair of Kirkpatrick‐Baez mirrors situated after the beam‐defining aperture. For this experiment, the beam size was set to 700 by 700 nm. The principle of the Bragg coherent diffraction experiment was illustrated in Figure S3 (Supporting Information). A Medipix2 CMOS X‐ray detector was positioned around the diffraction sphere using a motorized arm. The detector was aligned with the outgoing (002) characteristic Bragg reflection from the CBNO sample. To magnify the interference fringes in the diffraction pattern, the detector was placed 1.2 m away from the sample. An evacuated flight tube positioned in the path from the sample to the detector helped minimize photon scattering losses in the air. For such a photon‐starving technique as nanoscale Bragg coherent diffractive imaging, the use of an evacuated flight tube, high sensor gain, and the detector's photon counting mode were essential. An Ultra‐Violet laser of 375nm, guided via a confocal system available at the 34‐IDC beamline, was focused down to 10 µm at a normal incidence to the isolated CBNO flake of interest. During the collection of rocking curves, the sample was continuously illuminated. A spatial light modulator (SLM) was employed to select the optical beam's topological charge, thereby controlling the OAM and torque transferred to the crystal (see Section S6 and Figure S3, Supporting Information). Rocking curves were collected as a series of 2D diffraction patterns near the CBNO 002 Bragg peak, corresponding to 2θ = 13.26°, with a scanning range of Δθ = ±1.4° in the vicinity of the Bragg peak. A total of 360 patterns were gathered in a single rocking curve. The dataset for the virgin state (topological charge, ℓ = 0) was obtained before cycling the CBNO crystal. The subsequent states under continuous LG_ℓ_ beam illumination, ℓ = 1, ℓ = −1 and ℓ = 0 were recorded after 30 cycles of ℓ = 0 illumination and release. This takes about 30 mins for one single 3D scan to be collected. Thus for all OAM applications *T* = *t*_0_ = *t*_1_ = *t*_2_ = *t*_3_ = 30 mins. This ensured that the system was entirely in equilibrium under laser illumination.

### Phase Retrieval Process

The collected 3D diffraction patterns were inverted from reciprocal into real space using iterative phase retrieval algorithms.^[^ ^50^ ^]^ This gives us the complex wavefield post diffraction, which was given by ρ(**r**) = ρ_0_(**r**)*e* ^(*i*ϕ(**r**))^ where ϕ(**r**) is the phase and ρ(*r*) is the amplitude of the wave. The phase carries the information of about the displacement field *u*_002_, which could be determined by using ϕ(**r**) = **G_002_**.**u_002_** (**r**). The shape and size of the nanocrystal were estimated from the reconstructed Bragg Electronic Density(ρ(**r**)). For a given applied TL topological charge, real‐space images of the CBNO nanocrystal were reconstructed with approximately 33 nm spatial resolution as determined by the phase retrieval transfer function (PRTF) (see Figure S28, Supporting Information). The 3D nature of the reconstructed nanocrystal allowed to slice through the particle and analyze the different FE topologies. Fienup's hybrid input‐output (HIO) approach was the basis for iterative phase retrieval techniques, which were further enhanced by randomized overrelaxation. When the measurement locations were close enough to one another to satisfy the oversampling criterion, an important stage in the process was to reverse the diffraction data using a computer algorithm. The first stage was to assume a 3D support volume where the sample density would be completely limited. These techniques impose a Fourier transform, both forward and backward, between the real and reciprocal spaces, imposing an intensity mask constraint in the former and a support constraint in the latter.

### Twisted‐Light Raman Spectroscopy

A 532 nm wavelength light from the Witec Alpha 300R series was used to perform the Raman Measurements. The Witec Alpha 300R was modified with a free beam coupler to introduce the twisted light of wavelength 375 nm on to the sample surface. During the collection of a single spectrum, the sample was continuously illuminated. A spatial light modulator (SLM) was employed to select the optical beam's topological charge, thereby controlling the OAM and torque transferred to the crystal (see Section S19 and Figure S30, Supporting Information). An Andor CCD was used to collect and count photons scattered from the CBNO nanoflake. A spectroscope grating of 1800 grmm^−1^ was used to obtain a high‐resolution spectrum of the sample under various topological charges. A Zeiss 100x microscope objective was used for focusing the laser beams onto the nanoflake as shown in Figure S30 (Supporting Information). The integration time of 1 *s* was used to collect 100 accumulations of the spectrum to obtain a clean spectrum with a low signal‐to‐noise ratio. The reference spectrum was collected before cycling the system. The subsequent spectrums were collected under continuous illuminations of ℓ = 1, ℓ = −1 and ℓ = 0 were collected. The experimental procedure is highlighted in Figure S36 (Supporting Information).

### First‐Principles Calculations

First‐principles calculations were performed using the CASTEP (Cambridge Serial Total Energy Package)^[^ ^54^ ^]^ and Quantum ESPRESSO (QE)^[^ ^55^ ^]^ packages. The calculations involving the application of an external light beam carrying Orbital Angular Momentum (OAM) with different topological charges (ℓ) were performed using CASTEP. The experimental electric field component of the OAM beam was explicitly included in these calculations. The Perdew‐Burke‐Ernzerhof (PBEsol) exchange‐correlation functional within the generalized gradient approximation (GGA)^[^ ^56^ ^]^ was employed for all calculations. Norm‐conserving pseudopotentials and a plane‐wave basis set with a kinetic energy cutoff of 600 eV were used. The pseudopotentials for Cs, Bi, Nb, and O were taken from the CASTEP library.

The Brillouin zone was sampled using a 6 × 6 × 6 Monkhorst‐Pack grid.^[^ ^57^ ^]^ The self‐consistent field (SCF) calculations were converged to an energy threshold of 1 × 10^−6^ eV. The initial molecular structure of CBNO was obtained from experimental theoretical data predictions available on the Materials Project. The structures were optimized using density functional theory (DFT) as implemented in Quantum ESPRESSO. The Perdew‐Burke‐Ernzerhof (PBEsol) exchange‐correlation function was chosen to describe the electron‐electron interactions. The unit cell parameters are: *a* = 11.87380 Å, *b* = 5.45730 Å, *c* = 5.55750 Å, with angles α = 90°, β = 90°, γ = 90°. Atomic positions were fully relaxed until the forces on each atom were less than 0.01 eVÅ^−1^, and the stress tensor was below 1× 10^−3^ GPa. The polarizability was computed using density functional perturbation theory (DFPT).^[^ ^58^ ^]^ Infrared and Raman spectra were obtained by calculating the dynamical charges and the Raman tensors, respectively. The dielectric function and the Raman activities were computed to simulate the IR and Raman spectra.

## Conflict of Interest

The authors declare no conflict of interest.

## Author Contributions

R.H., W.C., and E.F. designed and performed the BCDI experiment. J.J. and J.S. grew and provided the samples. V.T, P.B., and M.N synthesized the twisted beam.M.N and V.T acknowledged the support of The Office of Advanced Scientific Computing Research within the Office of Science US Department of Energy (DOE) under grant No DE‐SC0024676. All authors interpreted the results and contributed to writing the manuscript.

## Supporting information

Supporting Information

## Data Availability

The data that support the findings of this study are available on request from the corresponding author. The data are not publicly available due to privacy or ethical restrictions.
