# Supplementary material for: Manipulating Ferroelectric Topological Polar Structures with Twisted Light
Source: Adv Mater. 2025 Jun 6;37(33):2415231. doi: 10.1002/adma.202415231 (PMC12369684; doi:10.1002/adma.202415231)
Supplement: Supplementary file 1 — Supporting Information [file ADMA-37-2415231-s001.docx]

Manipulating ferroelectric topological polar structures with twisted light

Nimish P. Nazirkar, Viet Tran, Pascal Bassène, Atoumane Ndiaye, Julie Barringer, Jie Jiang, Wonsuk Cha, Ross Harder, Jian Shi, Moussa N’Gom, Edwin Fohtung.

Nimish P. Nazirkar, Dr. Atoumane Ndiaye, Dr. Julie Barringer, Dr. Jie Jiang, Prof. Jian Shi, Prof. Edwin Fo- htung

Department of Materials Science and Engineering, Rensselaer Polytechnic Institute (RPI), Troy, NY 12180, USA

Email Address: [ngomm@rpi.edu](mailto:ngomm@rpi.edu) and [fohtue@rpi.edu](mailto:fohtue@rpi.edu) Viet Tran, Dr. Pascal Bassène, Prof. Moussa N’Gom

Department of Physics, Applied Physics, and Astronomy, RPI, Troy, NY 12180, USA

Shirley Ann Jackson, Ph.D. Center for Biotechnology and Interdisciplinary Studies, Rensselaer Polytechnic In- stitute, Troy, New York 12180, USA.

Dr. Wonsuk Cha, Dr. Ross Harder

Advanced Photon Source, Argonne National Laboratory, Lemont, Illinois 60439, USA

**Supplementary Materials:**

## Section S1: Sample Preparation

Free-standing CBNO 2D flakes were synthesized using a molten-salt assisted method^[[1]](#endnote-1)^. Powder precursors of Cs_2_CO_3_ (Sigma-Aldrich, 99%), Bi_2_O_3_ (Sigma-Aldrich, 99.9%) and Nb_2_O_5_ (Sigma-Aldrich, 99.9%) were

finely ground in a molar ratio of 3:2:4. This mixture was then combined with CsCl salt (Sigma-Aldrich, 99.9%), which was ten times the weight of the other precursors and loaded into an alumina crucible. The crucible was then heated in a horizontal furnace in open air, with the heating process divided into three stages. First, the tem- perature was increased to 900^◦^*C* at a ramp rate of 225^◦^*C/*hr. The crucible was then held at this temperature for

an additional 4 hours. Lastly, the furnace was allowed to cool down slowly at a rate of 2^◦^*C/*min. Following this

process, CBNO square crystals with dimensions on the sub-micron to millimeter scale and varried thickness

were formed within the crucible. These crystals were cleaned using deionized water. The exfoliation of the CBNO single crystal was accomplished through the use of the scotch tape method. Thin crystalline flakes of CBNO

were then transferred onto a transmission electron microscopy (TEM) grid or a silicon substrate to facilitate sub- sequent operando Raman and BCDI measurements.

## Section S2: SEM Analysis

To perform BCDI experiment, one of the important prerequisites include isolated nanoflakes or nanocrystals on a Si substrate as mentioned in section S1. To confirm this, we look at our samples under an scanning elec-

tron microscope as shown in Figure S1 (a). We can clearly see some isolated nanoflakes around huge flakes with chipped edges and irregular geometry of about 1 um in spatial dimensions.

## Section S3: Powder Diffraction

Another one of the BCDI experimental prerequisites is to use single crystalline nanoflake or nanocrystals. We performed powder x-ray diffraction to characterize and understand the structural make up of the grown nanoflakes. As seen from Figure S1 (b), the flakes used are single crystalline with preferred orientation around the (002) planes. This informs us which area in the reciprocal space to focus on in order to collect the 3D data for reconstructions.

## Section S4: X-ray Photoelectron Spectroscopy

In these sets of experiments both Raman spectroscopy as well as BCDI, the main aim was to study Light-Matter interactions. Hence we are required to understand and know the chemical make up as well as the oxidation states in the nanoflake. X-ray photoelectron spectroscopy is a materials characterization technique which uses elec- trons and generated x-rays to determine the oxidation states of surface or near surface elements. Here we con- firm our assumption that the stoichiometry of surface matches with *CsBiNb*_2_*O*_7_ confirming the oxidation states of the surface ions as shown in Figure S2 (a).

## Section S5: Transmission Measurement

In order to perform light matter interaction and confirm that the interaction that we observed a completely struc- tural interaction, we needed to confirm the bandgap of the CBNO nanoflake. We performed transmittance and absorbance measurement showing the bandgap of the synthesized nanocrystal. We confirmed it to be similar to the one shown by Shi et.al. This is shown in Figure S2(b).

## Section S6: In-Operando Bragg CDI with Twisted Light

The set-up shown in Figure S3 was used to continuously illuminate the sample as it was rocked for collecting Diffraction data. The light travelling out of the laser source travels through a beam expander on to a spatial light modulator (SLM), which is then directed towards the periscope using a beam split-er. Further it is bounced up to the height of the microscope light source (which is then replaced by the laser). An Ultra-Violet laser of 375nm, guided via a confocal system available at the 34-IDC beamline, was focused down to 10 *µ*m a normal incidence to the isolated CBNO flake of interest. During the collection of rocking curves, the sample was continuously il- luminated. We employed a spatial light modulator (SLM) to select the optical beam’s topological charge, thereby controlling the OAM and torque transferred to the crystal.

To track the time evolution of the ferroelectric displacement field in CBNO flakes under twisted light illumina- tion with an OAM field, 3D diffraction data was collected near the (002) reciprocal lattice point with the crystal subjected to a cyclical application of OAM beam topological charges. The crystal was subjected to time dura- tions of *t*_0_, *t*_1_, *t*_2_, and *t*_3_ ≈ 30 minutes of continuous UV light illumination corresponding to *ℓ* = 0, *ℓ* = 1, *ℓ* = −1,

and back to *ℓ* = 0’.

## Section S7: Twisted Light Electric Field Calculations

Due to the donut-shaped intensity profile of OAM-carrying light, it can be seen that the magnitude of the electric field is minimal at the center of the beam, increase to maximum at the center of the ring, and decrease toward the edge of the beam, shown in Fig S6. A 3D modelling and comparison between Gaussian beam and *LG*_10_ is shown in Fig S4, including electric field distribution, intensity profile, phase profile, and Poynting vector (see Fig S5)^[[2]](#endnote-2)^. The in-plane component of the electric field is parallel to the surface of the beam, and the out-of- plane component of the electric field is perpendicular to the surface of the beam^[[3]](#endnote-3)^. Fig S7 demonstrates both the out-of-plane radiation force and the in-plane rotational force experienced by a particle from twisted beam^[[4]](#endnote-4)^.

The power *P* of each LG beam used in our experiments is measured and shown in Table 1. Power of LG is in- dependent of z and conserved along the z-axis ^[[5]](#endnote-5)^. In principle, the power of any paraxial light can be calculated as summation of its intensity distribution over the beam’s area^[[6]](#endnote-6)^:

$\begin{aligned} \mathcal{P=}\int I\cdot d\boldsymbol{A}= \int_{-\infty}^{\infty} \boldsymbol{E}^{*}\boldsymbol{E}dxdy \#\left( 1 \right) \end{aligned}$

Here, we aim to leverage Equation 1 to calculate the experimental value of TL’s electric field. We generated LG beams whose beam waist is *ω*_0_ = 1*µ*m with calculation resolution of 10 nm. Using the recorded power values, intensity values are assigned according the beams’ theoretical intensity distributions. Electric field distribution is calculated using *I*(*r*) = |**E**(*r*)|^2^. Summation of |**E**(*r*)| over area yielded the final experimental electric flux, which are shown in Table 1.

While only TLs of topological charge 0, 1, and -1 are used to interact with CBNO samples, we also take power measurement for beam of other topological charges. In order to deepen the analysis, we present our model of other LG beams using these measurements in Figure S6. Figure S6a and b show the intensity distribution of an OAM-carrying beam and intensity line profile of TLs with *ℓ* from 0 to 5. As presented, due to beam of higher topological charge rotate at higher angular velocity, its singularity possesses a large radius, causing the beam’s profile to be a thinner ring despite having a larger overall radius. Consequently, the power of an OAM-carrying beam does not increase linearly with the magnitude of the topological charge. Eventually, we would not ob- serve a significant increase in power despite an increase of *ℓ*. This is also reflect in electric flux of LG beams as

a function of *ℓ*, shown in Figure S6c. Finally, from information shown in Figure S6, we conclude that the biggest change in the effect of electric field exists between ${LG}_{\begin{aligned} 0 \\ \\ \end{aligned}}^{-1}$, ${LG}_{\begin{aligned} 0 \\ \\ \end{aligned}}^{0}$, and ${LG}_{\begin{aligned} 0 \\ \\ \end{aligned}}^{1}$, which is why these beams are used in the experiments regarding CBNO.

## Section S8: Electrostatic Force and Torque Acting on Nanoflake under OAM Electric Field

Given the experimental observations with the CsBiNb_2_O_7_ (CBNO) nanoflake under the influence of twisted or- bital angular momentum (OAM) light, we need to develop a theoretical framework to describe the electrostatic forces and torques acting on the nanoflake in both homogeneous (*ℓ* = 0) and inhomogeneous (*ℓ* = ±1) electric fields. This framework will help interpret the displacement fields, Bloch point (BP) transitions, and strain evo- lutions observed experimentally. The decision to classify an electric field as homogeneous or inhomogeneous

depends on the spatial distribution and variation of the field on the nanoflake (or regions of). When dealing with light sources, such as lasers, a plane wave or Gaussian beam that is far from its focus typically generates a nearly homogeneous electric field over small regions

For an OAM beam with a topological charge *ℓ* = 0, (see Figure S4) the field is not twisted, and the electric field distribution is similar to a standard Gaussian beam, which can be approximated as homogeneous if observed over a small enough region relative to the beam waist. For OAM beams with non-zero topological charge (*ℓ* ≠ 0), (see Figure S4) the electric field has a helical phase structure and varies azimuthally around the beam axis. This naturally leads to an inhomogeneous electric field in the plane perpendicular to the beam’s propagation di- rection. The higher the topological charge *ℓ*, the more pronounced the inhomogeneity in the electric field. The inhomogeneity is particularly significant near the beam’s center, where the field intensity may have an optical vortex phase structure with a zero-intensity core.

To derive the electrostatic force **F** acting on the nanoflake in a homogeneous electric field, we need to consider the principles of electrostatics and the interaction between the OAM electric field and the FE polarization in CBNO.

The polarization **P**(**r**) at a point **r** is defined as the dipole moment per unit volume. In the presence of an exter-

nal OAM electric field $\boldsymbol{E}_{OAM}^{ext}$, the polarization interacts with the field, which can result in forces and torques on

the CBNO crystal.

The electrostatic force density **f**(**r**) in the CBNO crystal is given by the expression:

$$\begin{aligned} \boldsymbol{f}\left( \boldsymbol{r} \right)= \rho\left( \boldsymbol{r} \right)\boldsymbol{E}_{OAM}^{ext}\left( \boldsymbol{r} \right)+\left( \boldsymbol{P}\left( \boldsymbol{r} \right)\cdot\nabla\right)\boldsymbol{E}_{OAM}^{ext}\left( \boldsymbol{r} \right) \boldsymbol{\#}\left( 2 \right) \end{aligned}$$

where

- - *ρ_f_* (**r**) = −∇· **P**(**r**) is the bound charge density.

The first term represents the force due to the interaction of the electric field with the bound charges, and the sec- ond term represents the force due to the interaction of the polarization with the spatial gradient of the electric field. The total force **F** on the entire nanoflake can be obtained by integrating the force density **f**(**r**) over the vol-ume *V* of the nanoflake:

$$\begin{aligned} \boldsymbol{F}= \int_{V}^{0} \boldsymbol{f}\left( \boldsymbol{r} \right)dV \boldsymbol{\#}\left( 3 \right) \end{aligned}$$

Substituting the expression for **f**(**r**), we get:

$$\begin{aligned} \boldsymbol{F}= \int_{V}^{0} \left[ \rho\left( \boldsymbol{r} \right)\boldsymbol{E}_{OAM}^{ext}\left( \boldsymbol{r} \right)+\left( \boldsymbol{P}\left( \boldsymbol{r} \right)\cdot\nabla\right)\boldsymbol{E}_{OAM}^{ext}\left( \boldsymbol{r} \right) \right]dV\boldsymbol{\#}\left( 4 \right) \end{aligned}$$

Since the observed vortex core as no free space charge, the first term involving *ρ_f_* (**r**) integrates to zero, leaving us with:

$$\begin{aligned} \boldsymbol{F}=\int_{V}^{0} \left[ \left( \boldsymbol{P}\left( \boldsymbol{r} \right)\cdot\nabla\right)\boldsymbol{E}_{OAM}^{ext}\left( \boldsymbol{r} \right) \right]dV \#\left( 5 \right) \end{aligned}$$

## Nanoflake and Vortex Stabilization in a Homogeneous Electric Field (*ℓ* = 0)

When the nanoflake is illuminated with an OAM beam with *ℓ* = 0, the electric field is expected to be homogeneous across the sample. This corresponds to a scenario where the electric field $\boldsymbol{E}_{OAM}^{ext}$ does not vary spatially, meaning ${\nabla\boldsymbol{E}}_{OAM}^{ext}\boldsymbol{=}0$. This shows that no translational force acts on the nanoflake in a homogeneous field. Thus, the FE vortex core is stable under (*ℓ* = 0) as shown in Fig3.

**Electrostatic Torque (M)**

The torque on the nanoflake due to a homogeneous electric field can be expressed as:

$$\begin{aligned} \boldsymbol{M=}\int_{\boldsymbol{V}}^{\boldsymbol{0}} \left[ \boldsymbol{P}\left( \boldsymbol{r}^{\boldsymbol{'}} \right)\boldsymbol{\times}\boldsymbol{E}_{OAM}^{ext}\left( \boldsymbol{r}^{\boldsymbol{'}} \right) \right]dV^{'}\boldsymbol{\#}\left( 6 \right) \end{aligned}$$

Since $\boldsymbol{E}_{OAM}^{ext}$ is uniform, the torque simplifies to:

$$\begin{aligned} \boldsymbol{M= -}\boldsymbol{E}_{OAM}^{ext}\left( \boldsymbol{r}^{\boldsymbol{'}} \right)\boldsymbol{\times}\int_{\boldsymbol{V}}^{\boldsymbol{0}} \boldsymbol{P}\left( \boldsymbol{r}^{\boldsymbol{'}} \right)dV^{'}\boldsymbol{\#}\left( 7 \right) \end{aligned}$$

In a uniform field, if the polarization vortex is symmetrically distributed, $\int_{\boldsymbol{V}}^{\boldsymbol{0}} \boldsymbol{P}\left( \boldsymbol{r}^{\boldsymbol{'}} \right)dV^{'}\boldsymbol{=}0$, leading to **M** = 0. However, slight asymmetries can induce a small torque, causing the vortex to rotate around its axis.

## Nanoflake in an Inhomogeneous Electric Field (*ℓ* = ±1)

When the nanoflake is exposed to an OAM beam with *ℓ* = ±1, the electric field is inhomogeneous, varying spa- tially within the sample. This inhomogeneity introduces more complex interactions, resulting in both electro- static force and torque.

**Inhomogeneous Electric Field Profile**

For *ℓ* = ±1, the electric field, the electric field $\boldsymbol{E}_{OAM}^{ext}\left( \boldsymbol{r}^{\boldsymbol{'}} \right)$ can be modeled as:

$$\begin{aligned} \boldsymbol{E}_{OAM}^{ext}\left( \boldsymbol{r}^{\boldsymbol{'}} \right)=E_{0}\left( \boldsymbol{r}^{\boldsymbol{'}} \right)\left[ \cos\left( \theta\left( \boldsymbol{r}^{\boldsymbol{'}} \right) \right){\hat{\boldsymbol{e}}}_{\boldsymbol{x}}+ \sin\left( \theta\left( \boldsymbol{r}^{\boldsymbol{'}} \right) \right){\hat{\boldsymbol{e}}}_{\boldsymbol{y}} \right],\boldsymbol{\#}\left( 8 \right) \end{aligned}$$

where $E_{0}\left( \boldsymbol{r}^{\boldsymbol{'}} \right)$ varies with position $\boldsymbol{r}^{\boldsymbol{'}}$and $\theta\left( \boldsymbol{r}^{\boldsymbol{'}} \right)$represents the azimuthal angle, capturing the twisting nature of the field.

**OAM Electrostatic Force (F) and Torque**

The electrostatic force in an inhomogeneous field is:

$$\begin{aligned} \boldsymbol{F}=\int_{V}^{0} \left[ \left( \boldsymbol{P}\left( \boldsymbol{r}^{\boldsymbol{'}} \right)\cdot\nabla\right)\boldsymbol{E}_{OAM}^{ext}\left( \boldsymbol{r}^{\boldsymbol{'}} \right) \right]dV^{'} \#\left( 9 \right) \end{aligned}$$

Due to the spatial variation in $\boldsymbol{E}_{OAM}^{ext}$ this force is generally non-zero, driving translational motion of the

nanoflake. The presence of vortex-antivortex pairs, as observed experimentally, indicates significant internal strain and localized gradients in the electric field, which contribute to this force.

The torque in the inhomogeneous field is given

$$\begin{aligned} \boldsymbol{M}=\int_{V}^{0} \left( \left[ \boldsymbol{P}\left( r^{'} \right)\times\boldsymbol{E}_{OAM}^{ext}\left( \boldsymbol{r}^{\boldsymbol{'}} \right) \right] \right)\boldsymbol{+[}\boldsymbol{r}^{\boldsymbol{'}}\times(\boldsymbol{P(}\boldsymbol{r}^{\boldsymbol{'}}\boldsymbol{)\cdot}\nabla)\boldsymbol{E}_{OAM}^{ext}\left( \boldsymbol{r}^{\boldsymbol{'}} \right)\boldsymbol{]}dV^{'}\boldsymbol{\#}\left( 10 \right) \end{aligned}$$

The second term accounts for the inhomogeneous nature of the field, introducing additional complexity into the torque. The inhomogeneous field can lead to significant rotational motion, especially if there are large spatial

variations in $\boldsymbol{E}_{OAM}^{ext}$.

## Correlation with Experimental Observations

- - **Bloch and Anti-Bloch Point Transitions (Fig. 4a, 4b):** The inhomogeneous electric field (*ℓ* = ±1) pro- motes the formation of Bloch and anti-Bloch points, driven by the localized variations in polarization and strain. These topological features correspond to regions where the electrostatic force and torque vary sig-

nificantly, explaining the observed transitions.

- - **Strain Evolution (Fig. 4c, 4d):** The hysteresis-like behavior in strain under varying OAM fields suggests that the nanoflake’s response is non-linear, with different strain states correlating with distinct polarization configurations. This strain is a direct result of the inhomogeneous electrostatic interactions.
  - **Homogeneous Field (***ℓ* = 0**):** The electrostatic force is zero, and torque is minimal unless there are asym- metries in the polarization distribution.
  - **Inhomogeneous Field (***ℓ* = ±1**):** Both electrostatic force and torque are significant, leading to complex dynamics such as the formation of vortex-antivortex pairs, Bloch point transitions, and strain-induced topo- logical changes.

## Section S9: DFT calculations of the Band Structure in CBNO

We performed first-principles calculations using the CASTEP (Cambridge Serial Total Energy Package) ^[[7]](#endnote-7)^ and Quantum ESPRESSO (QE) ^[[8]](#endnote-8)^ packages. Calculations involving the application of an external light beam car- rying Orbital Angular Momentum (OAM) with different topological charges (*l*) were performed using CASTEP. The experimental electric field component of the OAM beam was explicitly included in these calculations.

We used the Perdew-Burke-Ernzerhof (PBEsol) exchange correlation functional within the generalized gradient approximation (GGA) ^[[9]](#endnote-9)^ for all calculations. We used norm-conserving pseudopotentials and a plane-wave ba- sis set with a kinetic energy cutoff of 600 eV. The pseudopotentials for Cs, Bi, Nb, and O were taken from the CASTEP library.

The Brillouin zone was sampled using a 6 × 6 × 6 Monkhorst-Pack grid ^[[10]](#endnote-10)^. The self-consistent field (SCF) cal- culations converged to an energy threshold of 1 × 10^−6^ eV.

The initial molecular structure of CsBiNb_2_O_7_ was obtained from experimental theoretical data predictions avail-

able on the Materials Project. The structures were optimized using density functional theory (DFT) as imple- mented in Quantum ESPRESSO. The Perdew-Burke-Ernzerhof (PBEsol) exchange-correlation functional was chosen to describe the electron-electron interactions. The unit cell parameters are: *a* = 11*.*87380 Å, *b* = 5*.*45730

Å, *c* = 5*.*55750 Å, with angles *α* = 90*.*000^◦^, *β* = 90*.*000^◦^, *γ* = 90*.*000^◦^. The atomic positions were fully relaxed

until the forces in each atom were less than 0.01 eV/Å, and the stress tensor was below 0.001 GPa.

Polarizability was computed using density functional perturbation theory (DFPT) ^[[11]](#endnote-11)^. IR and Raman spectra were obtained by calculating the dynamical charges and Raman tensors, respectively. The dielectric function and Raman activities were computed to simulate the IR and Raman spectra.

To understand the electronic motif behind the results, we performed Density Functional theory (DFT) calcula- tions using quantum espresso a freely available software. We first started by stabilizing the structure as shown in Figure S8 and then calculate the Band structure and the contribution to the density of the states of different atoms. The smaller area of the band structure around the gamma point was used in the tight binding model to observe chiral band structure in CBNO when illuminated with twisted light. We also calculate the ferroelectric

displacements for different atomic species in the CBNO unit cell as shown in Figure S9.In order to check for the defect states in the system, we performed SCF calculations and Hubbard calculation on the pristine as well as oxygen defect induced system as shown in Figure S12 We also use the calculated electric fields indicated in Ta- ble S1 along with the experimental strain observed in Figure S21 to calculate the effect on unit cell structures in CBNO as shown in Figure S10. In order to confirm the experimentally obtained Raman data, we use the SCF stabilized unit cell to calculate the phonon band structure as shown in Figure S11 and the phonon density of the states in Figure S12.

Phonon dispersion curves for CsBiNb_2_O_7_, calculated using density functional perturbation theory (DFPT). The horizontal axis represents the wave vector along high-symmetry directions in the Brillouin zone, while the verti- cal axis denotes the phonon frequencies in inverse centimeters (cm^−1^).

The presence of acoustic phonon branches is observed, which exhibit linear behavior near the Γ point. These branches are essential for understanding the sound velocity and mechanical properties of the material. Optical modes at the Γ, M, and R points $\left( 0,0,0;\frac{1}{2},\frac{1}{2},0;\frac{1}{2},\frac{1}{2},\frac{1}{2} \right)$ involve vibrations of atoms that lead to changes in the polarization of the crystal lattice. Notable gaps between the acoustic and optical phonon branches indicate regions of phonon band gaps, where there are no phonon states. These gaps can influence the thermal conductivity and other properties of the ma- terial. Additionally, negative frequencies are observed at all points along the edges of the Brillouin zone, indi- cating potential dynamic instability within the crystal structure due to the softening of the optical mode. This suggests that the crystal may undergo structural changes under certain conditions. The primary order parameter driving the transition is not directly related to strain, but to a soft mode at a specific point in the Brillouin zone. This provides insights into the nature and mechanism of the improper ferroelastic phase transition, indicating that the phase transition is driven by the instability of a specific vibrational mode.

The density of phonon states (DOS) for CsBiNb_2_O_7_, also calculated using DFPT. The horizontal axis represents the phonon frequencies in cm^−1^, while the vertical axis denotes the density of states (DOS) in (1/cm^−1^).

Key features of the DOS include peaks at specific frequencies corresponding to the vibrational modes of the atoms within the crystal lattice. Distinct peaks at higher frequencies are primarily associated with the optical phonon modes, which are critical for understanding interactions involving higher energy vibrations. Lower fre- quency regions are dominated by contributions from acoustic phonon modes, essential for understanding low- energy excitations and heat capacity at low temperatures.

Gaps in the DOS indicate gaps in the phonon band that separate different vibrational modes. These gaps can have significant implications for the thermal and electronic properties of the material. The DOS provides valu- able information on the vibrational properties of CsBiNb_2_O_7_ and can be used to predict phase stability and po- tential phase transitions.

The presence of soft modes (negative or very low-frequency modes) indicates potential phase instability, sug- gesting that the crystal structure may undergo a phase transition under certain conditions. By comparing the DOS of different structural modifications, one can identify which structures are dynamically stable (absence of soft modes) and which are prone to phase transitions. The distribution and characteristics of the DOS peaks pro- vide information on how atomic vibrations contribute to the overall stability of the structure, helping to under- stand which structural modifications are more thermodynamically favorable.

Examining phonon dispersion and DOS provides comprehensive information on the vibrational properties, dy- namic stability, and potential phase transitions of CsBiNb_2_O_7_. This detailed understanding is crucial for the de- velopment of advanced electronic devices and other applications where material stability and performance are paramount.

## Section S10: OAM Induced Chiral Band Structure in CsBiNb_2_O_7_ (CBNO)

CsBiNb_2_O_7_ (CBNO) is a material composed of layers of Bi-O octahedra. These Bi-O layers can form a chiral structure when subjected to twisting induced by Orbital Angular Momentum (OAM) light.

The chiral nature of the molecule arises from the arrangement of atoms within one helical unit cell. Consider a helical unit cell with *N* atoms, where *N* determines the handedness (right or left) of the chiral structure. Each atom is connected to its nearest neighbor by a bond length *bond*, projected in the xy-plane. Additionally, there is a projected bond length *δ_z_* along the z-axis.

The interaction between the twisted OAM light and CsBiNb_2_O_7_ involves the OAM of light being transferred to the electrons within the material. This transfer of OAM influences the electronic states within the material, lead- ing to a chiral band structure.

To describe this phenomenon, we use the tight-binding method, a common approach in solid-state physics. The tight-binding Hamiltonian for the chiral molecule includes terms for spin, orbital angular momentum (AM), and spin-orbit coupling (SOC). In the basis of pxyz orbitals, the matrices for spin and orbital AM are constructed as follows ^[[12]](#endnote-12)^:

$$\begin{aligned} L_{0}= \left( \begin{matrix} \begin{matrix} 1 \\ 0 \\ \begin{matrix} 0 \\ 0 \\ \begin{matrix} 0 \\ 0 \end{matrix} \end{matrix} \end{matrix} & \begin{matrix} 0 \\ 1 \\ \begin{matrix} 0 \\ 0 \\ \begin{matrix} 0 \\ 0 \end{matrix} \end{matrix} \end{matrix} & \begin{matrix} \begin{matrix} 0 \\ 0 \\ \begin{matrix} 1 \\ 0 \\ \begin{matrix} 0 \\ 0 \end{matrix} \end{matrix} \end{matrix} & \begin{matrix} \begin{matrix} 0 \\ 0 \\ 0 \end{matrix} \\ 1 \\ \begin{matrix} 0 \\ 0 \end{matrix} \end{matrix} & \begin{matrix} \begin{matrix} \begin{matrix} 0 \\ 0 \\ 0 \end{matrix} \\ 0 \\ \begin{matrix} 1 \\ 0 \end{matrix} \end{matrix} & \begin{matrix} \begin{matrix} 0 \\ 0 \end{matrix} \\ 0 \\ \begin{matrix} 0 \\ 0 \\ 1 \end{matrix} \end{matrix} \end{matrix} \end{matrix} \end{matrix} \right), L_{z}= \left( \begin{matrix} \begin{matrix} 0 \\ 0 \\ \begin{matrix} i \\ 0 \\ \begin{matrix} 0 \\ 0 \end{matrix} \end{matrix} \end{matrix} & \begin{matrix} 0 \\ 0 \\ \begin{matrix} 0 \\ i \\ \begin{matrix} 0 \\ 0 \end{matrix} \end{matrix} \end{matrix} & \begin{matrix} \begin{matrix} -i \\ 0 \\ \begin{matrix} 0 \\ 0 \\ \begin{matrix} 0 \\ 0 \end{matrix} \end{matrix} \end{matrix} & \begin{matrix} \begin{matrix} 0 \\ -i \\ 0 \end{matrix} \\ 0 \\ \begin{matrix} 0 \\ 0 \end{matrix} \end{matrix} & \begin{matrix} \begin{matrix} \begin{matrix} 0 \\ 0 \\ 0 \end{matrix} \\ 0 \\ \begin{matrix} 0 \\ i \end{matrix} \end{matrix} & \begin{matrix} \begin{matrix} 0 \\ 0 \end{matrix} \\ 0 \\ \begin{matrix} 0 \\ -i \\ 0 \end{matrix} \end{matrix} \end{matrix} \end{matrix} \end{matrix} \right), L_{x}= \left( \begin{matrix} \begin{matrix} 0 \\ 0 \\ \begin{matrix} 0 \\ 0 \\ \begin{matrix} i \\ 0 \end{matrix} \end{matrix} \end{matrix} & \begin{matrix} 0 \\ 0 \\ \begin{matrix} 0 \\ 0 \\ \begin{matrix} 0 \\ i \end{matrix} \end{matrix} \end{matrix} & \begin{matrix} \begin{matrix} 0 \\ 0 \\ \begin{matrix} 0 \\ 0 \\ \begin{matrix} 0 \\ 0 \end{matrix} \end{matrix} \end{matrix} & \begin{matrix} \begin{matrix} 0 \\ 0 \\ 0 \end{matrix} \\ 0 \\ \begin{matrix} 0 \\ 0 \end{matrix} \end{matrix} & \begin{matrix} \begin{matrix} \begin{matrix} -i \\ 0 \\ 0 \end{matrix} \\ 0 \\ \begin{matrix} 0 \\ 0 \end{matrix} \end{matrix} & \begin{matrix} \begin{matrix} 0 \\ -i \end{matrix} \\ 0 \\ \begin{matrix} 0 \\ 0 \\ 0 \end{matrix} \end{matrix} \end{matrix} \end{matrix} \end{matrix} \right), L_{y}= \left( \begin{matrix} \begin{matrix} 0 \\ 0 \\ \begin{matrix} 0 \\ 0 \\ \begin{matrix} 0 \\ -i \end{matrix} \end{matrix} \end{matrix} & \begin{matrix} 0 \\ 0 \\ \begin{matrix} 0 \\ i \\ \begin{matrix} 0 \\ 0 \end{matrix} \end{matrix} \end{matrix} & \begin{matrix} \begin{matrix} 0 \\ 0 \\ \begin{matrix} 0 \\ 0 \\ \begin{matrix} 0 \\ 0 \end{matrix} \end{matrix} \end{matrix} & \begin{matrix} \begin{matrix} 0 \\ 0 \\ 0 \end{matrix} \\ 0 \\ \begin{matrix} i \\ 0 \end{matrix} \end{matrix} & \begin{matrix} \begin{matrix} \begin{matrix} 0 \\ 0 \\ 0 \end{matrix} \\ -i \\ \begin{matrix} 0 \\ 0 \end{matrix} \end{matrix} & \begin{matrix} \begin{matrix} i \\ 0 \end{matrix} \\ -i \\ \begin{matrix} 0 \\ 0 \\ 0 \end{matrix} \end{matrix} \end{matrix} \end{matrix} \end{matrix} \right)\#\left( 11 \right) \end{aligned}\text{ }$$

The SOC Hamiltonian in the pxyz basis is given by:

$$\begin{aligned} H_{SOC}= \left( \begin{matrix} \begin{matrix} 0 \\ 0 \\ \begin{matrix} i\sigma_{z} \\ 0 \\ \begin{matrix} 0 \\ 0 \end{matrix} \end{matrix} \end{matrix} & \begin{matrix} 0 \\ 0 \\ \begin{matrix} 0 \\ i\sigma_{x} \\ \begin{matrix} 0 \\ 0 \end{matrix} \end{matrix} \end{matrix} & \begin{matrix} \begin{matrix} -i\sigma_{z} \\ 0 \\ \begin{matrix} 0 \\ 0 \\ \begin{matrix} 0 \\ 0 \end{matrix} \end{matrix} \end{matrix} & \begin{matrix} \begin{matrix} 0 \\ -i\sigma_{x} \\ 0 \end{matrix} \\ 0 \\ \begin{matrix} 0 \\ 0 \end{matrix} \end{matrix} & \begin{matrix} \begin{matrix} \begin{matrix} 0 \\ 0 \\ 0 \end{matrix} \\ 0 \\ \begin{matrix} 0 \\ i\sigma_{z} \end{matrix} \end{matrix} & \begin{matrix} \begin{matrix} 0 \\ 0 \end{matrix} \\ 0 \\ \begin{matrix} 0 \\ -i\sigma_{z} \\ 0 \end{matrix} \end{matrix} \end{matrix} \end{matrix} \end{matrix} \right) \#\left( 12 \right) \end{aligned}$$

The tight-binding Hamiltonian for both intra and inter unit cells is then constructed. The band structure is ob- tained by solving the eigenvalue problem for different values of *k_z_* (momentum along the z-axis). The result- ing chiral band structure displays ranges of energy bands and band gaps, indicative of chirality in the electronic states.

In conclusion, when twisted by OAM light, the Bi-O layers in CsBiNb_2_O_7_ form a chiral molecule with a unique band structure, influenced by the OAM of light. This chiral band structure(see Figure S14) is a manifestation of the intricate interplay between the electronic properties and the chiral molecular arrangement in the material.

## Section S11: Diffraction Analysis and Pixel Size Calculations:

To understand the structural changes in the material under continuous illumination, we performed a BCDI exper- iment followed by the reciprocal space diffraction analysis. 9.0 keV coherent X-ray photons were selected using an Advanced Photon Source Si (111) monochromator sector 34-ID-C. The beam’s transverse coherence length was 0.7 µm, and its energy bandwidth of 1 eV defined its monochromaticity. A pair of Kirkpatrick-Baez mirrors placed after the beam-defining aperture focused the X-rays onto the sample. For this experiment, the beam size was 700 nm by 700 nm. The confocal mirror at the 34-ID-C beamline was modified as shown in Figure S3 to perform in-situ BCDI measurements. A Medipix2 CMOS X-ray detector was able to be positioned around the diffraction sphere using a motorized arm. We align the position of the detector with the CBNO sample’s outgo- ing (002) characteristic Bragg reflection. To zoom into the interference fringes in the diffraction pattern, the de- tector was positioned 1.2 meters away from the sample. Deployed in the sample-to-detector path, an evacuated flight tube reduces the amount of photons lost through air scattering. Using an evacuated flight tube, having a high sensor gain, and using the detector’s photon counting mode is essential for photon-starving techniques like nanoscale Bragg coherent diffractive imaging or similar methods^[[13]](#endnote-13),^^[[14]](#endnote-14)^.

The Rocking curve was collected as a collective of the 2D diffraction patterns in the vicinity of the 002 Bragg peak corresponding to 2*θ* = 13*.*5 degrees. with a scanning range of about ∆*θ* = 0*.*36 degrees about the Bragg peak origin. Throughout a single curve about 360 patterns were collected. A total of 4 such 3D scans were col- lected for *ℓ* going from 0->1->-1->0.

We observe the emergence of a long-range ordering from Gaussian light to light carrying a topological charge of 1. We see that this change is not reversible in reciprocal space. We also observe an angular changes in the *Q_z_* direction as seen in Figure S15 We also observe a change in the d spacing as we cycle the particle from 0->1->- 1->0’ as seen in Figure S16.

## Section S12: Reconstruction and Pixel Size Calculations

These recorded 3D diffraction patterns(see Figure S15) are then inverted to real space 3D image of the nanopar- ticle after the phase Φ and amplitude *A* of the complex wavefield given by *ρ*(*r*) = *A*(*r*)*e*^(^*^i^*^Φ(^*^r^*^))^. The reconstructed

phase Φ scales linearly with the displacement field *u*_002_ for a given reciprocal lattice vector *G*_002_ given by Φ(*r*) =

*u*_002_*.G*_002_. The Bragg Electronic Density helps reconstruct the 3D shape of the particle as shown in figure S16.

The reconstruction allows us to slice through the volume of the nanoparticle under a certain topological charge and analyze the signatures of the topological charges.

Iterative phase retrieval algorithms based on Fienup’s Hybrid Input-output (HIO) method with Error Reduc- tion(ER) were used^[[15]](#endnote-15)^. When the measurement points are close enough to one another to satisfy the oversam- pling requirement, an important step in the process is to reverse the diffraction data using a computer algorithm. The first stage is to assume a three-dimensional support volume where all sample densities will be restricted to exist. With the support constraint imposed in the real space and the intensity mask constraint in the reciprocal space, these methods impose a backward and forward Fourier transform between them. A total of 200 itera- tions consisting of 80 HIO and 120 ER were performed on the measured 3D diffraction patterns with an average

*χ*2 = 0*.*01 which translates to about 1% of the error. The reconstructed phases were then unwrapped to obtain the ferroelectric displacement fields. An estimate of the resolution resulting from the reconstruction obtained us- ing Phase retrieval Transfer Function as shown in Figure S28 which indicates a resolution of about 33 nm.

Next, to understand the implications of the above observations in real space, we performed phase retrieval using the Error Reduction and High Input-Output iterative methods. We observe the changes in the Bragg Electronic Density of the particle as shown in the iso-surfaces given in Figure S17

The real space pixel size, of the reconstructions can be determined using the following. **Given Parameters:**

- *λ* (X-ray wavelength in meters).
- *D* (Sample-to-detector distance in meters).
- *d* (Pixel size of the detector in meters).
- ∆*θ* (Step size of the rocking curve scan in degrees).
- *N_x_* (Array size of the data in the X-direction).
- *N_y_* (Array size of the data in the Y-direction).
- *N_z_* (Array size of the data in the Z-direction for 3D experiments).

## Step-by-step Derivation:

1. **X-direction (lateral direction):**

The real space pixel size (pixel_size_x) in the X-direction can be derived as follows:

The angular dispersion ∆*θ* between consecutive pixels in the rocking curve is given in degrees. To convert this angular dispersion to radians, we use the conversion factor: $\frac{\pi}{180}$. So, ∆*θ* in radians is given by:

$$\Delta\theta_{rad}= \Delta\theta\times\frac{\pi}{180}$$

Now, the spatial dispersion ∆*x* between consecutive pixels in the X-direction is related to the pixel size of the detector (*d*) and the sample-to-detector distance (*D*) by the equation:

$$\Delta x= \frac{d}{D}$$

The real space pixel size in the X-direction (pixel_size_x) is then calculated using the wavelength (*λ* ) and the spatial dispersion (∆*x*) as follows:

$$pixel\_size\_x= \frac{\lambda\cdot\Delta x}{N_{x}}$$

## Y-direction (vertical direction):

The real space pixel size (pixel_size_y) in the Y-direction can be derived similarly to the X-direction. The an- gular dispersion ∆*θ* is already converted to radians, and the spatial dispersion ∆*y* between consecutive pixels in the Y-direction is also related to the pixel size of the detector (*d*) and the sample-to-detector distance (*D*) by the equation:

$$\Delta y= \frac{d}{D}$$

The real space pixel size in the Y-direction (pixel_size_y) is then calculated using the wavelength (*λ* ) and the spatial dispersion (∆*y*) as follows:

$$pixel\_size\_y= \frac{\lambda\cdot\Delta y}{N_{y}}$$

**3. Z-Direction (depth direction)**

For 3D experiments (Nx, Ny, and Nz), we also consider the Z-direction (depth direction) along which the rock- ing curve is scanned. The angular dispersion ∆*θ* is converted to radians as before, and the spatial dispersion ∆*z* between consecutive pixels in the Z-direction is related to the pixel size of the detector (*d*) and the sample-to- detector distance (*D*) by the equation:

$$\Delta y= \frac{d}{D}$$

The real space pixel size in the Z-direction (pixel_size_z) is then calculated using the wavelength (*λ* ) and the spatial dispersion (∆*z*) as follows:

$$pixel\_size\_z= \frac{\lambda}{N_{z}\cdot\Delta\theta_{rad}}$$

These derivations represent the conversion from reciprocal space to real space pixel sizes in the three spatial di- rections: X, Y, and Z. The calculation accounts for the X-ray wavelength (*λ* ), pixel size (*d*), sample-to-detector distance (*D*), and the array sizes in the corresponding directions (Nx, Ny, Nz). In the case of these experiments, the real space pixel size calculations are performed using the information mentioned in section S6 comes out to be 16 nm across the three directions.

## Section S13: Ferro-electric Materials’ Energy Landscape and Vortex tracking

We observe that on illumination with Gaussian light the reconstructed particle has a ferroelectric vortex structure from its displacement field. Now, when we illuminate it with light carrying an Orbital Angular Momentum, we push it into a metastable state as shown in Figure 2. Due to this the vortex is displaced temporarily, and then it comes back to when we illuminate with the opposite orbital angular momentum as seen in Figure S18

## Section S14: Determination of strain from Coherent diffraction datasets

We further calculate the strain evolution in the particle as a function of the applied topological charge. We use the differential Bragg’s law we calculate the strain in the particle with respect to the data collected for Gaussian light as shown below:

$$\begin{aligned} \varepsilon= \frac{\left| d^{\left( l=0 \right)}-d^{\left( l=l_{i} \right)} \right|}{d^{\left( l=0 \right)}} \#\left( 13 \right) \end{aligned}$$

We observe a reversible behavior in the strain as well, as shown in Figure S18

## Section S15: Determination of strain from Reconstructed Datasets

The real space strain at the local structural level is calculated using the following equation:

$$\begin{aligned} \varepsilon_{zz}= \frac{\delta u_{002}}{\delta z} \#\left( 14 \right) \end{aligned}$$

We observe the evolution of strain as a function of applied electric field and topological charge as shown in Fig- ure S20.

We also track the shear component of the strain along the (002) direction given by:

$$\begin{aligned} \varepsilon_{xy}=\frac{1}{2}\left( \frac{\delta u_{002}}{\delta x}+ \frac{\delta u_{002}}{\delta y} \right)\#\left( 15 \right) \end{aligned}$$

and shown in the Figure S22 .This indicates the changes in the shear component of strain as a function of ap- plied topological charge from the twisted light. The values of the compressive and tensile strains are shown in Tables S2 & S3

## Section S16: Determination of Polarization Magnitude from the Gradient of Bragg Electronic Density

In the case of ferroelectric materials, the Polarization is given by the following equation:

$$\begin{aligned} P\left( \boldsymbol{r} \right)\boldsymbol{=}\frac{1}{V}*\oint_{V}^{0} \boldsymbol{r}\rho\left( \boldsymbol{r} \right)d^{3}\boldsymbol{r}\#\left( 16 \right) \end{aligned}$$

where *ρ*(**r**) is regarded as the electronic density of the system. Now in case of the change in polarization or change in magnitude of the polarization, we can modify the equation shown above as follows,

$$\begin{aligned} P\left( \boldsymbol{r} \right)\boldsymbol{=}\frac{1}{V}*\oint_{V}^{0} \boldsymbol{r}\nabla\rho\left( \boldsymbol{r} \right)d^{3}\boldsymbol{r}\#\left( 17 \right) \end{aligned}$$

This indicates a change in the magnitude of polarization and its dependence on the gradient of the Bragg elec- tronic density^[[16]](#endnote-16)^.

## Section S17: Topology Analysis: Bloch Points, Anti-Bloch Points and Merons

We employ the ability of BCDI of 3D structural and strain analysis to understand the different topologies in the system. We already analyzed and discussed the topological nature of the ferroelectric vortex in the nanocrystal. We start by tracking the behavior of the reconstructed Bragg Electronic densities to track polar topologies in this 2D ferroelectric materials as shown in Figure S 24

The Blue and red dots indicate Bloch and Anti-bloch behavior of the Polarization magnitude. Further to observe the Polar topology in the displacement field which signifies the changes in spontaneous polarization giving rise to vortex and anti vortex structures. We track the said vortex structures as discussed in Section 7 and 18 we also study the quiver plots of the ferroelectric displacement fields as shown in Fig 17.

We use the changes in topologies in the displacement field to study the three dimensional nature of the toroidal moment in the nanocrystal. This change is tracked in Figure 28

**Winding Number Reduction for Ferroelectric Vortex and Other Topological Objects**

Given the winding number expression:

$$\begin{aligned} Q=\frac{1}{8\pi}\int dA_{i}\varepsilon_{ijk}\frac{\sum_{\kappa\alpha} Z_{\kappa,\alpha}^{*}u_{\kappa\alpha}\left( r \right)}{\left| \sum_{\kappa\alpha} Z_{\kappa,\alpha}^{*}u_{\kappa\alpha}\left( r \right) \right|}\cdot\left[ \partial_{j}\left( \frac{\sum_{\kappa\beta} Z_{\kappa,\beta}^{*}u_{\kappa\beta}\left( r \right)}{\left| \sum_{\kappa\beta} Z_{\kappa,\beta}^{*}u_{\kappa\beta}\left( r \right) \right|} \right)\times\partial_{k}\left( \frac{\sum_{\kappa\gamma} Z_{\kappa,\gamma}^{*}u_{\kappa\gamma}\left( r \right)}{\left| \sum_{\kappa\gamma} Z_{\kappa,\gamma}^{*}u_{\kappa\gamma}\left( r \right) \right|} \right) \right]\#\left( 18 \right) \end{aligned}$$

The polarization **P**(*r*) in terms of the displacement field is^[[17]](#endnote-17)^:

$$\begin{aligned} \boldsymbol{P}\left( r \right)= \left( \sum_{\kappa} Z_{\kappa,x}^{*}u_{\kappa x}\left( r \right), \sum_{\kappa} Z_{\kappa,y}^{*}u_{\kappa y}\left( r \right), \sum_{\kappa} Z_{\kappa,z}^{*}u_{\kappa z}\left( r \right) \right)\boldsymbol{\#}\left( 19 \right) \end{aligned}$$

The normalized polarization vector *p*ˆ(*r*) is:

$$\begin{aligned} \hat{\rho}= \frac{\boldsymbol{P}\left( r \right)}{\left| \boldsymbol{P}\left( r \right) \right|}= \frac{\left( \sum_{\kappa} Z_{\kappa,x}^{*}u_{\kappa x}\left( r \right), \sum_{\kappa} Z_{\kappa,y}^{*}u_{\kappa y}\left( r \right), \sum_{\kappa} Z_{\kappa,z}^{*}u_{\kappa z}\left( r \right) \right)}{\sqrt{\left( \sum_{\kappa} Z_{\kappa,x}^{*}u_{\kappa x}\left( r \right) \right)^{2}+ \left( \sum_{\kappa} Z_{\kappa,y}^{*}u_{\kappa y}\left( r \right) \right)^{2}+ \left( \sum_{\kappa} Z_{\kappa,z}^{*}u_{\kappa z}\left( r \right) \right)^{2}}} \#\left( 20 \right) \end{aligned}$$

For a vortex, the polarization orientation angle *θ* around the core changes continuously. The orientation angle *θ* can be defined as:

$$\begin{aligned} \theta= \arctan\left( \frac{P_{y}}{P_{x}} \right)\#\left( 21 \right) \end{aligned}$$

The gradient of *θ* is:

$$\begin{aligned} \nabla\theta=\left( \frac{\partial\theta}{\partial x},\frac{\partial\theta}{\partial y} \right)\#\left( 22 \right) \end{aligned}$$

Considering rotational symmetry, we have:

$$\begin{aligned} \partial_{j}\hat{p}\left( r \right)\approx\left( -sin \theta\frac{\partial\theta}{\partial j}, -cos \theta\frac{\partial\theta}{\partial j} \right)\#\left( 23 \right) \end{aligned}$$

The cross product term is:

$$\begin{aligned} \partial_{j}\hat{p}\left( r \right)\times\partial_{k}\hat{p}\left( r \right)= \left( -\sin\theta\frac{\partial\theta}{\partial j}, -cos \theta\frac{\partial\theta}{\partial j} \right)\times\left( -\sin\theta\frac{\partial\theta}{\partial k}, -cos \theta\frac{\partial\theta}{\partial k} \right)\#\left( 24 \right) \end{aligned}$$

This simplifies to:

$$\begin{aligned} \partial_{j}\hat{p}\left( r \right)\times\partial_{k}\hat{p}\left( r \right)=\left( \frac{\partial\theta}{\partial x}\frac{\partial\theta}{\partial y}- \frac{\partial\theta}{\partial y}\frac{\partial\theta}{\partial x} \right)\hat{z}=\left( \nabla\theta\times\nabla\theta\right)\hat{z}\#\left( 25 \right) \end{aligned}$$

So the integral reduces to the contour integral of ∇*θ* around a closed path *C*:

$$\begin{aligned} \mathcal{Q=}\frac{1}{2\pi}\oint_{C}^{0} \nabla\theta\cdot d\boldsymbol{l} \#\left( 26 \right) \end{aligned}$$

**Polar Bloch Points**

For polar Bloch points, the winding number captures the change in the polarization direction in three dimen- sions, leading to:

$$\begin{aligned} \mathcal{Q=}\frac{1}{8\pi}\int dA_{i}\varepsilon_{ijk}\frac{P_{i}\left( r \right)}{\left| P_{i}\left( r \right) \right|} \left[ \partial_{j}\frac{P_{i}\left( r \right)}{\left| P_{i}\left( r \right) \right|}\times\partial_{k}\frac{P_{i}\left( r \right)}{\left| P_{i}\left( r \right) \right|} \right]\#\left( 27 \right) \end{aligned}$$

This integrates over a surface enclosing the Bloch point, capturing the three-dimensional nature of the topologi- cal charge as shown in Figures S23 & S24.

**0.0.1 Anti-Polar Bloch Points**

For anti-polar Bloch points, the winding number captures the reversal in polarization direction as shown in Fig- ures S25 & S26. The integral remains the same but will yield a negative winding number due to the opposite ori- entation^[[18]](#endnote-18)^:

$$\begin{aligned} \mathcal{Q=-}\frac{1}{2\pi}\oint_{C}^{0} \nabla\theta\cdot d\boldsymbol{l} \#\left( 28 \right) \end{aligned}$$

**Merons and Anti-Merons**

Merons and anti-merons are characterized by half-integer winding numbers and a discontinuous polarization field. For merons:

$$\begin{aligned} \mathcal{Q=}\frac{1}{4\pi}\oint_{C}^{0} \nabla\theta\cdot d\boldsymbol{l} \#\left( 29 \right) \end{aligned}$$

For anti-merons:

$$\mathcal{Q=-}\frac{1}{4\pi}\oint_{C}^{0} \nabla\theta\cdot d\boldsymbol{l}$$

## Determining the Winding Number from Reconstructed data

As discussed earlier in section S12, the reconstructed data constitutes three different sets namely, phase, am- plitude, and support. The amplitude carries the information about the Electron density of the atoms in CBNO Flake. This gives us the ability to have a look at the Bloch or Meron-like topologies present in the flake. We use MATLAB’s gradient function to estimate the 3D gradient of the amplitude. Furthermore, to determine the wind- ing number of the Bloch and Meron-like topologies, we calculate the divergence of the gradient field determined from the amplitude. The divergence is further normalized to recognize bloch, anti-bloch, meron and anti-meron topologies. Here +1 is bloch, -1 is anti-Bloch, +0.5 is a meron and -0.5 is anti-meron. Another important piece of information that can be extracted from the reconstructed data is the vortex and anti-vortex topologies. To de- termine that, we utilize the phase data and follow the same procedure we used for amplitude. Except in the last step instead of using the divergence we use the curl of the gradient of the phase. A similar nomenclature is used for the normalized curl of the gradient of the phase with +1 as vortex and -1 being the anti-vortex.

**Section S18:OAM Raman Spectroscopy Standard Raman Spectroscopy of CsBiNb**_2_**O**_7_ **Raman Active Modes**

The symmetry of the crystal dictates the selection rules for Raman-active modes:

- **A**_1_*_g_* **Modes:** These are symmetric stretching modes of the NbO_6_ octahedra, typically strong in Raman spec- tra due to their symmetric breathing motions.
- **B**_1_*_g_* **and B**_2_*_g_* **Modes:** These modes can involve out-of-plane bending or distortion of the octahedra, influ- enced by the layered structure.
- **E***_g_* **Modes:** These doubly degenerate modes involve motions perpendicular to the principal axis, contribut- ing to the complexity of the Raman spectra.

**Expected Raman Features**

In the Raman spectrum of CsBiNb_2_O_7_, we expect the following features:

- **High-Frequency Modes:** These correspond to lattice vibrations and interlayer interactions, influenced by the weak bonding between layers.
- **Mid-Frequency Modes:** Arise from Nb-O bending and stretching within the octahedral layers, reflecting the intrinsic perovskite structure.
- **Low-Frequency Modes:** Associated with in-plane stretching vibrations of the Bi-O and Cs-O bonds.

**Effects of OAM Light**

Using twisted UV light with Laguerre-Gaussian beams, the orbital angular momentum (OAM) can interact with specific symmetry elements:

- **Enhancement of Symmetry-Sensitive Modes:** Certain modes may show enhanced intensity or shifts when excited with twisted light, providing insight into anisotropies or symmetry-breaking effects.
- **Chirality and Anisotropy:** OAM can help detect chiral or anisotropic features that are otherwise difficult to observe with standard Raman techniques.

**Analytical Considerations**

- **Symmetry Analysis:** Understanding the symmetry properties of CsBiNb_2_O_7_ is crucial for interpreting the Raman spectra and identifying OAM-sensitive modes.
- **Mode Assignment:** Detailed analysis and comparison with theoretical models or complementary spectro- scopic techniques might be necessary to assign observed modes accurately.

**Raman Active Modes for CsBiNb**_2_**O**_7_ **under UV OAM Setup**

The use of twisted UV light with Orbital Angular Momentum (OAM) in Raman spectroscopy provides a power- ful method for probing the vibrational modes of materials. Laguerre-Gaussian beams carrying OAM are charac- terized by a helical wavefront and a topological charge *ℓ*, which describes the number of twists in the wavefront.

- **Topological Charge (***ℓ***):** The value of *ℓ* determines the phase structure and intensity distribution of the beam. In this study, we explore cyclic topological charges *ℓ* = 0, *ℓ* = 1, *ℓ* = −1, and returning to *ℓ* = 0.
- **Beam Profile:** For *ℓ* = 0, the beam resembles a standard Gaussian profile. The beam exhibits a doughnut- shaped intensity profile for *ℓ* = ±1, with a phase singularity at the center.

**Symmetry Considerations**

The interaction between the OAM beams and the crystal lattice of CsBiNb_2_O_7_ leads to modified selection rules for Raman-active modes. The symmetry of the crystal, combined with the OAM properties, dictates which modes are enhanced or suppressed.

- **Symmetry of CsBiNb**_2_**O**_7_**:** As a member of the orthogonal space group **P2**_1_**am**, CsBiNb_2_O_7_ has a point group symmetry of **2**_1_**am**.
- **Raman Modes:** The Raman-active modes can be categorized into different symmetry representations: A_1_*_g_*, B_1_*_g_*, B_2_*_g_*, and E*_g_*.
- **OAM-Dependent Enhancement:** The cyclic changes in *ℓ* can selectively enhance certain Raman modes based on their symmetry properties:
  - *ℓ* = 0**:** Primarily excites modes with A_1_*_g_* symmetry due to the Gaussian-like intensity profile, maximiz- ing symmetric stretching and breathing modes.
  - *ℓ* = 1**:** Enhances modes with anisotropic character, such as E*_g_* modes, which involve motions perpen- dicular to the principal axis, due to the doughnut-shaped beam intensity.
  - *ℓ* = −1**:** Similar to *ℓ* = 1, but may introduce subtle changes in mode intensities due to the opposite helicity, providing insights into chirality and anisotropy.
  - **Return to** *ℓ* = 0**:** Serves as a control measurement to confirm the cyclic behavior and assess potential structural changes induced by OAM cycling.

**Data Collection and Analysis**

Data is collected at each topological charge value (*ℓ* = 0, *ℓ* = 1, *ℓ* = −1, and *ℓ* = 0) to understand how the OAM influences Raman scattering.

- **Experimental Setup:** A UV laser capable of generating Laguerre-Gaussian beams is used, with the beam polarization aligned to maximize interaction with specific vibrational modes (Figure S30). In order to un- derstand the stability of the system under UV illumination we performed timed Raman scans on a similarly grown flake as shown in Figure S34.
- **Raman Spectrum Acquisition:** At each *ℓ* value, Raman spectra are recorded to capture the intensity and frequency of vibrational modes. Comparison of these spectra reveals the influence of OAM on mode selec- tion.

## Mode Assignment and Symmetry Analysis:

- - Identify peaks corresponding to Raman-active modes and assign them based on theoretical models, previously reported values^[[19]](#endnote-19)^, and symmetry considerations.
  - Analyze the intensity variations and frequency shifts induced by different *ℓ* values to deduce informa- tion about lattice dynamics and symmetry breaking.
- **Visualization:** Plot the Raman spectra at each *ℓ* value, highlighting the differences in peak intensities and positions. Use color coding or markers to indicate symmetry-enhanced modes.

**Conclusion**

The cyclic variation of topological charges in the OAM setup provides a novel approach to probing the vibra- tional properties of CsBiNb_2_O_7_. By systematically analyzing the Raman spectra collected at each *l* value, we can gain deeper insights into the material’s symmetry, lattice dynamics, and potential for symmetry-related ap- plications(Table S4) . This approach not only enhances our understanding of CsBiNb_2_O_7_ but also demonstrates the broader applicability of OAM-enhanced Raman spectroscopy in material science. The schematic represen- tation of the Raman Set-up is shown in Figure S29. We use a witec alpha 300R with a free beam coupler to per-

form an insitu Raman spectroscopy. A Zeiss 100x objective was used to focus the OAM light as well as the green laser on to the sample surface. The spectroscope grating of 1800 mm/gr was used to collect the scans. To under- stand the changes the CBNO nanoflake undergoes under twisted light and confirm the structure of CBNO Flakes we perform reference scans on them as shown in Figure S31. We also track the changes in the Raman intensity as a function of applied topological charge, which provides us with valuable information about the strength and activity of the phonon modes as shown in Figure S33 As mentioned in Section S4 earlier, we also compare the Raman spectra computed for Ref unit cell as well as the the unit cell under strain equivalent to strain from *ℓ* = 1

which agrees with our experimental evidence as shown in Figure S32

## Section S19: Analysis of Power and Time-dependent Raman Spectrum

The plots show the peak position of the Bi–O (in-plane), Bi–O (out-of-plane), and Nb–O (in-plane) (See Figures S 34, 35, 36, 37) vibrational modes as a function of increasing power density (*kW/cm*^2^). Several important observations support the role of non-linear mechanisms:

## Non-Monotonic Shifts:

We notice that as the power is increased, the vibrational modes do not exhibit a simple linear or monotonic shift that would be expected from purely thermal or single-photon effects. Instead, the data reveal non-monotonic and sometimes abrupt changes in the Raman peak positions, which is consistent with threshold-like behavior often associated with multi-photon absorption.

## Enhanced Lattice Distortions:

The distinct responses of the Bi–O (in-plane), Bi–O (out-of-plane), and Nb–O (in-plane) modes highlight se- lective bond distortions under high-intensity twisted-light illumination. This selectivity implies a bond-specific non-linear response, rather than uniform thermal expansion across all modes.

## Corroboration with Twisted-Light Effects:

These power-dependent shifts align with our broader findings—such as hysteresis-like strain loops observed in Bragg Coherent Diffractive Imaging (BCDI) data—indicating that local lattice distortions scale with both in- tensity and the orbital angular momentum of the incident beam. This suggests a synergistic interplay between multi-photon excitation and the unique spatial phase structure of twisted light.

## Distinction from Simple Heating:

To differentiate these effects from trivial heating, we compared the power-dependent Raman data to temperature- dependent Raman measurements (see Figure Q5 vs Figure Q3). The thermal shifts observed at elevated tempera- tures are notably smaller and exhibit a smooth, monotonic trend, whereas the non-linear, power-dependent shifts under twisted light cannot be explained solely by heating.

These power/intensity-dependent Raman measurements thus provide experimental evidence supporting the role of multi-photon absorption and other non-linear processes in generating strain heterogeneity. While we acknowl-

edge that additional time-resolved or microscopic imaging techniques could offer further confirmation, the non- linear power dependence observed here, combined with the spatially resolved BCDI data, presents a compelling case that these processes are indeed operative in CBNO under twisted-light excitation. However, time resolved measurements are warranted to delineate them.


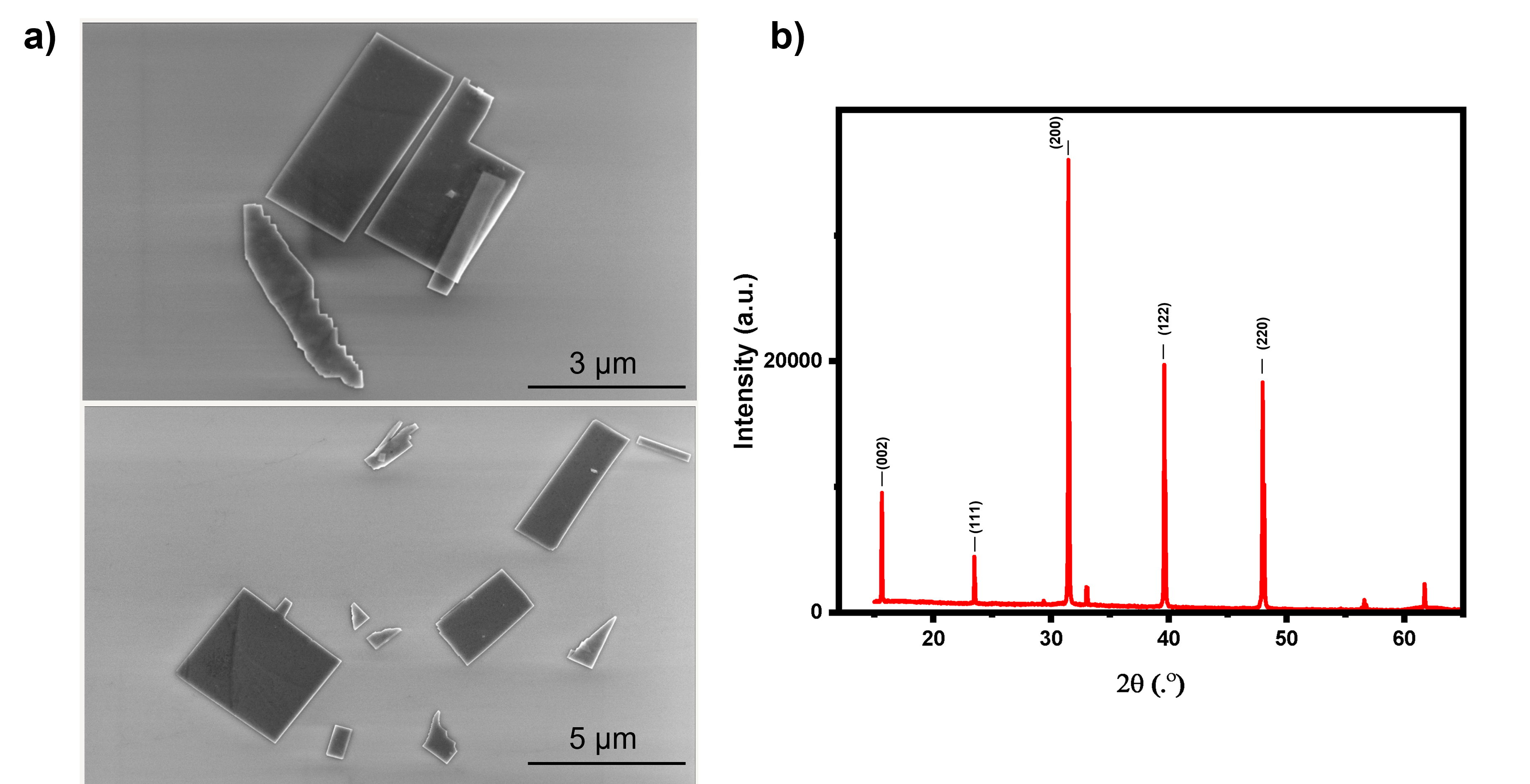


Figure S 1: **a:** SEM of the *CsBiNb*_2_*O*_7_ (CBNO). **b:** Powder XRD of CBNO particles.


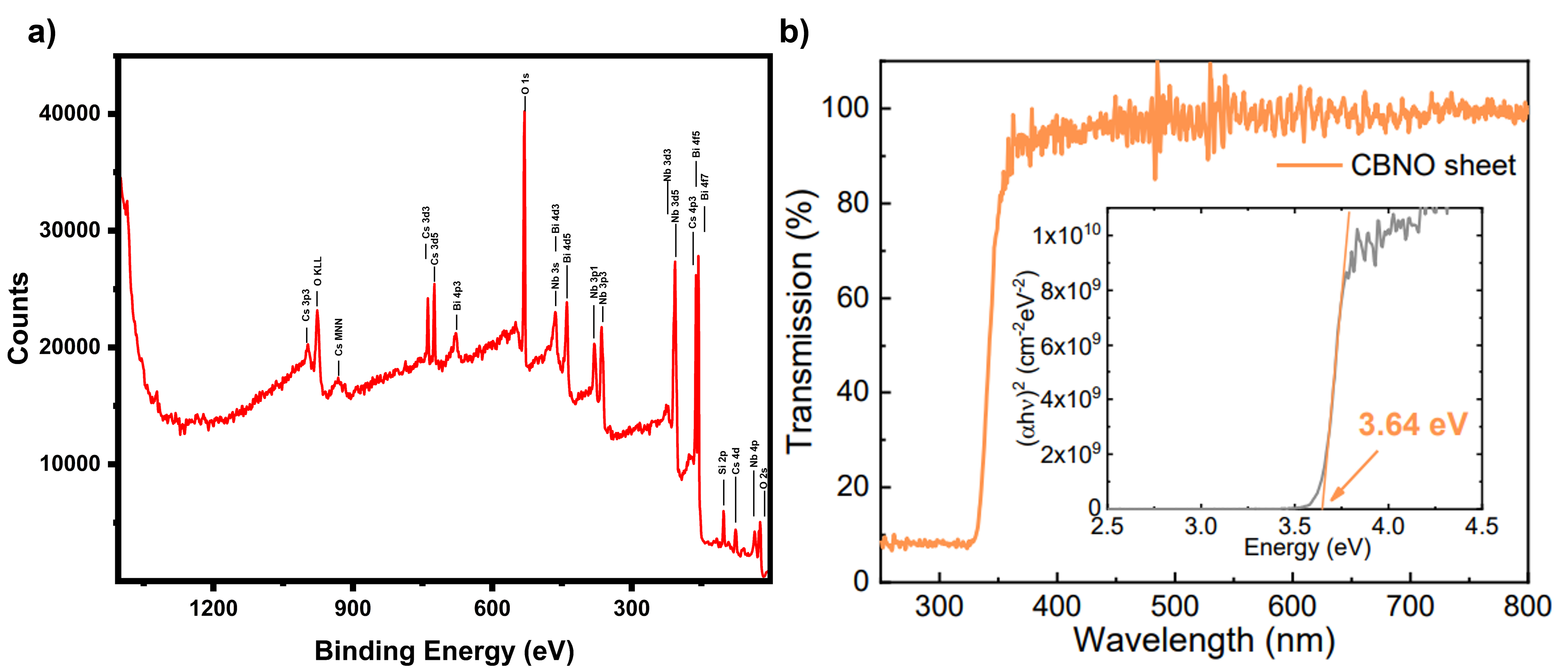


Figure S 2: **a:** XPS of the CBNO nanoparticles. **b:** Transmission Spectra of CBNO adapted from Jiang et al .


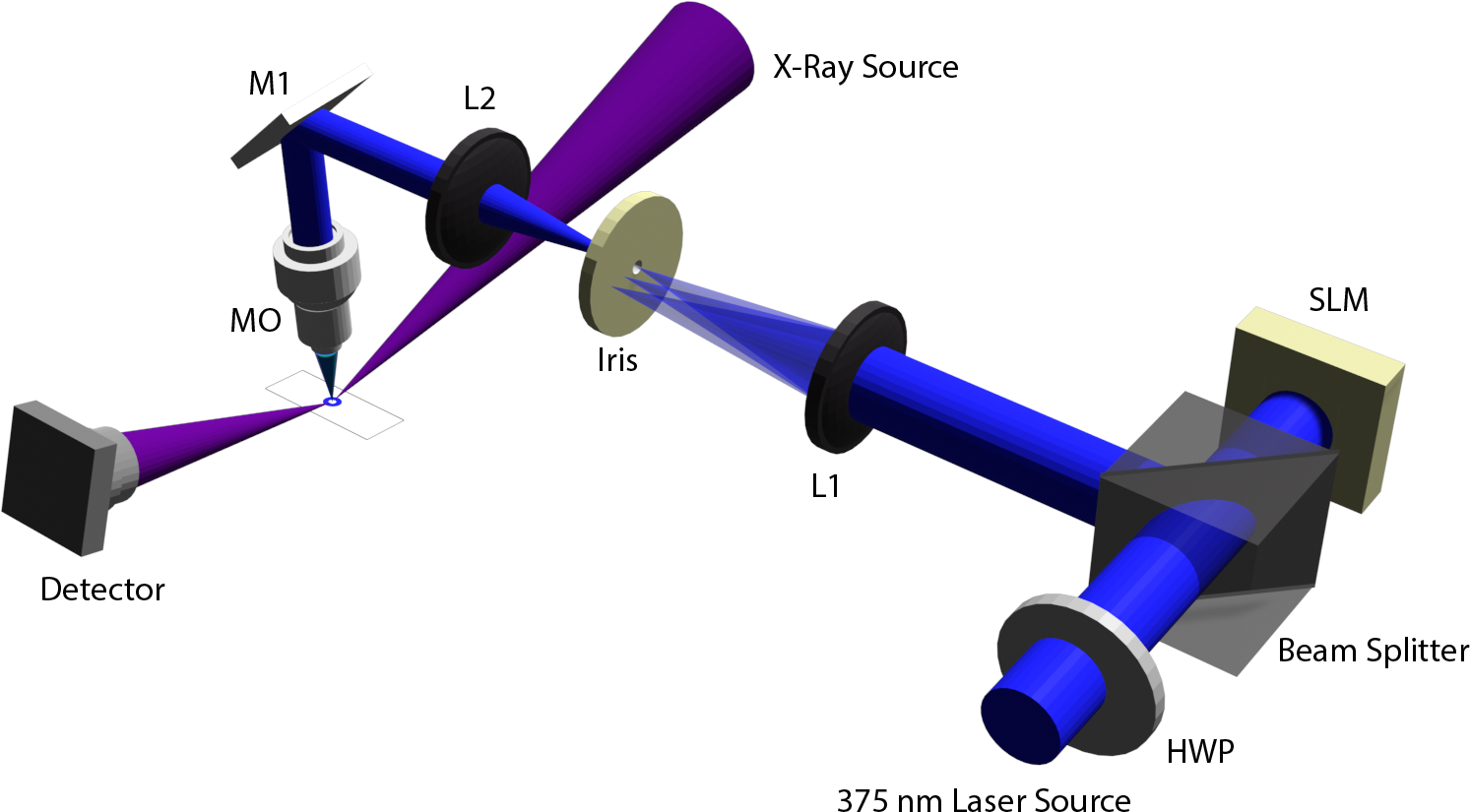


Figure S 3: Schematic of the in-operando BCDI set-up for producing the twisted light.


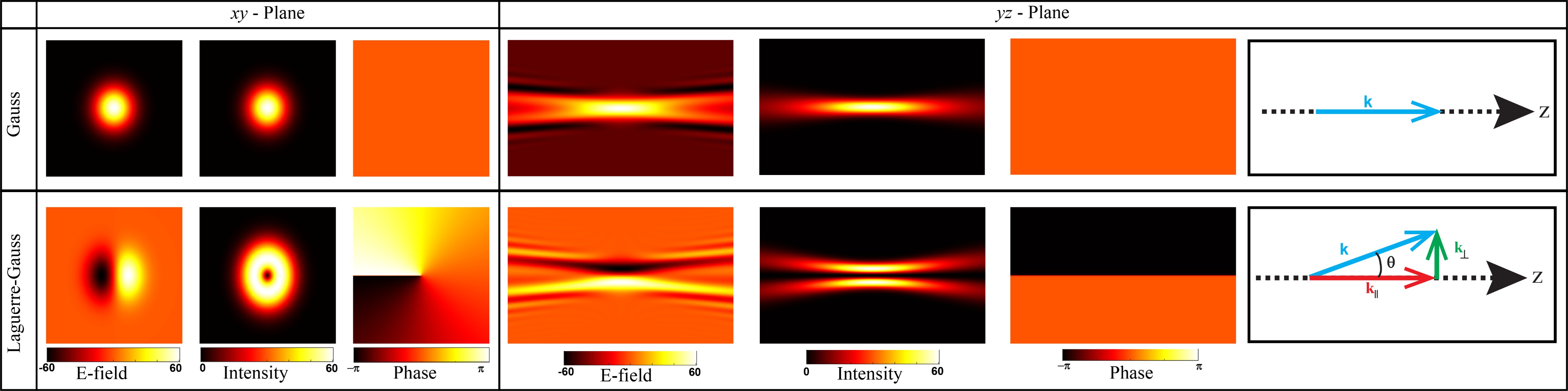


Figure S 4: A model showing the twisted light in various directions. The focus is on the Intensity, Electric Field, and Phase of the beam.


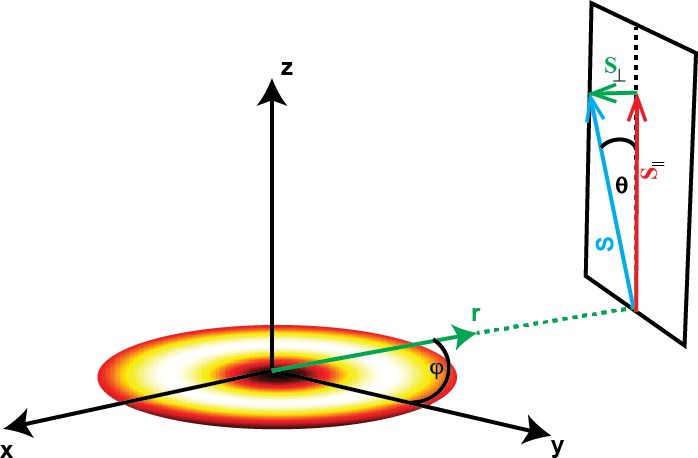


Figure S 5: A schematic of the Poynting Vector along the propagation direction.

# a b


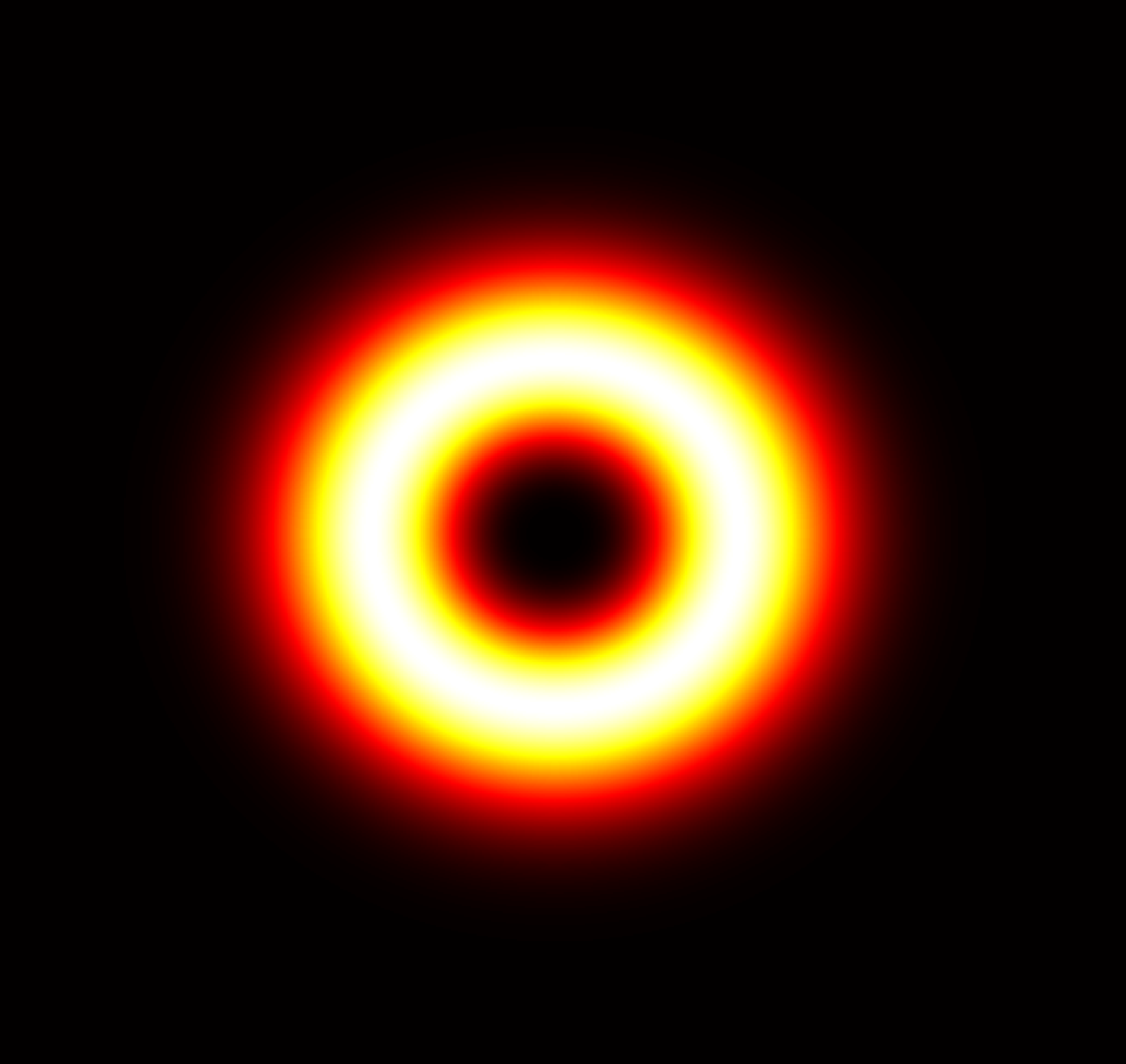

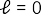

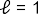

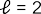

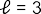

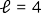

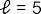


10-9

1

0.8

Intensity (W/m2)

0.6

0.4

0.2

# c

3.5

0

-6 -4 -2 0 2 4 6

Position r (m)
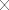
10-6

3


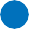

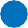

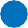

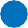

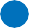

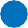

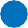

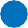

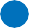

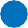

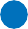


2.5

Electric Flux (V*nm)

2

1.5

1

0.5

-5 -4 -3 -2 -1 0 1 2 3 4 5

Topological Charge

Figure S 6: Characterization of LG beams as a function of topological charge from *ℓ* = 0 to *ℓ* = 5 and their conjugate counterparts. (a): Intensity profile of an LG beam with arbitrary *ℓ*. (b) Cross-sectional intensity line plots along the diameter (indicated by the blue dashed line) as for different topological charge *ℓ*. (c) Calculated Electric flux of each LG beam as a function of topological charge *ℓ*.


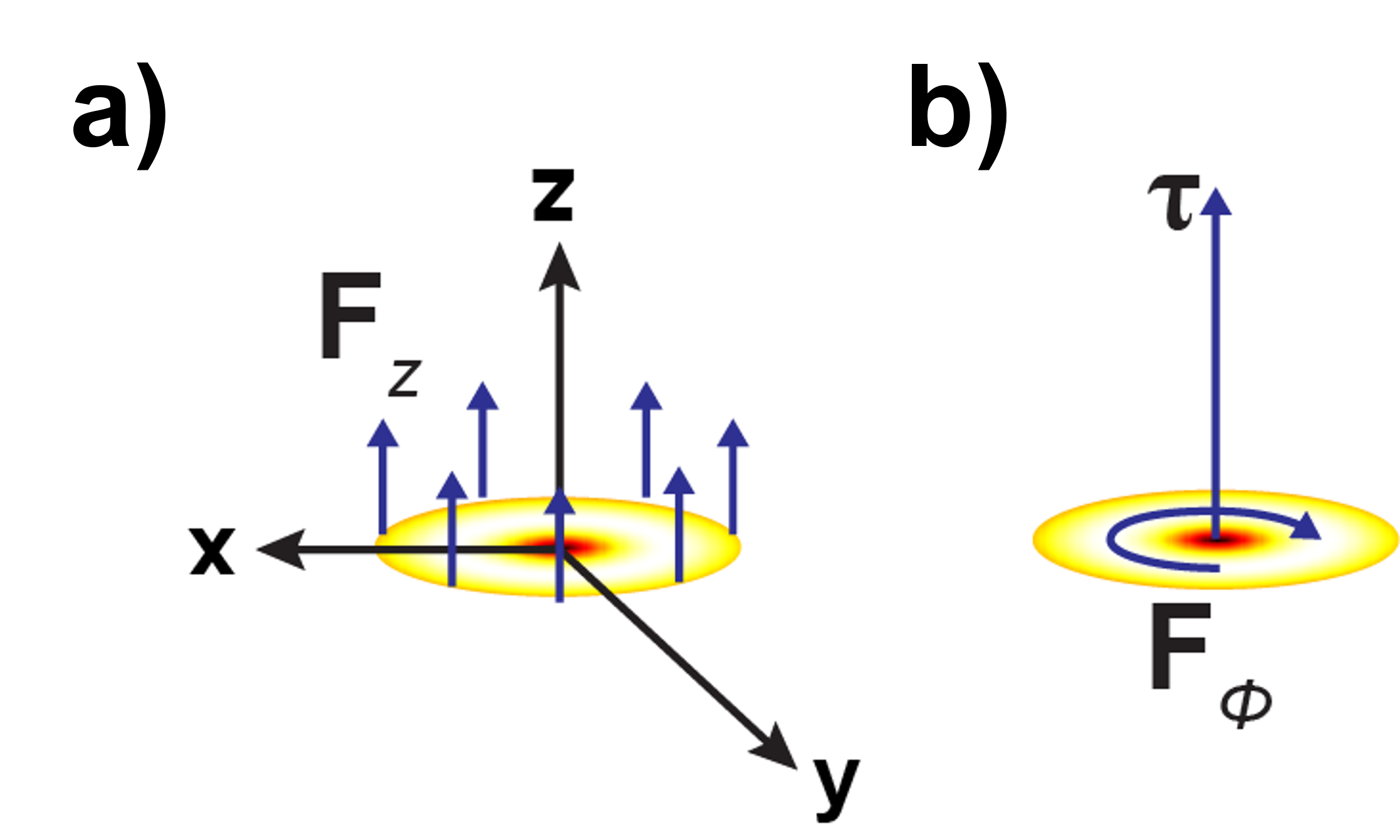


Figure S 7: **a:**Schematic of the Force experienced by the particle from the beam. **b:** Schematic of the torque on the particle from the beam.


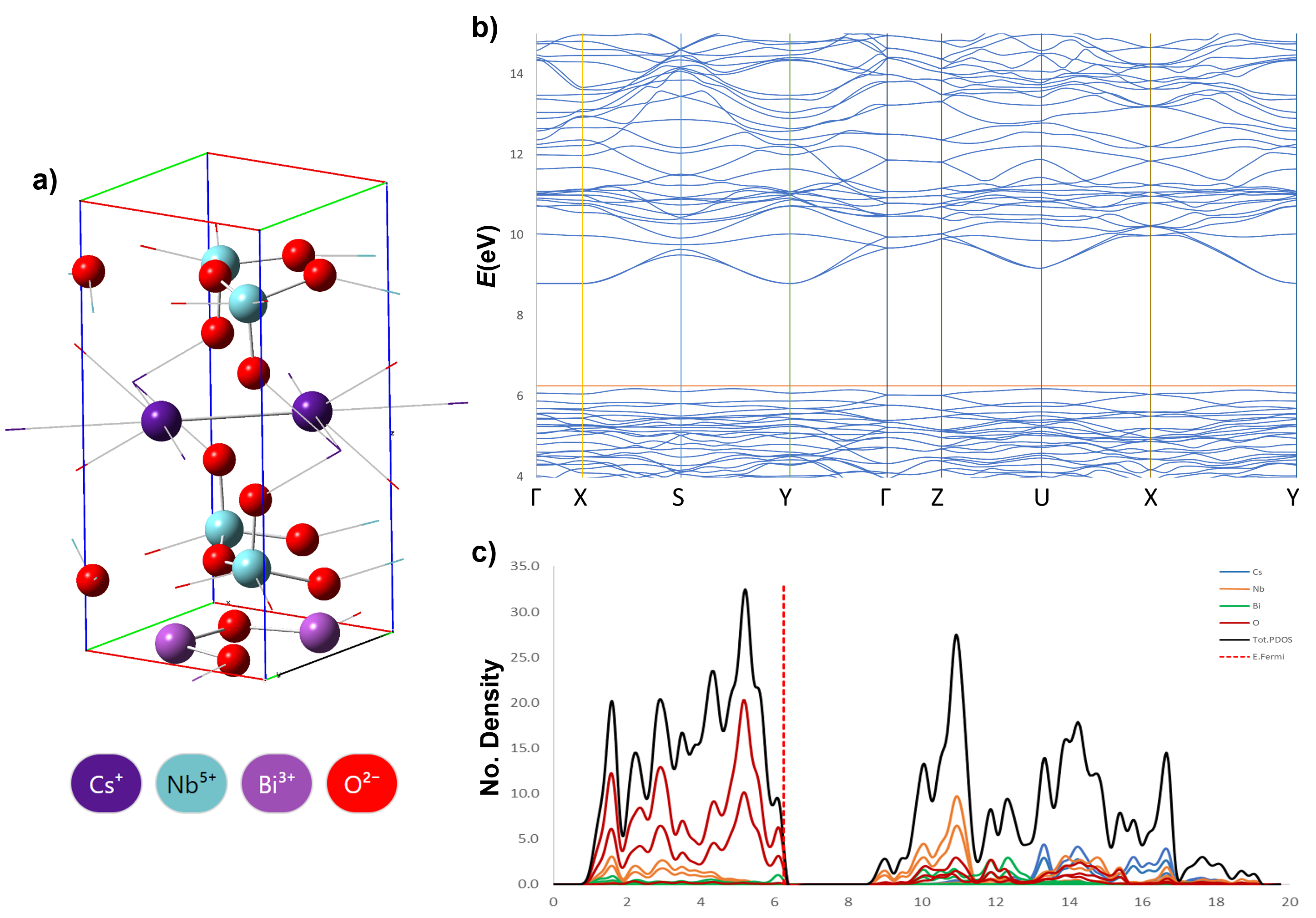


Figure S 8: **a:**CBNO Unit Cell **b:**CBNO Calculated band structure **c:** Density of States for a CBNO unit cell


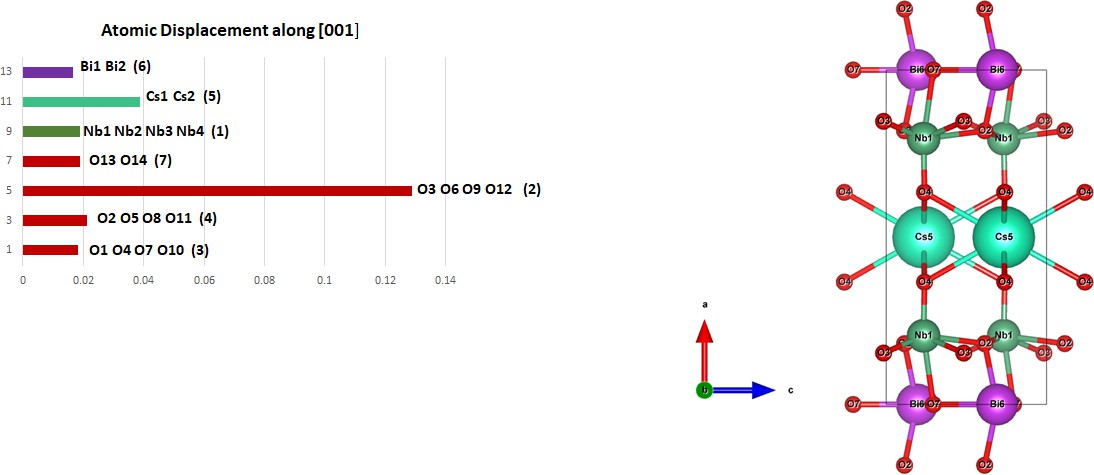


Figure S 9: **A**tomic displacement along [001] axis.

| Topological Charge | Torque(N·*µ*m) | Power (*µ*W) | Electric Flux (V·nm) |
| --- | --- | --- | --- |
| 0 | 0 | 13.5 | 0.92 |
| 1 | 0.025 | 34.0 | 1.83 |
| -1 | 0.025 | 34.1 | 1.83 |

Table S 1: Twisted Light Torque and Electric Fields


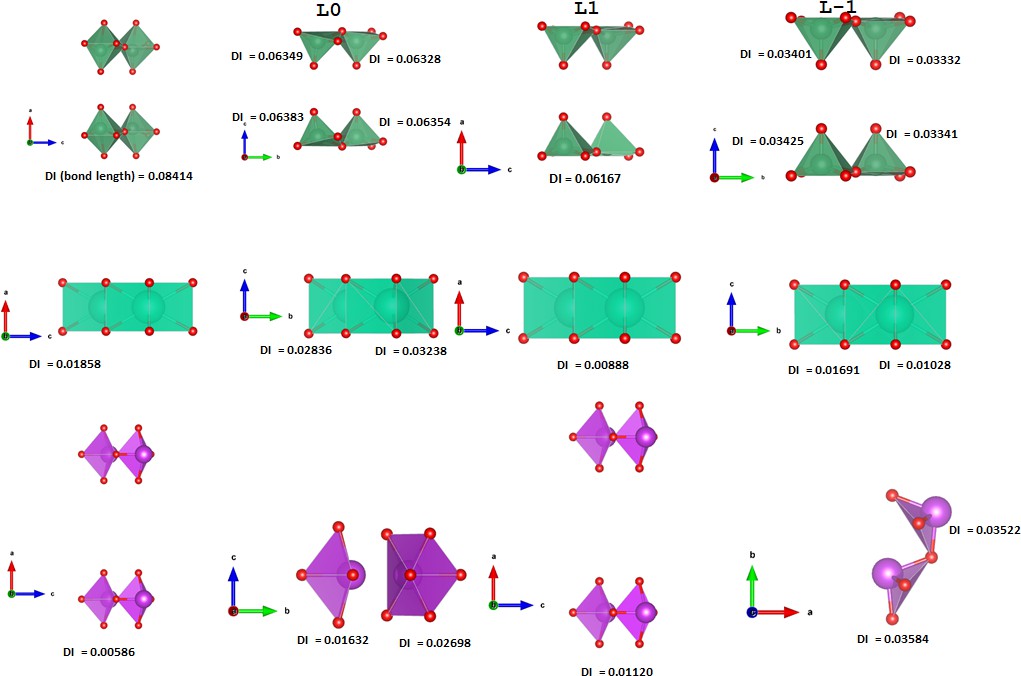


Figure S 10: **C**alculation of the distortion index (DI) for bond lengths: The higher value indicates a greater degree of bond length variation, leading to more significant structural distortions, which is more favorable for lowering the symmetry of the crystal. These distortions are often necessary to break the centrosymmetry of the crystal, thereby enabling the development of spontaneous polariza- tion characteristic of ferroelectric materials. Given bond lengths *l_i_* and the average bond length *l*¯, the distorsion index (DI) is calculated

¯

as:[*DI* = ^1^ ∑*^n^*

*n*

*i*=1

1*l_i_* − *l*1]

| Topological Charge | *ε_zz_*(%) | *δu_x_* (%) | *δu_y_* (%) |
| --- | --- | --- | --- |
| 0 | 0.70 | 0.68 | 0.58 |
| 1 | 0.75 | 0.76 | 0.76 |
| -1 | 0.41 | 0.71 | 0.64 |
| 0’ | 0.71 | 0.65 | 0.72 |

Table S 2: Compressive strains

| Topological Charge | *ε_zz_*(%) | *δu_x_* (%) | *δu_y_* (%) |
| --- | --- | --- | --- |
| 0 | 0.74 | 0.63 | 0.41 |
| 1 | 0.78 | 0.78 | 0.78 |
| -1 | 0.35 | 0.36 | 0.71 |
| 0’ | 0.73 | 0.74 | 0.68 |

Table S 3: Tensile strains

| **Scan** | **In-Plane Bi-O** | **Out-of-Plane Bi-O** | **In-Plane Nb-O** |
| --- | --- | --- | --- |
| **Ref** | 194.87 | 385.38 | 591.38 |
| **0** | 193.11 | 384.86 | 590.61 |
| **1** | 194.19 | 385.26 | 592.2 |
| **-1** | 194.6 | 386.16 | 592.64 |
| **0’** | 193.36 | 385.75 | 591.47 |

Table S 4: Raman Peak Shift due to Twisted Light


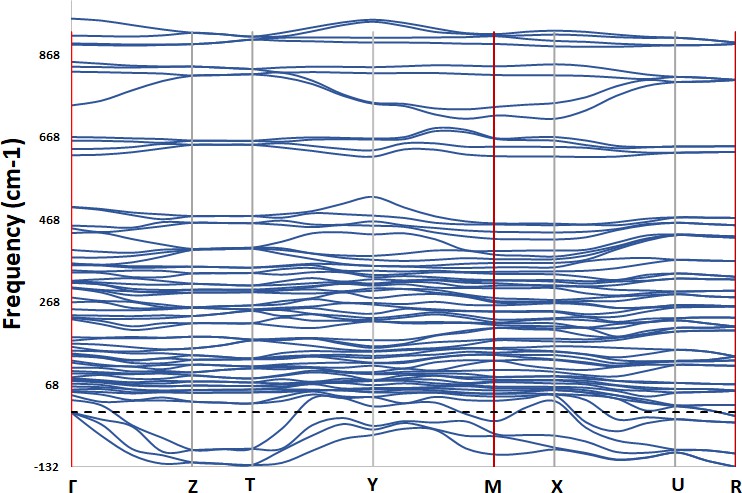


Figure S 11: **P**honon Dispersion of CsBiNb2O7. This figure presents the phonon dispersion curves of the CsBiNb2O7 material, cal- culated using density functional perturbation theory (DFPT). The horizontal axis represents the wave vector along high-symmetry directions in the Brillouin zone, while the vertical axis denotes the phonon frequencies in inverse centimeters (cm^−1^). The presence of acoustic phonon branches, which exhibit linear behavior near the Γ point, is noted. Optical modes at the Γ, M, and R points (0, 0,

0; 1/2, 1/2, 0; 1/2, 1/2, 1/2) with higher frequencies involve vibrations of atoms that lead to changes in the polarization of the crystal lattice. Notable gaps between the acoustic and optical phonon branches indicate regions of phonon band gaps. Additionally, negative frequencies are observed at all points along the edges of the Brillouin zone, indicating potential dynamic instability within the crystal structure due to the softening of the optical mode. The primary order parameter driving the transition is not directly related to strain, but to a soft mode at a specific point in the Brillouin zone. This provides information on the nature and mechanism of the improper ferroelastic phase transition.


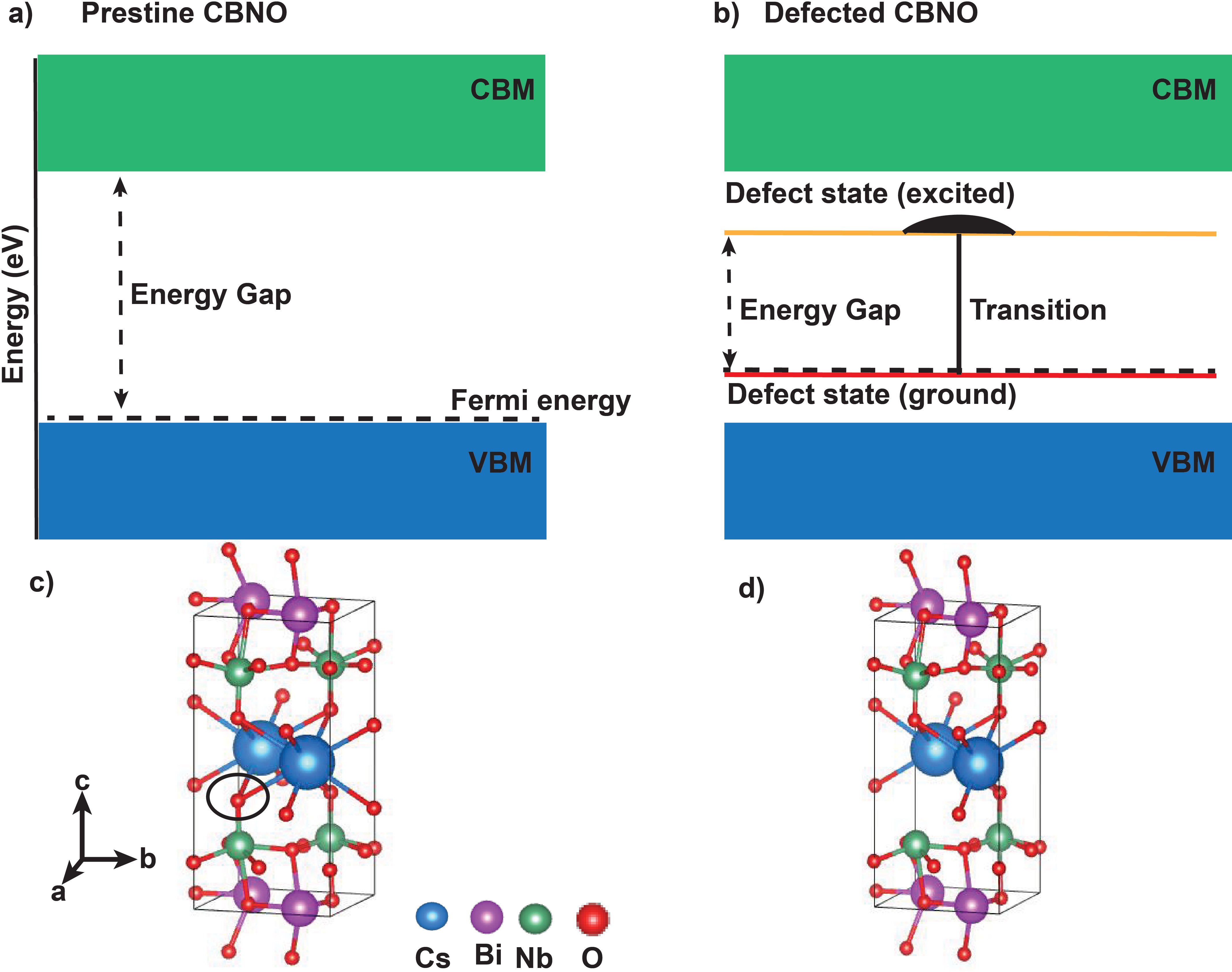


Figure S 12: **Electronic structure of CBNO (Cesium Bismuth Niobium Oxide) in pristine and defected states.** *(a)* For pristine CBNO, the valence band maximum (VBM) and conduction band minimum (CBM) are separated by a bandgap of approximately 3.2 eV. The Fermi energy lies within the gap, indicating an insulating state. *(b)* For defected CBNO with an oxygen vacancy near the Nb-O bond, localized defect states form within the bandgap. The ground and excited defect states are depicted, along with possible sub-gap optical transitions. A significant reduction in the effective bandgap is observed due to the defect levels, enabling transitions induced by light at 375 nm (3.3 eV). Structural representation of CBNO showing the atomic arrangement of Cs (blue), Bi (purple), Nb (green), and O (red) atoms. The *(c)* highlights the pristine structure, while *(d)* illustrates the defect location (encircled region).


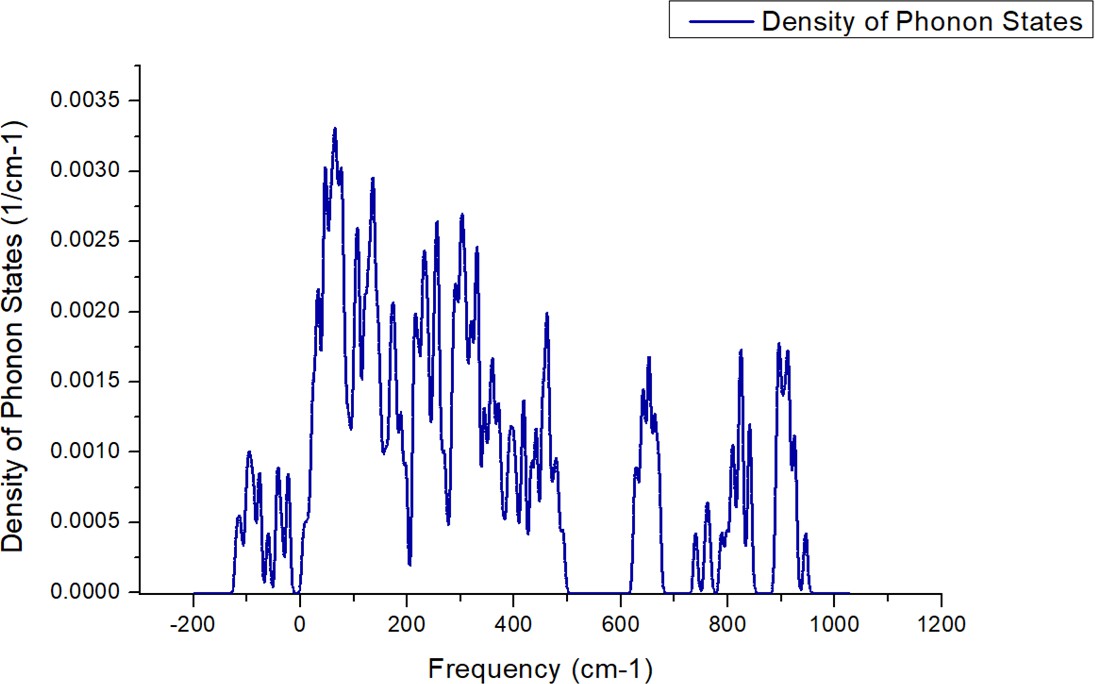


Figure S 13: **D**ensity of Phonon States (DOS) of CsBiNb2O7. This figure illustrates the density of phonon states (DOS) for the Cs- BiNb2O7, calculated using density functional perturbation theory (DFPT). The horizontal axis represents the phonon frequencies in cm^−1^, while the vertical axis denotes the density of states (DOS) in (1/ cm^−1^). Key features of the DOS include: Peaks at specific frequencies corresponding to the vibrational modes of the atoms within the crystal lattice. Distinct peaks at higher frequencies, which are primarily associated with the optical phonon modes. Lower frequency regions dominated by contributions from acoustic phonon

modes. Gaps in the DOS, indicating phonon band gaps that separate different vibrational modes. The DOS provides valuable insights into the vibrational properties of CsBiNb2O7 and can be used to predict phase stability (hard polar mode)and potential phase tran- sitions.The presence of soft modes (negative or very low-frequency modes) indicate potential phase instability, suggesting that the crystal structure may undergo a phase transition under certain conditions. By comparing the DOS of different structural modifications, one can identify which structures are dynamically stable (absence of soft modes) and which are prone to phase transitions. The dis- tribution and characteristics of the DOS peaks provide information on how atomic vibrations contribute to the overall stability of the structure, helping to understand which structural modifications are more thermodynamically favorable.

**a) b) c)**

**ℓ = 0 ℓ = -1 ℓ = 1**

**Intensity (a.u.)**

**Phase(rad)**


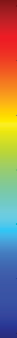

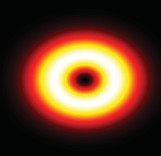

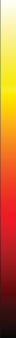

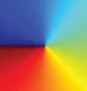

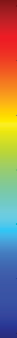

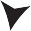

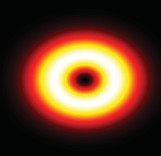

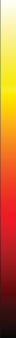

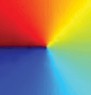

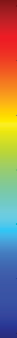

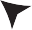


**
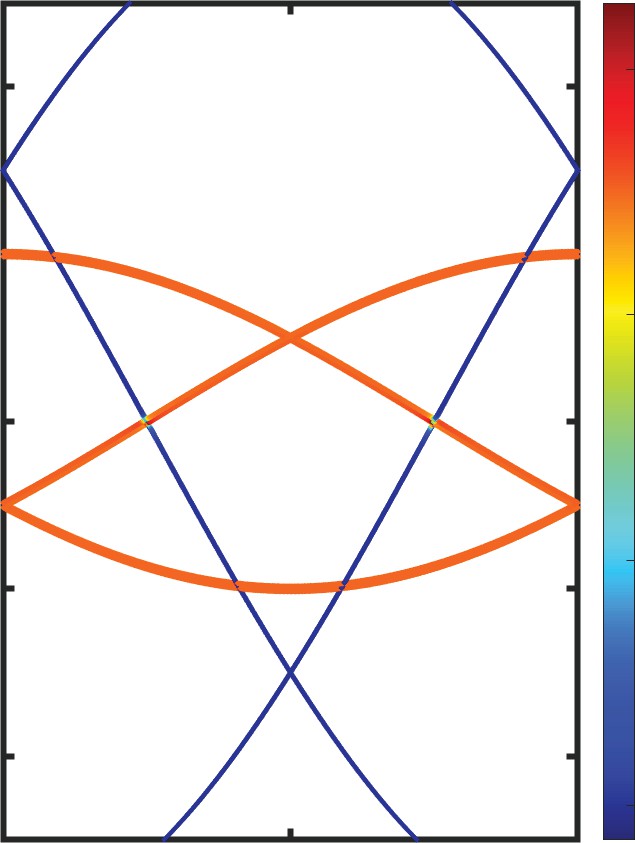
0.6**


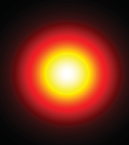

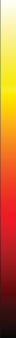


**2**

**0.8**

**
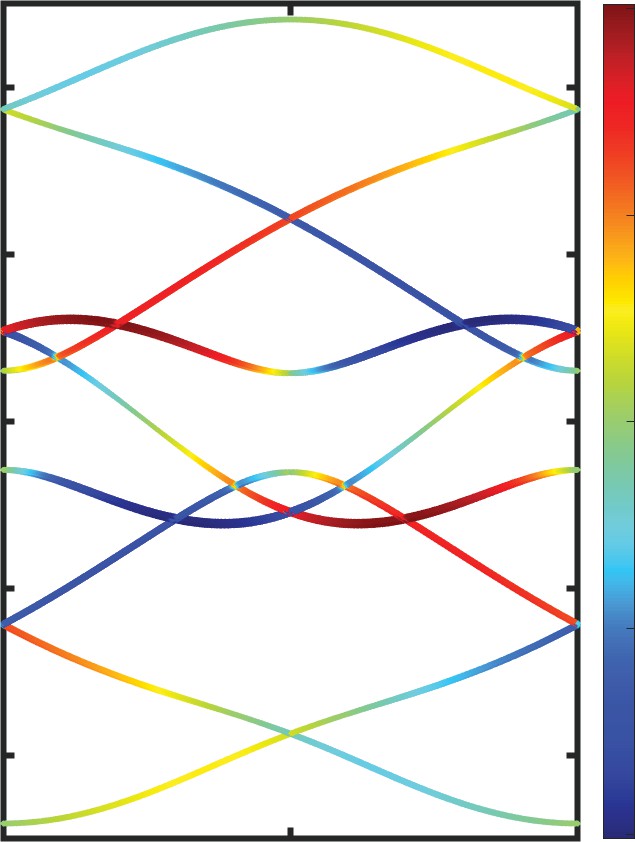
2**

**0.8**

**
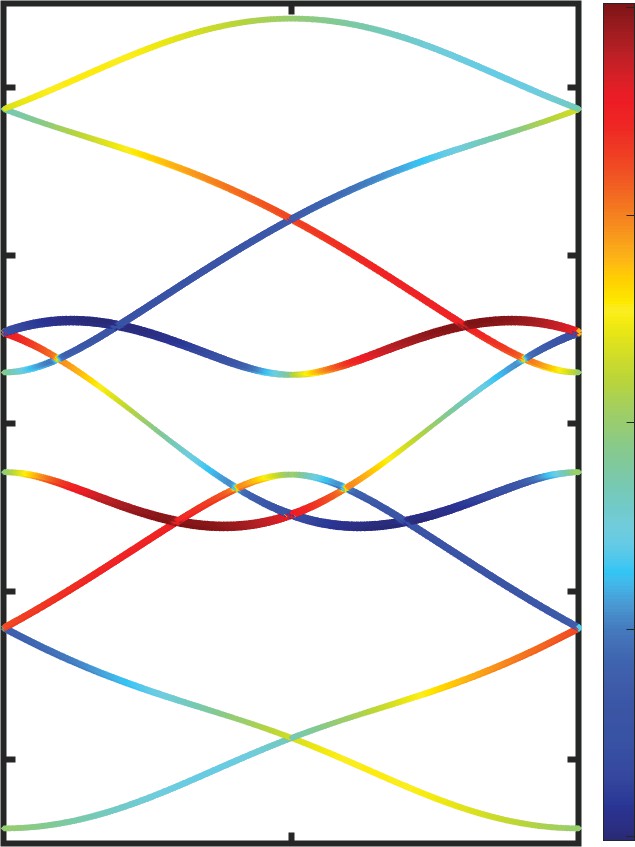
2**

**0 0.3 0 0 0 0**

***L*z(a.u.)**

**Energy (a.u.)**

**-2 -2 -2**

| **-Z** | Γ | **Z 0** | **-Z** | Γ | **Z** | **-0.8 -Z** | Γ | **Z -0.8** |
| --- | --- | --- | --- | --- | --- | --- | --- | --- |
|  | **kZ** |  |  | **kZ** |  |  | **kZ** |  |

Figure S 14: Tight Binding Model in CBNO unit cell for Bi-O-Nb atomic positions in the reciprocal space.


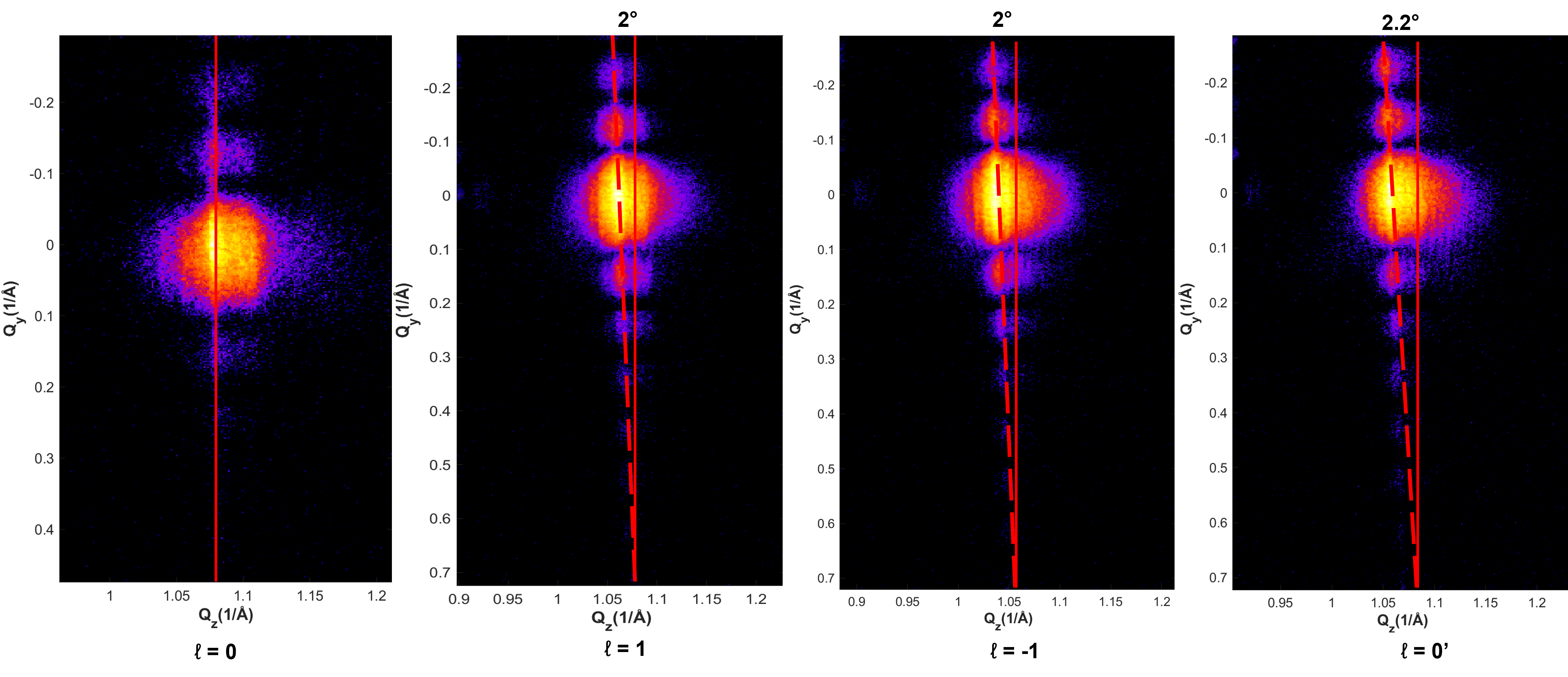


Figure S 15: Angular changes observed in the collected diffraction patterns.


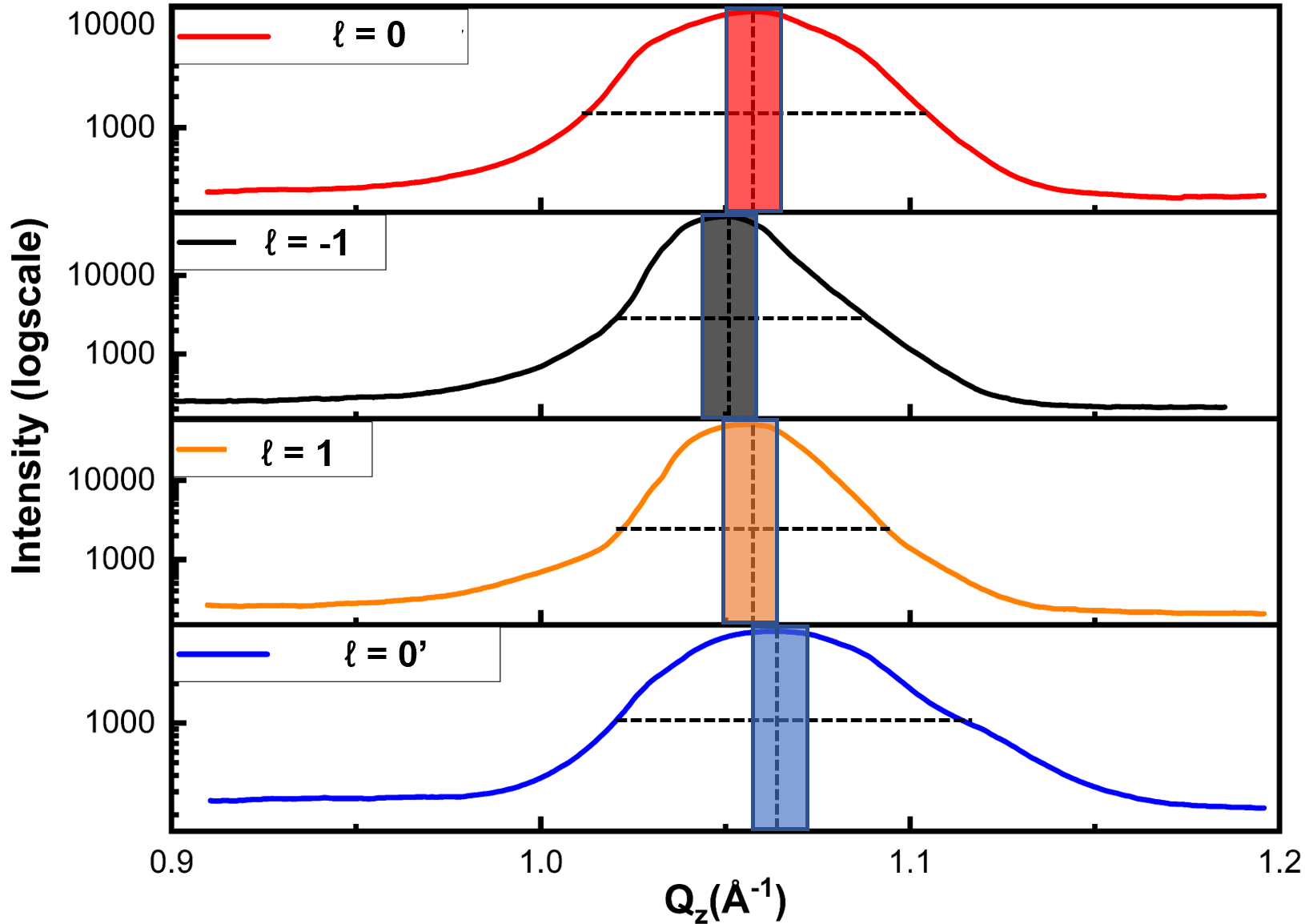


Figure S 16: Line plots showing the variation in the Bragg peak along the *Q_z_* direction. This shows presence of a in-homogeneous strain in the particle on illumination with *ℓ* = 0 which is observed to be homogenized during application of *ℓ* = 1 & *ℓ* = −1


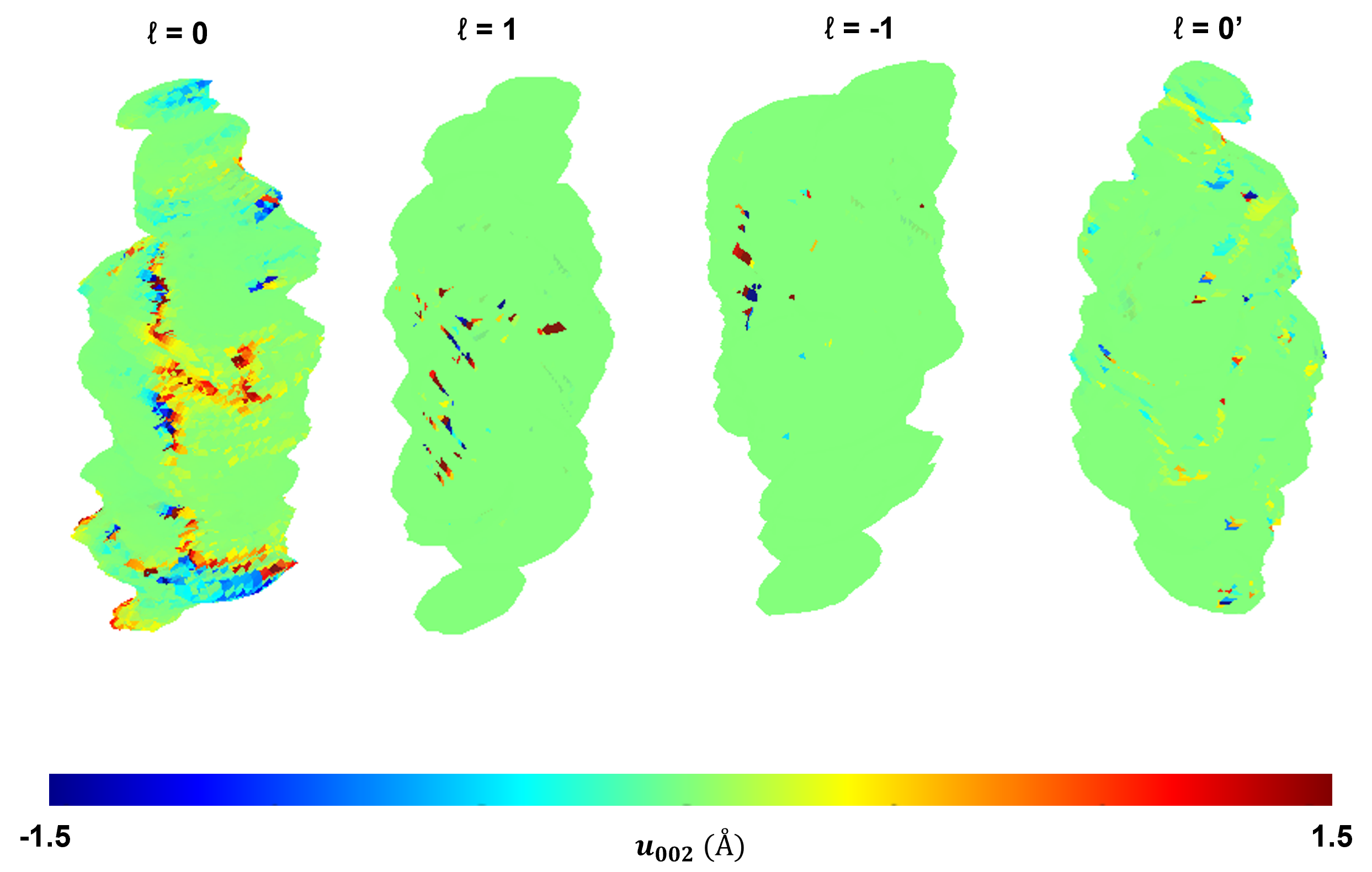


Figure S 17: Isosurfaces of the Bragg Electronic Density for CBNO particle when illuminated with different topolofies


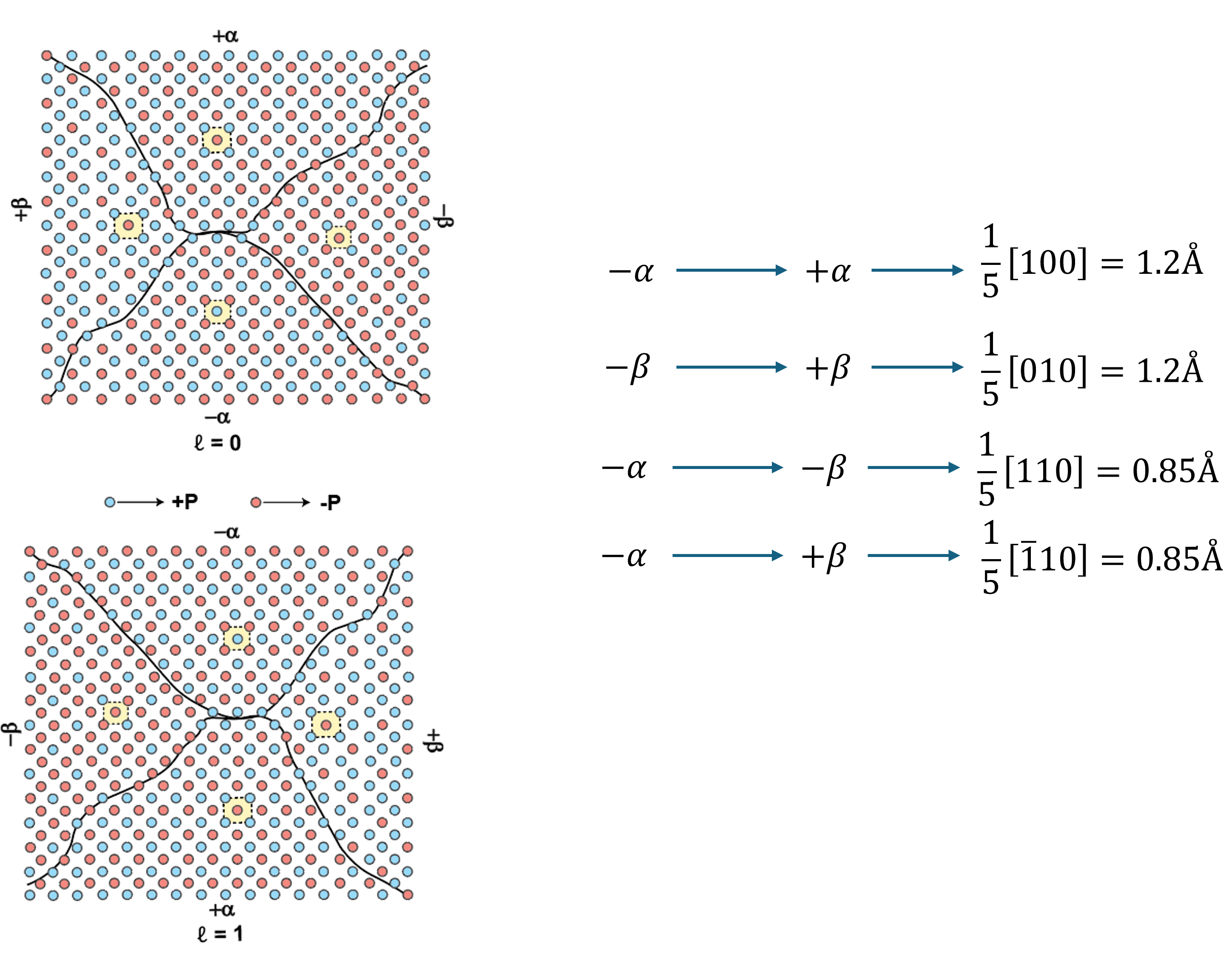


Figure S 18: CBNO Ferroelectric Domain Transformations under Twisted Light.


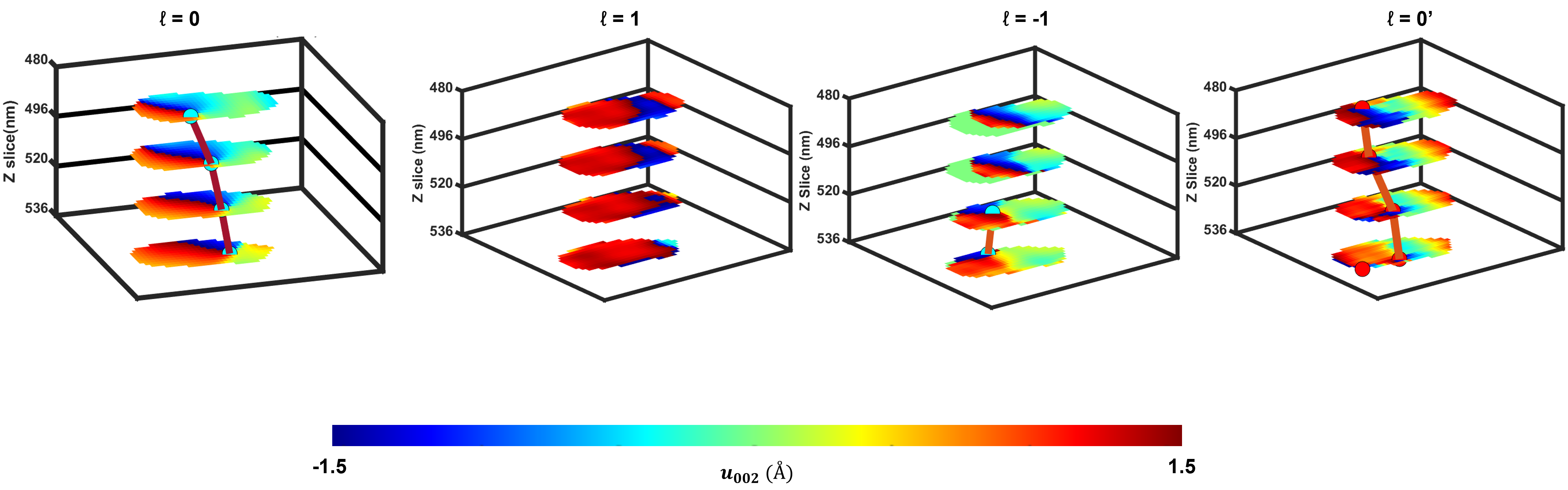


Figure S 19: Tracking Vortices as a function of Applied Topological charge.


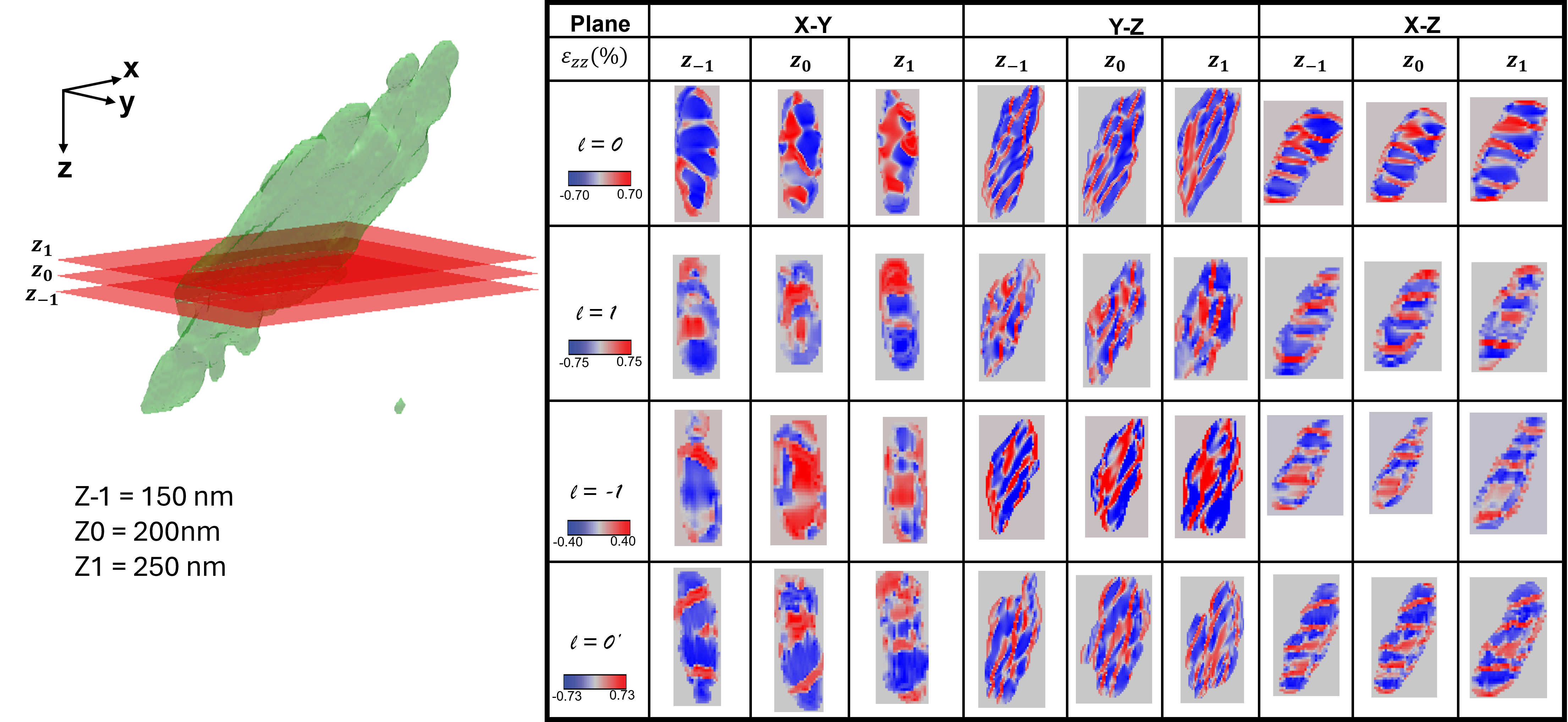


Figure S 20: Strain as a function of Applied Topological charge and electric Field at different slices.A comprehensive comparison can be done on the topological polar structures and their signatures in the strain maps in three dimensions. We observe a reversible control of the said polar topologies with a TL-induced vortex field.


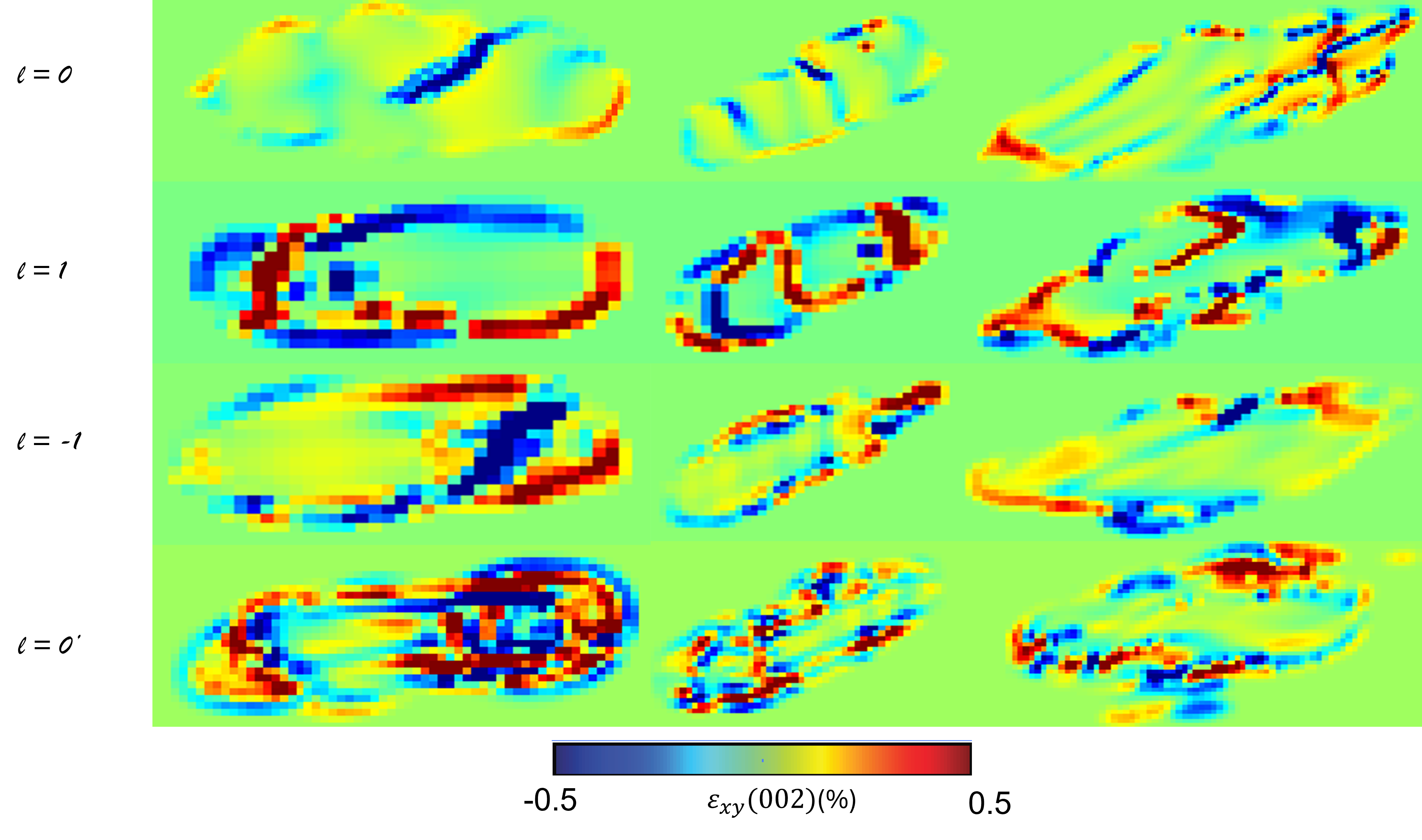


Figure S 21: Shear Strain as a function of applied topological charge from the twisted light. On comparing the shear strain observed at *ℓ* = 0 & *ℓ* = 1, we can conclude that the vortex core in *ℓ* = 1 is shifted or displaced due to the application of an inhomogeneous field. Further, on application of *ℓ* = −1 we observe similar signatures of the vortex returning but partially, this is due to the negative direction of the inhomogeneous electric field.


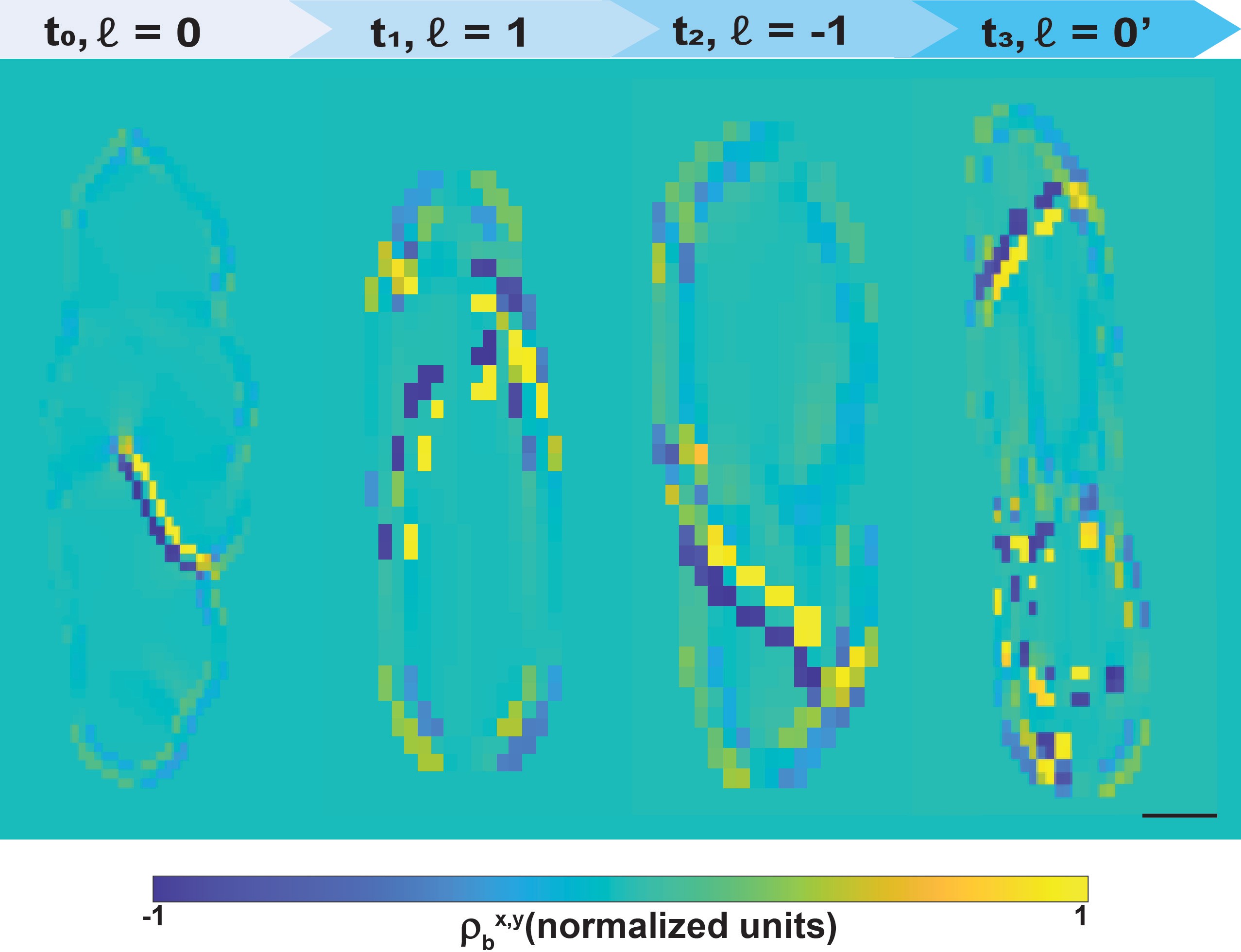


Figure S 22: Central Slice through the [001] or z-direction showing the bound charge density in the nanoflake as a function of the ap- plied topological charge. As observed in the figure, *ℓ* = 0 & *ℓ* = 0^′^ which have an homogeneous electric field harbour a vortex and show boundcharges being present at the vortex core. However, with an inhomogeneous electric field, the *ℓ* = 1 show bound charges near the domain wall region in the central slice of the nanoflake.


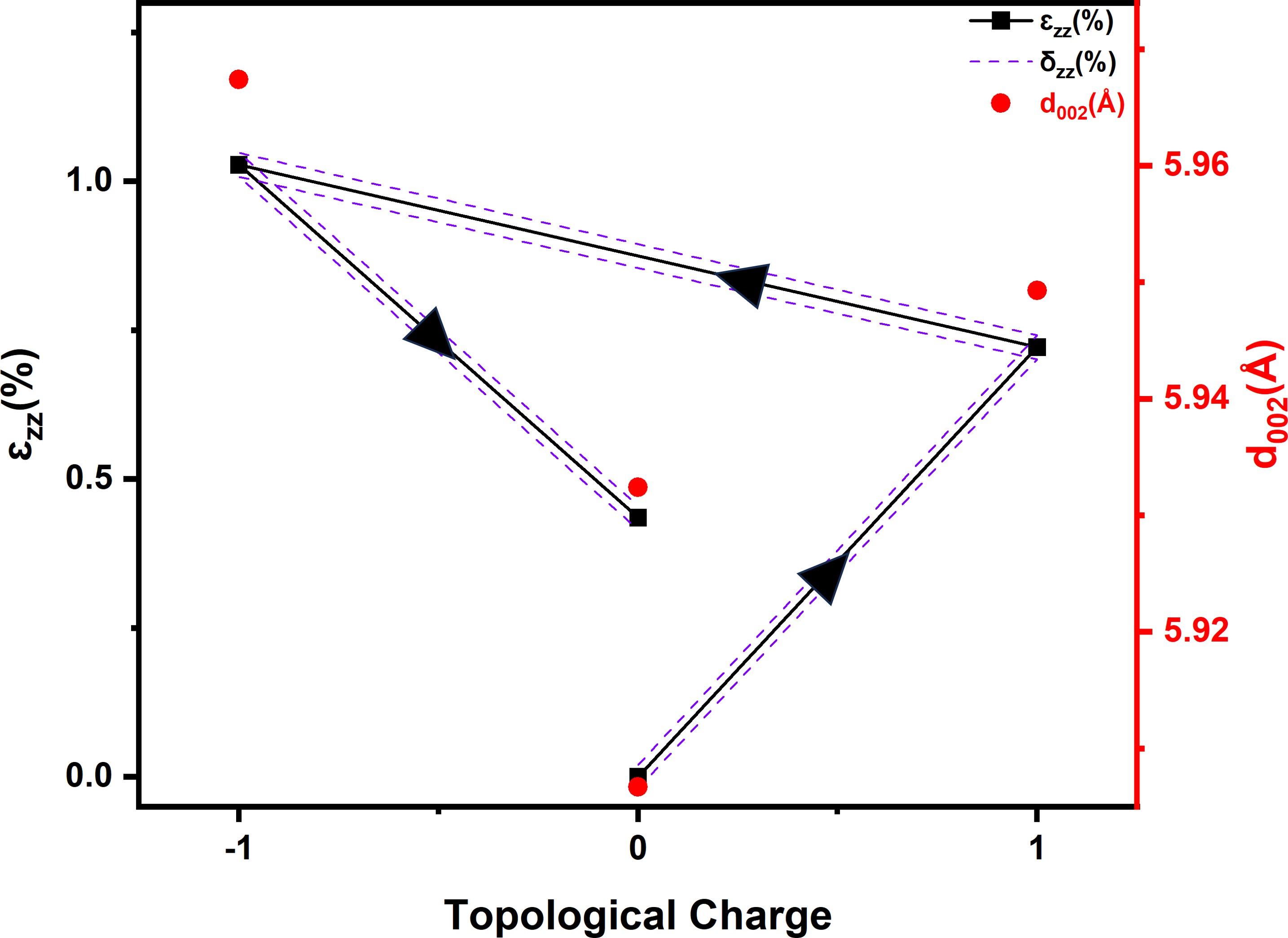


Figure S 23: Strain as a function of Applied Topological charge

XY Plane - Slice 97


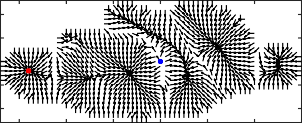

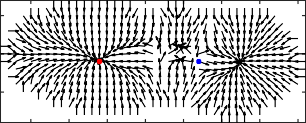

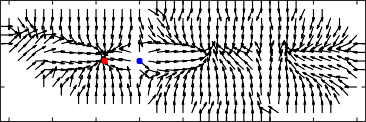

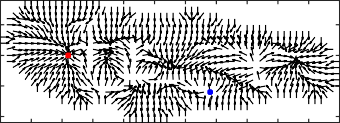


XZ Plane - Slice 95


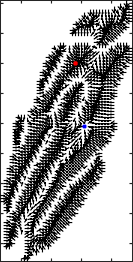

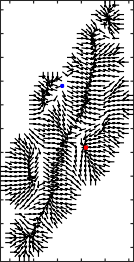

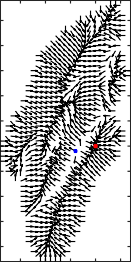

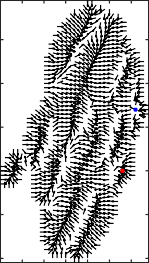


YZ Plane - Slice 70


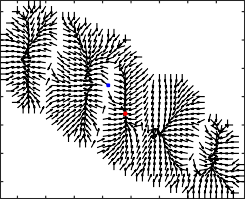

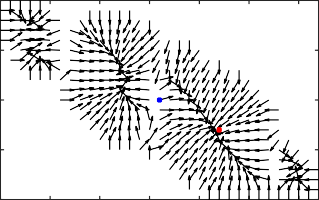

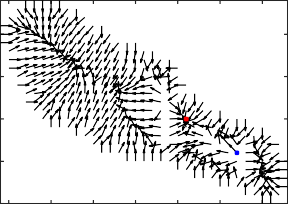

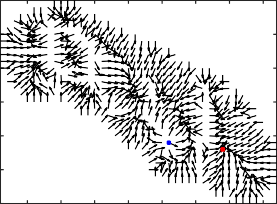


ℓ = 0 ℓ = 1 ℓ = -1 ℓ = 0’

Figure S 24: Different Topologies observed in CBNO Bragg Electronic density as a function of applied toplogical charge.


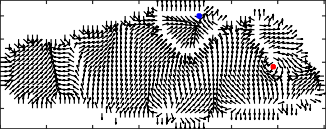

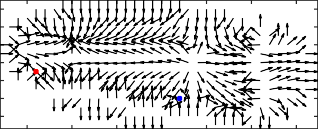

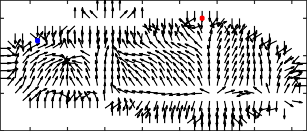

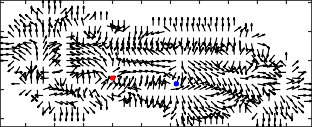
XY Plane - Slice 93

XZ Plane - Slice 91


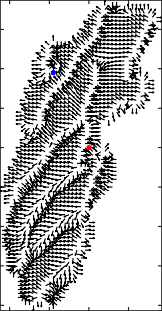

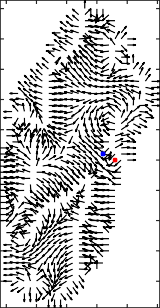

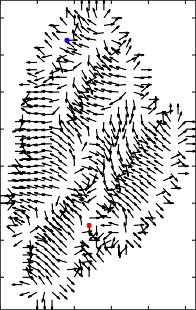

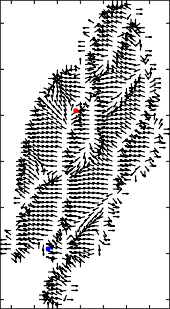


YZ Plane - Slice 66


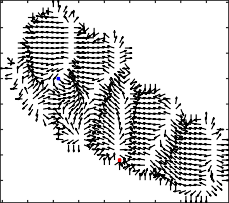

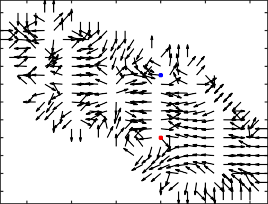

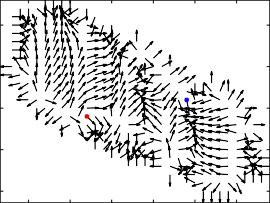

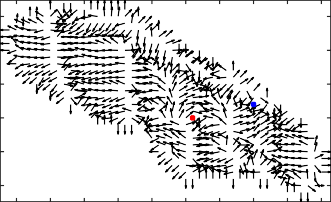


ℓ = 0 ℓ = 1 ℓ = -1 ℓ = 0’

Figure S 25: Different Topologies observed in CBNO Displacement Field as a function of applied toplogical charge. Blue dot indicates Vortex field and red dot indicates an anti-vortex topology.


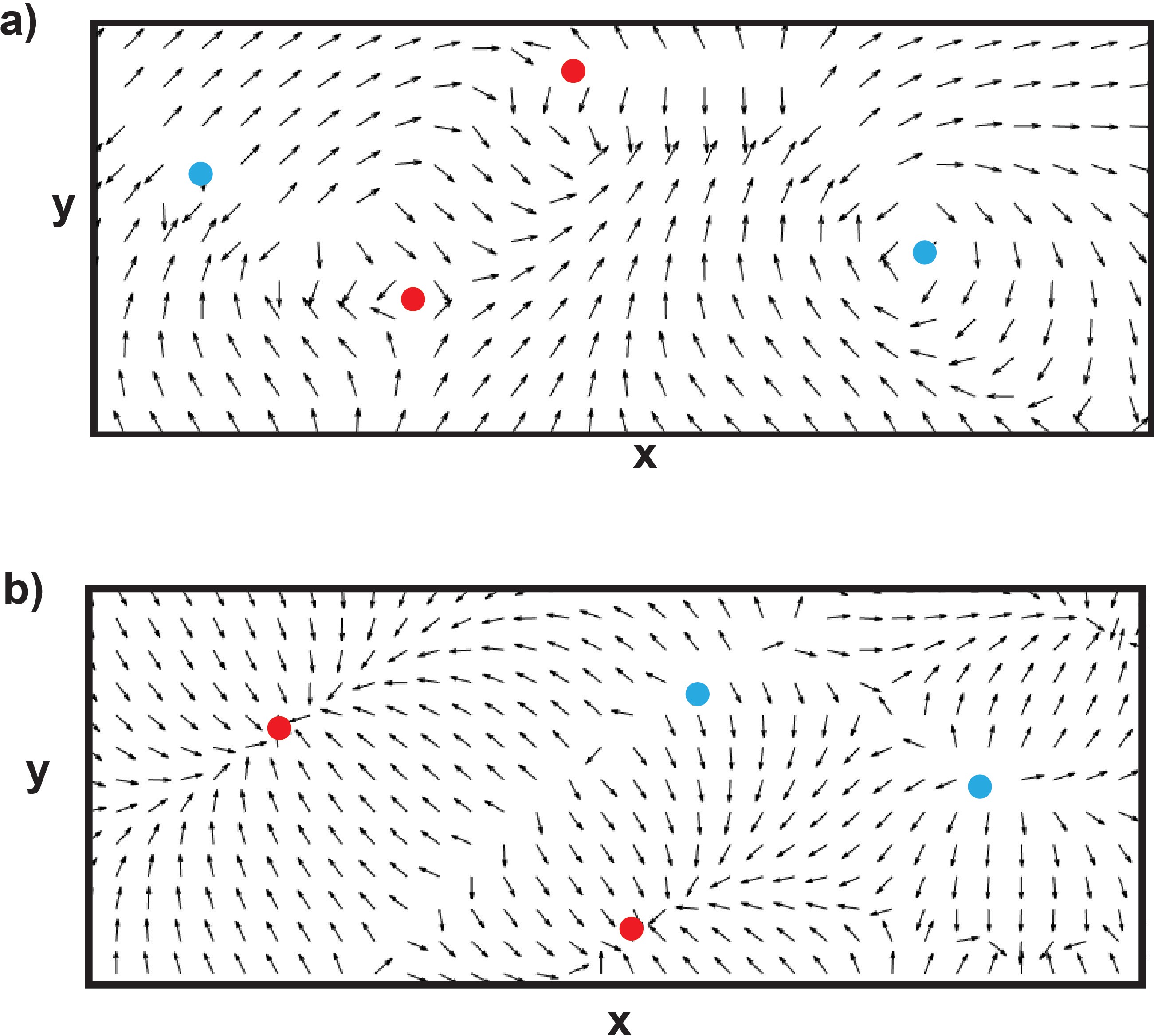


Figure S 26: Tracking Topologies in CBNO. A zoomed in view of the Topologies in CBNO. **a)** Displacement Field Topologies. **b)**

Bragg Electronic Density Topologies


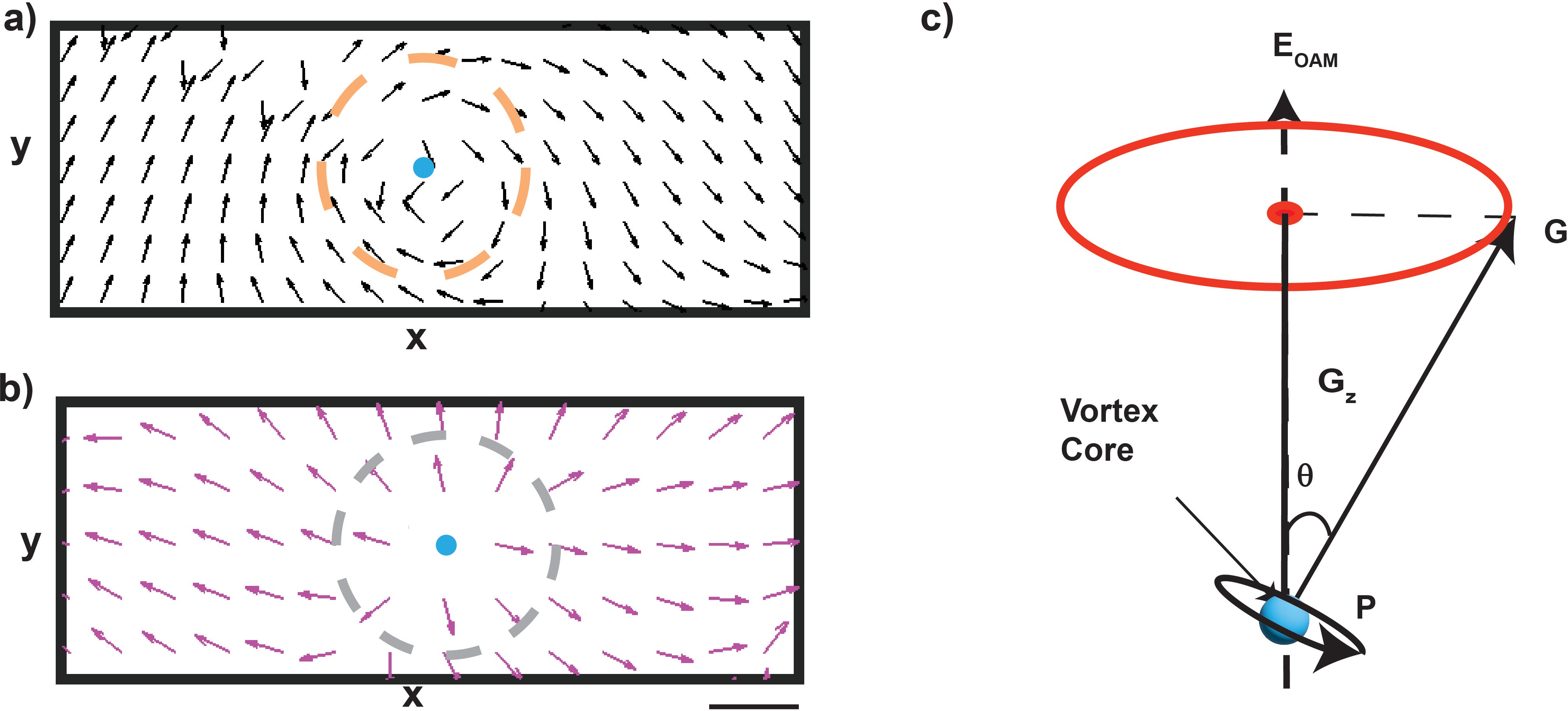


Figure S 27: Toroidal Moment Calculation from Bragg Electronic density and Displacement Field. **a)** Displacement Field Vortex Field,

**b)** Bragg Electronic Density Vortex Field, **c)** Gyration of the vortex field toroidal moment upon application of twisted light


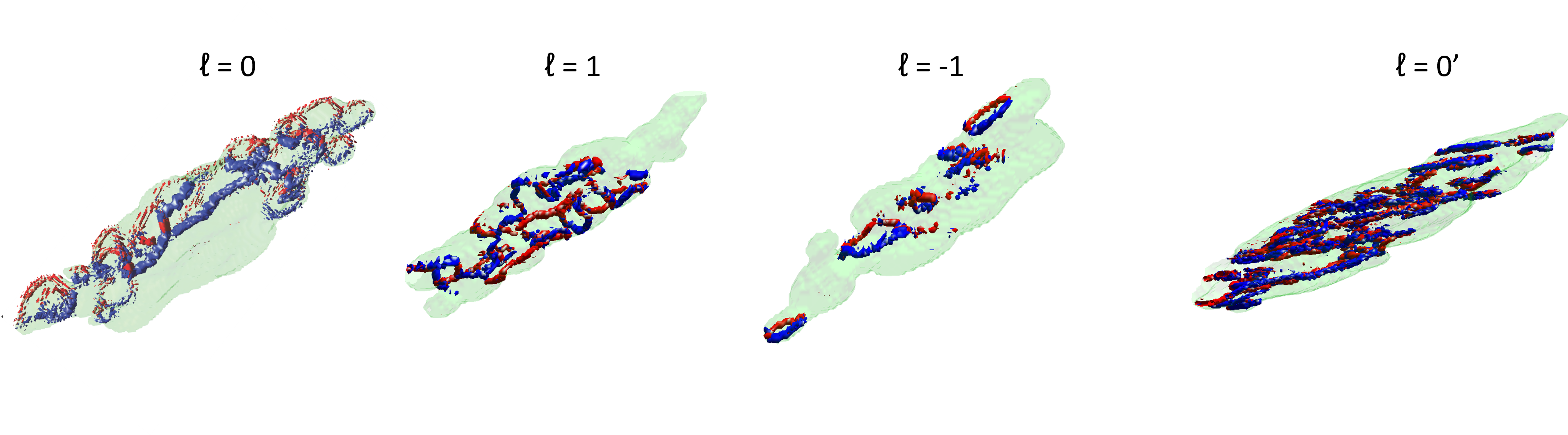


Figure S 28: Different Topologies observed in CBNO Toroidal Moment as a function of applied toplogical charge.


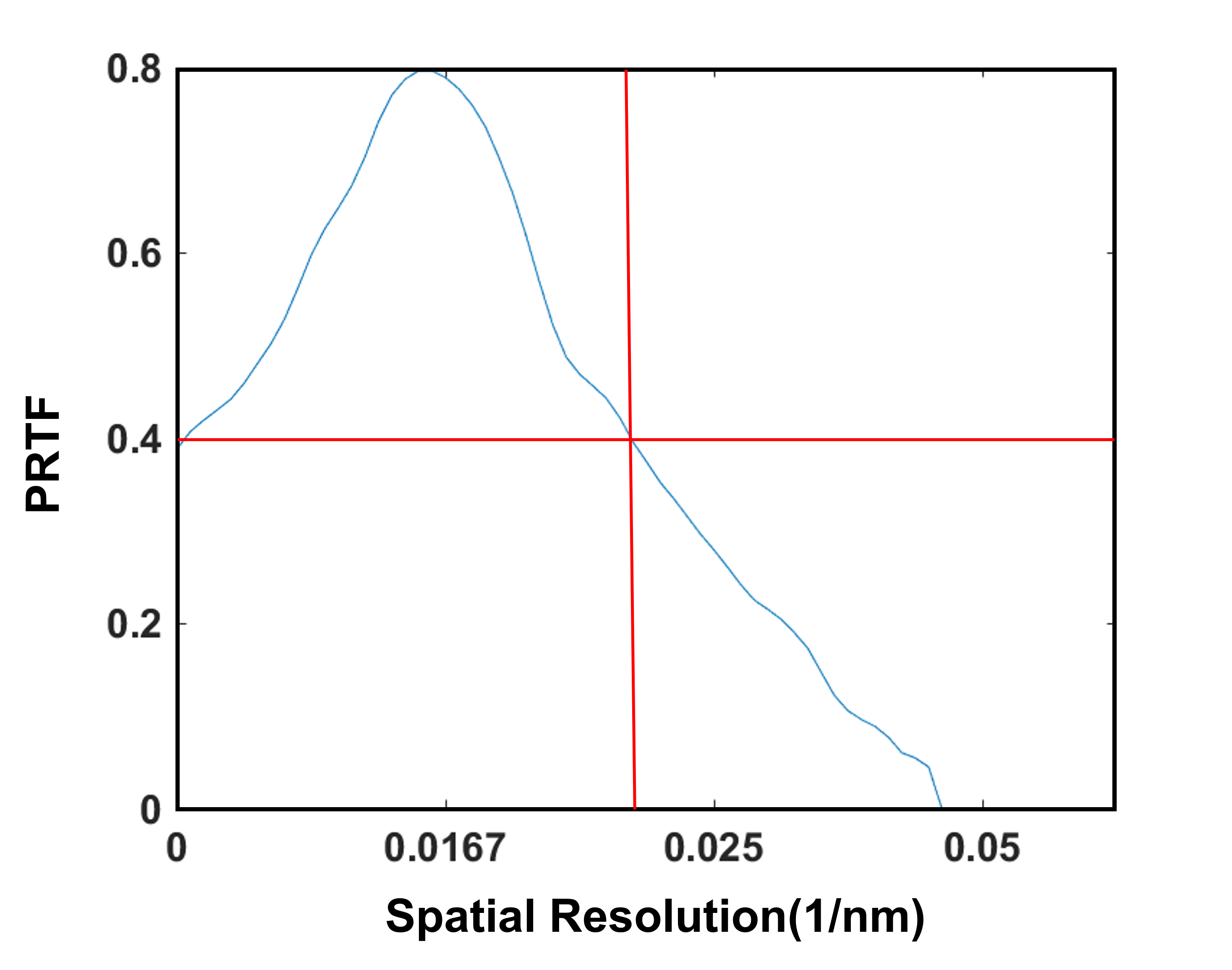


Figure S 29: Phase Retrieval Transfer Function (PRTF) showing a spatial resolution is indicated by the vertical red line is about 33 nm.


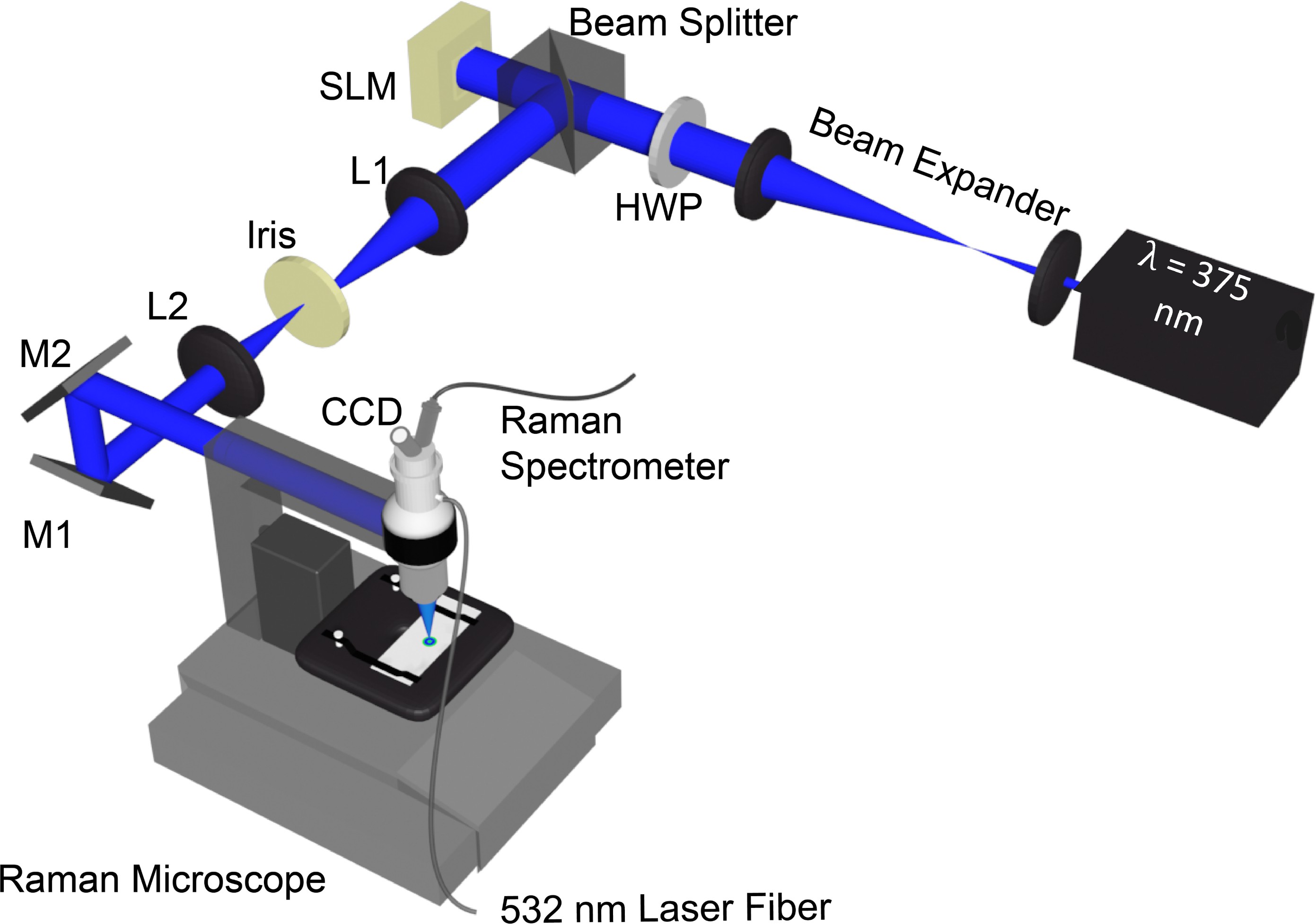


Figure S 30: Schematic of the Raman Experimental Set-up


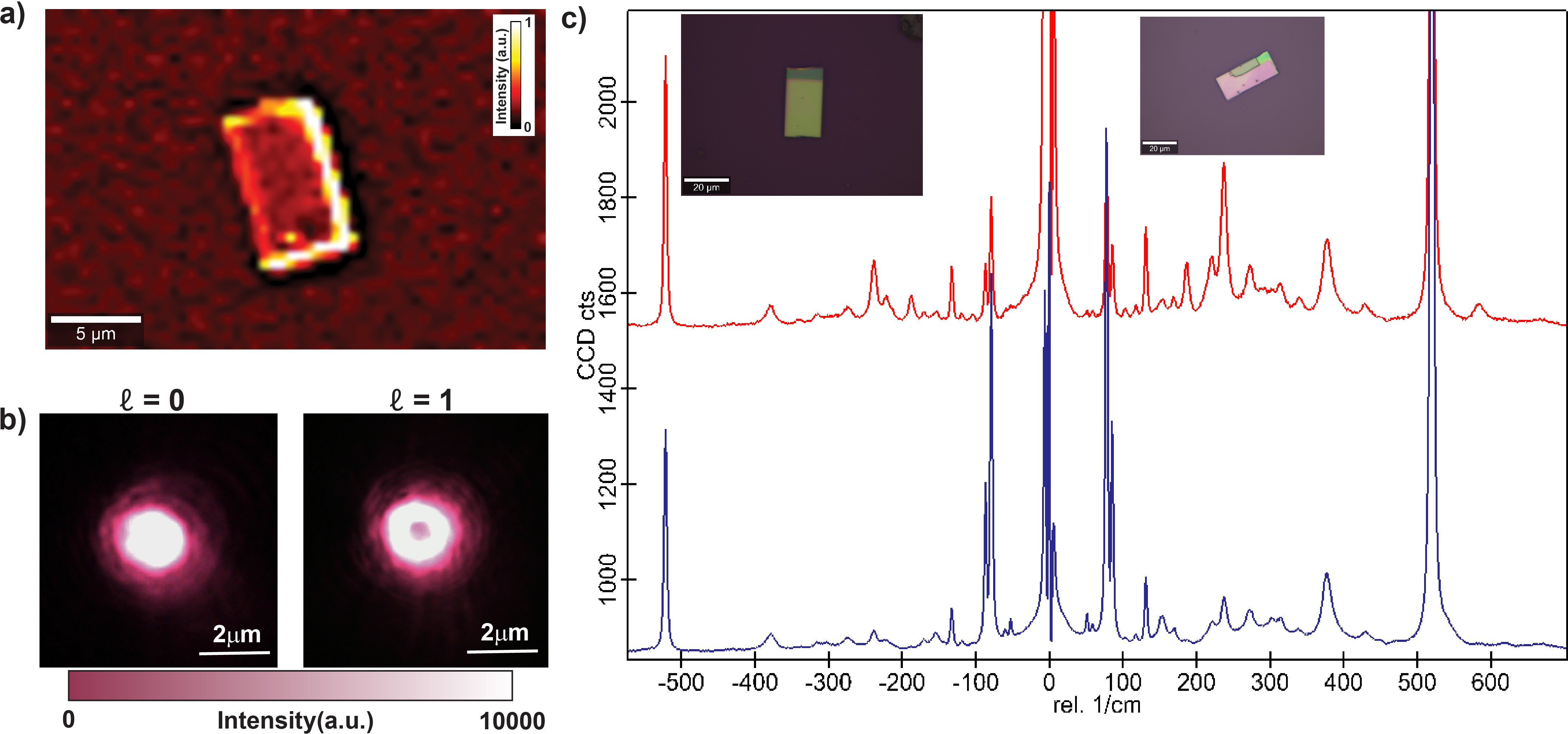


Figure S 31: Reference Raman Data and Imaging Twisted Light.**a)** Reference Raman Map of the in-plane Bi-O phonon frequency mode of the CBNO flake used in Raman Studies. **b)** Intensity images of the *ℓ* = 0 & *ℓ* = 1 respectively. **c)** CBNO nanoflake reference scans without any external stimulii.





Figure S 32: DFT modeling of strain-induced shifts in Raman Spectra of Reference i.e. unperturbed CBNO unit cell and a unit cell under the strain from TL with a topological charge of *ℓ* = 1.


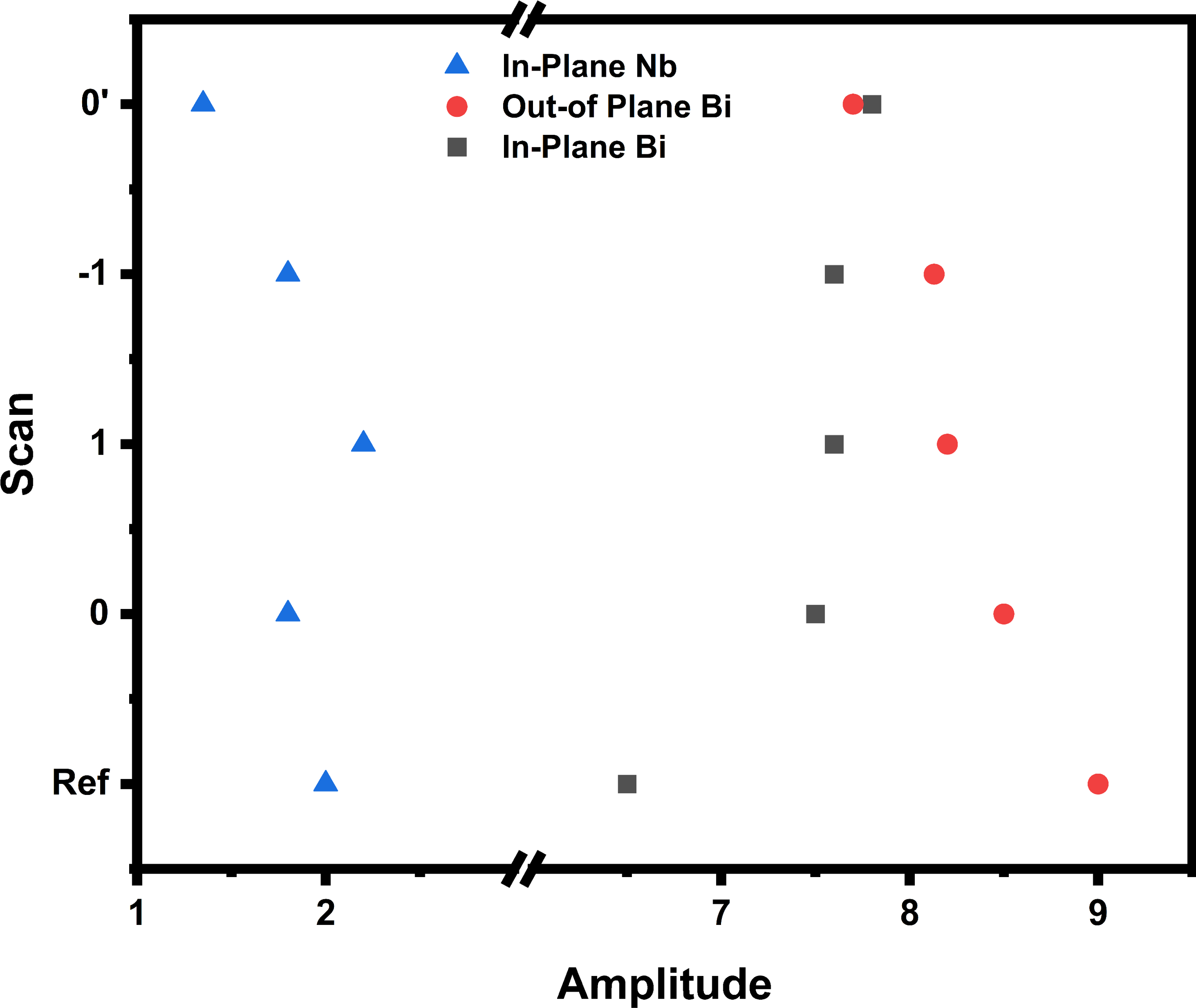


Figure S 33: Variation in Amplitude of the observed spectra as a function of applied topological charge


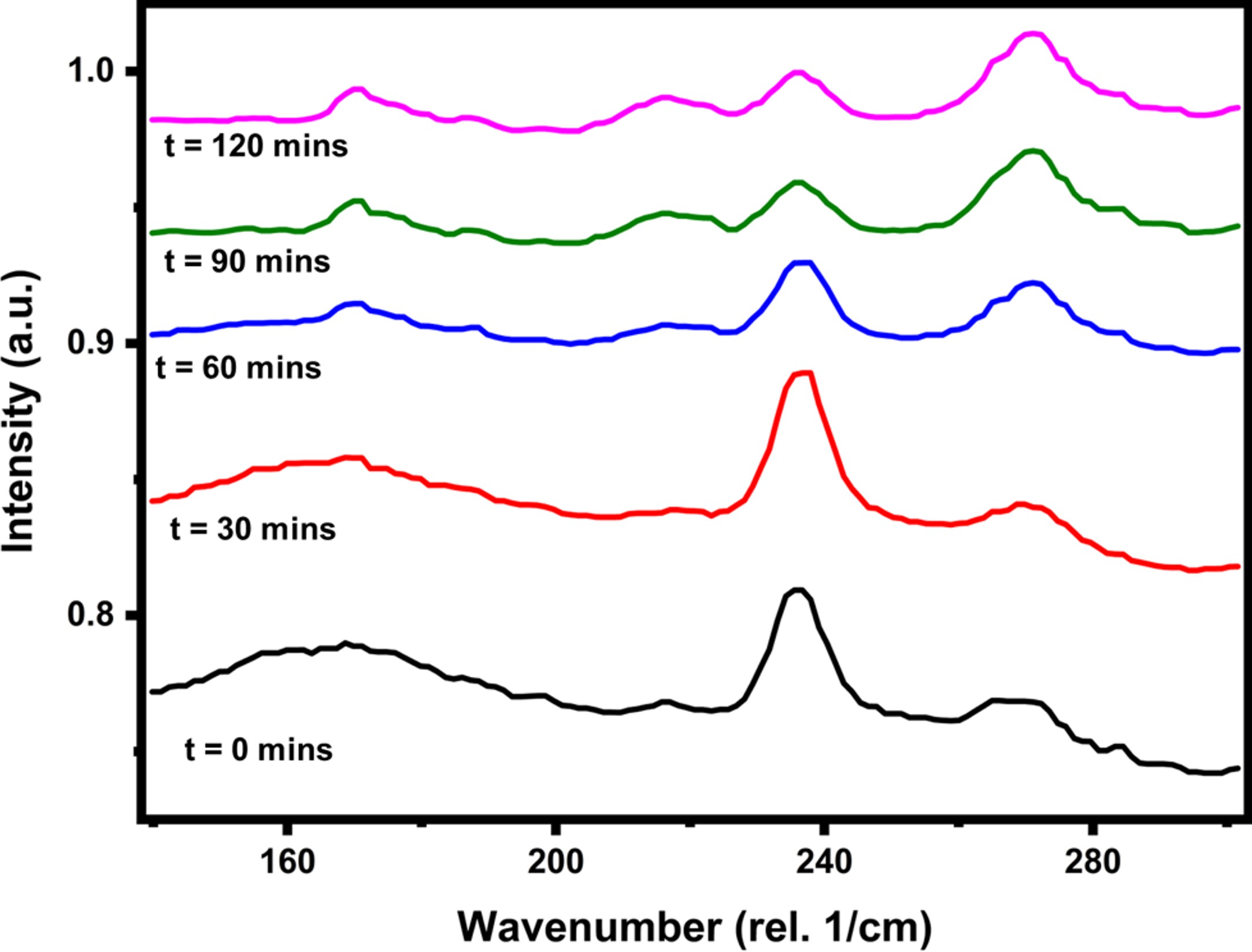


Figure S 34: Raman Scan depicting Stability of the flakes illuminated with gaussian beam.


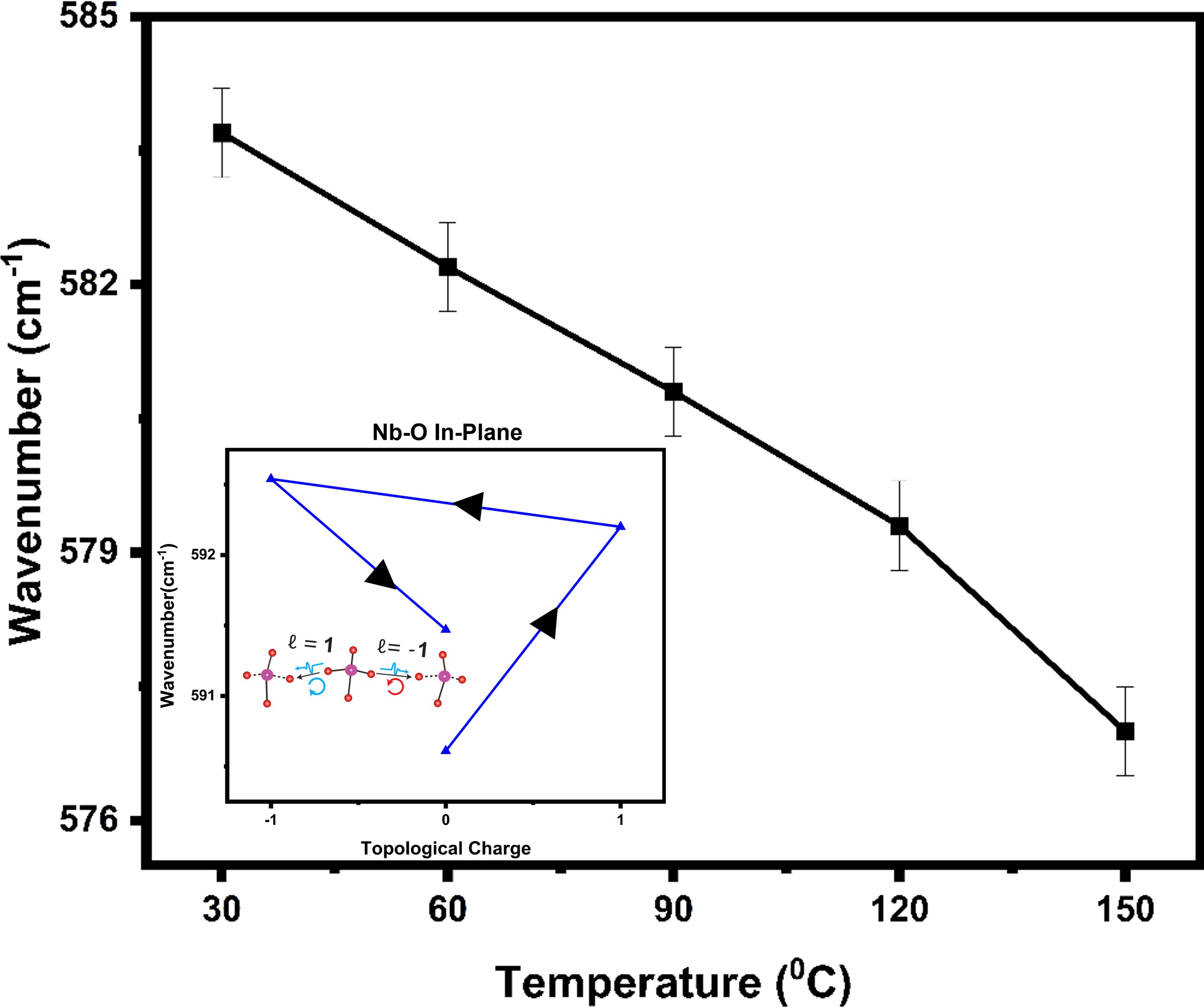


Figure S 35: Raman Peak profile analysis as a function of temperature for the Nb-O in-plane bonds. The inset shows a comparison with the effect of twisted light on the same Nb-O in-plane bond.


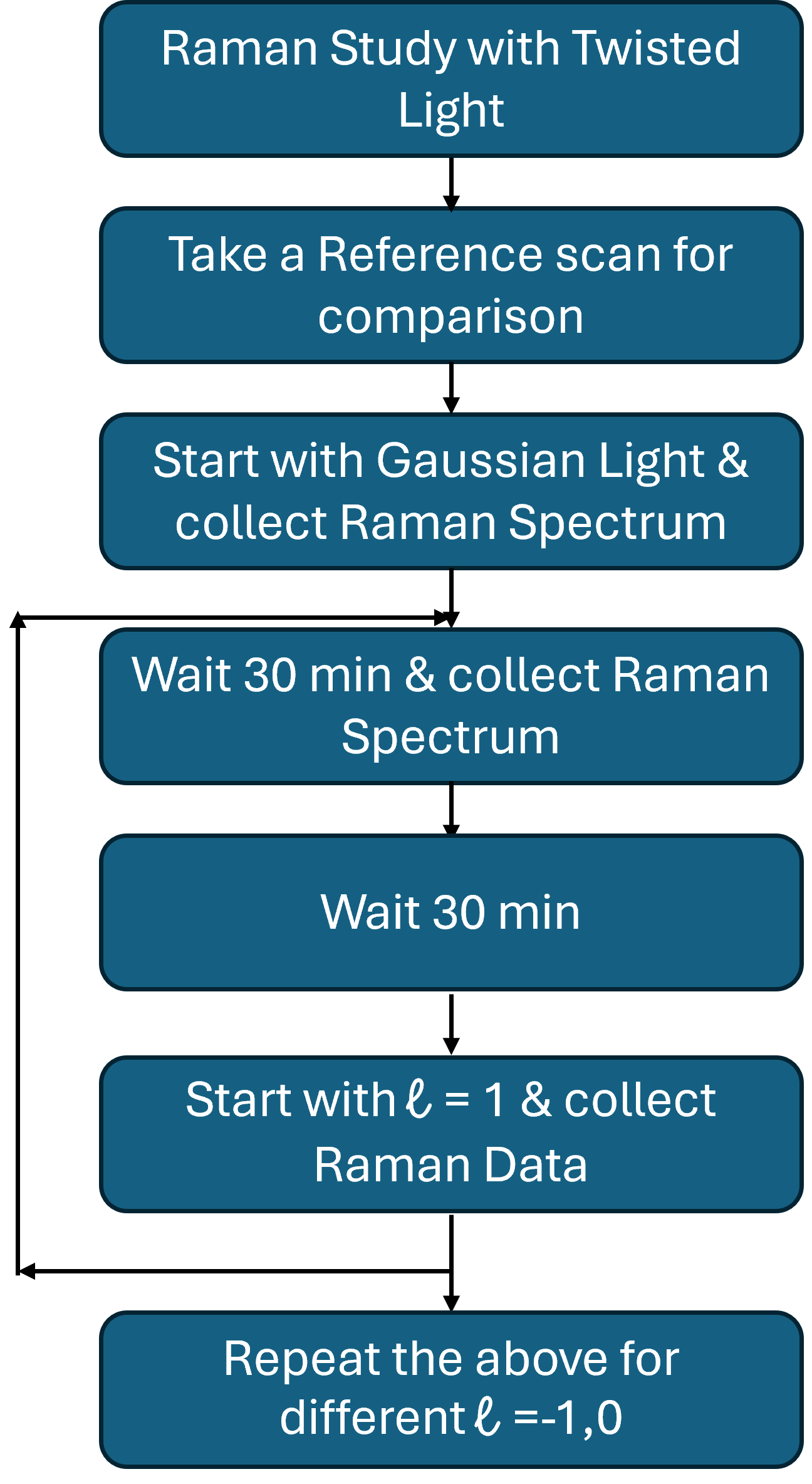


Figure S 36: Flowchart depicting the algorithm for twisted light control of the ferroelectric topologies in CBNO


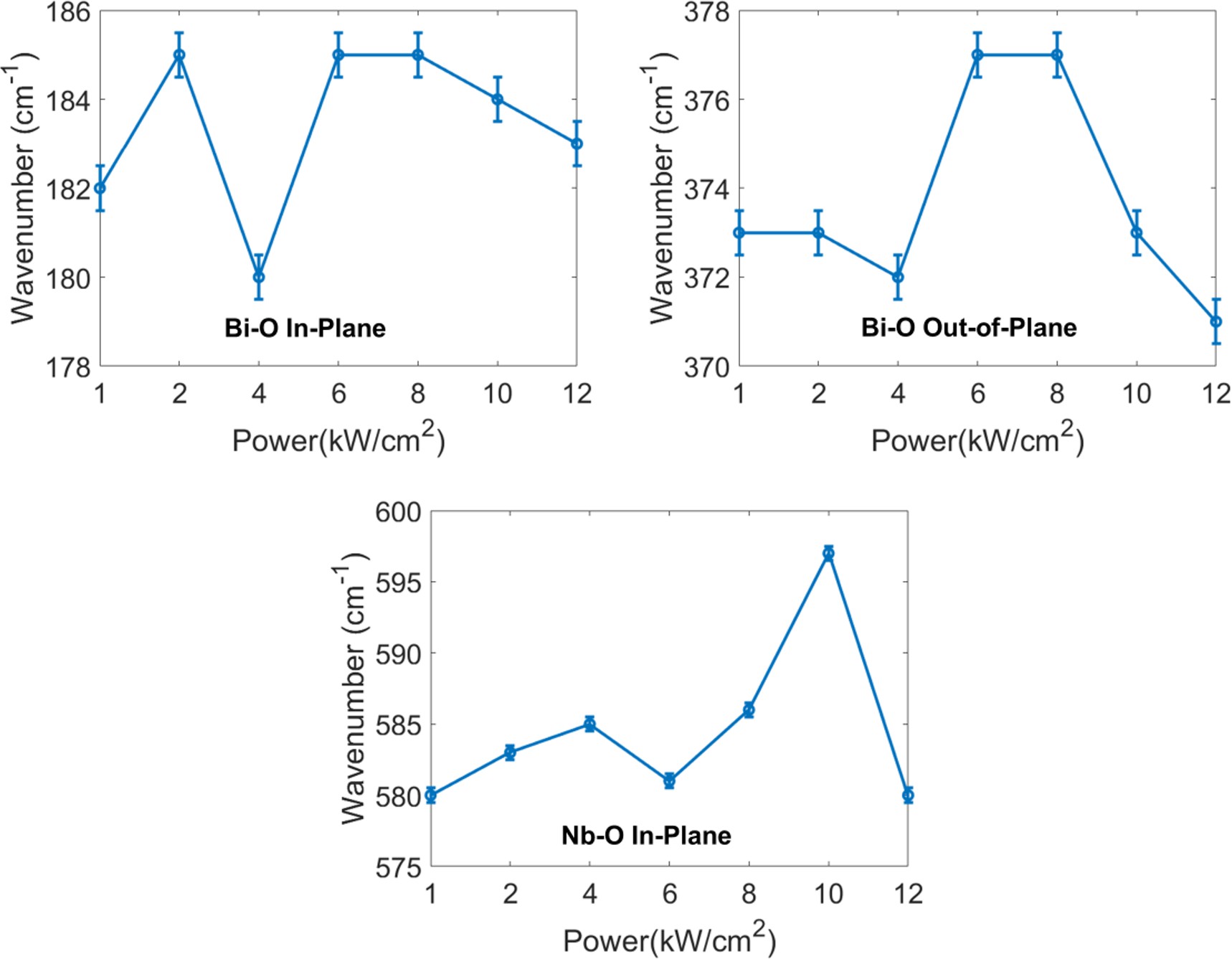


Figure S 37: Peak profile tracking for power/intensity dependent Raman Spectra for UV Twisted light for different in plane and out-of- plane bonds important for ferroelectric landscape in CBNO. The error bars are ± 0.5 1/cm.


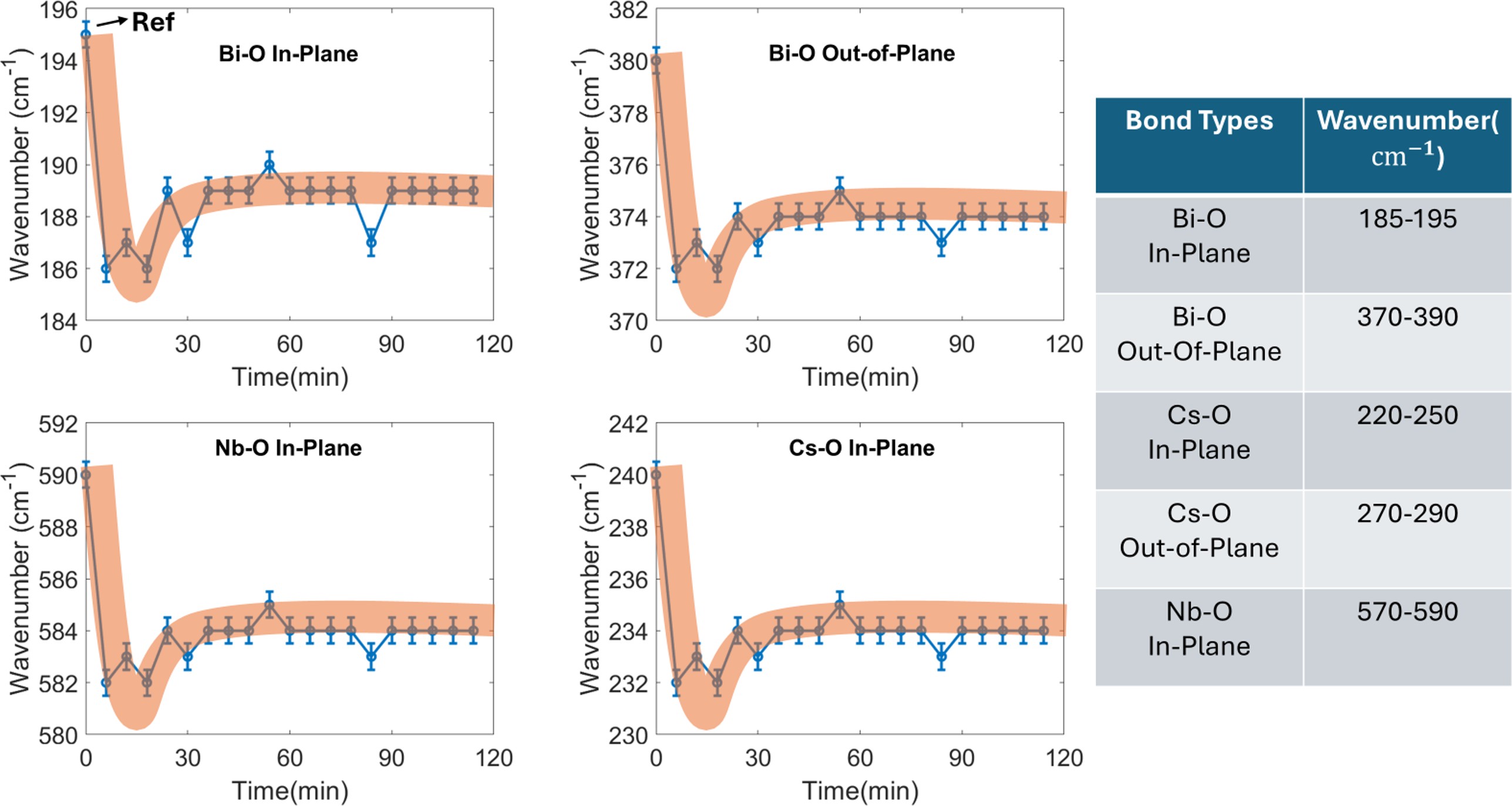


Figure S 38: Control Measurement for stability under UV light. Tracking Peak positions for different bonds where 0 min indicates a reference scan after which the UV laser was switched on immediately and the scans were collected in intervals of 6 minutes. The error bars are ± 0.5 1/cm.

1. Guo et al., ‘Unit-Cell-Thick Domain in Free-Standing Quasi-Two-Dimensional Ferroelectric Material’. [↑](#endnote-ref-1)
2. Duan, Lü, and Wang, ‘Propagation of Hermite–Gaussian and Laguerre–Gaussian Beams beyond the Paraxial Approximation’. [↑](#endnote-ref-2)
3. Allen, Padgett, and Babiker, ‘IV The Orbital Angular Momentum of Light’. [↑](#endnote-ref-3)
4. Kiselev and Plutenko, ‘Optical Trapping by Laguerre-Gaussian Beams’. [↑](#endnote-ref-4)
5. Kim and Lee, ‘Hermite–Gaussian and Laguerre–Gaussian Beams beyond the Paraxial Approximation’. [↑](#endnote-ref-5)
6. Kovalev, Kotlyar, and Porfirev, ‘Optical Trapping and Moving of Microparticles by Using Asymmetrical Laguerre–Gaussian Beams’. [↑](#endnote-ref-6)
7. Segall et al., ‘First-Principles Simulation’. [↑](#endnote-ref-7)
8. Giannozzi et al., ‘QUANTUM ESPRESSO’. [↑](#endnote-ref-8)
9. Perdew, Burke, and Ernzerhof, ‘Generalized Gradient Approximation Made Simple’. [↑](#endnote-ref-9)
10. Monkhorst and Pack, ‘Special Points for Brillouin-Zone Integrations’. [↑](#endnote-ref-10)
11. Baroni et al., ‘Phonons and Related Crystal Properties from Density-Functional Perturbation Theory’. [↑](#endnote-ref-11)
12. Geyer et al., ‘Chirality-Induced Spin Selectivity in a Coarse-Grained Tight-Binding Model for Helicene’. [↑](#endnote-ref-12)
13. Karpov et al., ‘Nanoscale Topological Defects and Improper Ferroelectric Domains in Multiferroic Barium Hexaferrite Nanocrystals’. [↑](#endnote-ref-13)
14. Karpov et al., ‘Three-Dimensional Imaging of Vortex Structure in a Ferroelectric Nanoparticle Driven by an Electric Field’. [↑](#endnote-ref-14)
15. Fienup, ‘Phase Retrieval Algorithms’. [↑](#endnote-ref-15)
16. Vanderbilt and King-Smith, ‘Electric Polarization as a Bulk Quantity and Its Relation to Surface Charge’. [↑](#endnote-ref-16)
17. Prosandeev et al., ‘Ferroelectric Vortices and Related Configurations’. [↑](#endnote-ref-17)
18. Wang et al., ‘Entangled Polarizations in Ferroelectrics’. [↑](#endnote-ref-18)
19. Jiang et al., ‘Giant Pyroelectricity in Nanomembranes’. [↑](#endnote-ref-19)
